# Supplementary material for: Sexual and reproductive health and human rights of women living with HIV
Source: J Int AIDS Soc. 2015 Dec 1;18(6Suppl 5):20834. doi: 10.7448/IAS.18.6.20834 (PMC4813610; doi:10.7448/IAS.18.6.20834)
Supplement: Related texts

# Sub article: Advancing the sexual and reproductive health and human rights of women living with HIV

**DOI:** 10.7448/IAS.18.6.20760

**Published:** 2015-12-01

**Authors:** Mona Loutfy, Rajat Khosla, Manjulaa Narasimhan

**Affiliations:** 1 Women's College Research Institute, Women's College Hospital, University of Toronto, Toronto, Ontario, Canada; 2 Department of Reproductive Health and Research, World Health Organization, Geneva, Switzerland

**Keywords:** HIV, women's health, sexual health, reproductive health, reproductive rights, gender, equity, human rights

## Abstract

**Introduction:**

Many women living with HIV can have safe, healthy and satisfying sexual and reproductive health, but there is still a long way to go for this to be a reality, especially for the most vulnerable amongst them who face repeated violations of their rights.

**Discussion:**

The contributions in this Supplement from researchers, clinicians, programme managers, policy makers, and women living with HIV demands an important appreciation that the field of sexual and reproductive health and human rights for women living with HIV is complex on many levels, and women living with HIV form a very diverse community.

**Conclusions:**

The manuscripts emphasize that attention must be paid to the following critical dimensions: 1) Placing human rights and gender equality at the centre of a comprehensive approach to health programming, in particular in relation to sexuality and sexual health; 2) Ensuring health systems responsiveness to minimizing inequalities in access to health care and quality of care that often do not meet the needs of women living with HIV; 3) Engaging and empowering women living with HIV in the development of policies and programmes that affect them; and 4) Strengthening monitoring, evaluation and accountability procedures to provide good quality data and ensuring remedies for violations of health and human rights of women living with HIV.

## References

[1] GIPA – The Greater Involvement of People Living with HIV/AIDS (1994). The Paris Declaration. Paris AIDS Summit [Internet].

[2] Amin A (2015). Addressing gender inequalities to improve the sexual and reproductive health and wellbeing of women living with HIV. J Int AIDS Soc.

[3] Khosla R, Van Belle N, Temmerman M (2015). Advancing the sexual and reproductive health and human rights of women living with HIV: a review of UN, regional and national human rights norms and standards. J Int AIDS Soc.

[4] Kumar S, Gruskin S, Khosla R, Narasimhan M (2015). Human rights and the sexual and reproductive health of women living with HIV – a literature review. J Int AIDS Soc.

[5] Kaida A, Carter A, de Pokomandy A, Patterson S, Proulx-Boucher K, Nohpal A (2015). Sexual inactivity and sexual satisfaction among women living with HIV in Canada in the context of growing social, legal and public health surveillance. J Int AIDS Soc.

[6] Huchko MJ, Maloba M, Nakalembe M, Cohen CR (2015). The time has come to make cervical cancer prevention an essential part of comprehensive sexual and reproductive health services for HIV-positive women in low-income countries. J Int AIDS Soc.

[7] Heffron R, Davies N, Cooke I, Kaida A, Mergler R, van der Poel S (2015). A discussion of key values to inform the design and delivery of services for HIV-affected women and couples attempting pregnancy in resource-constrained settings. J Int AIDS Soc.

[8] Matheson R, Moses-Burton S, Hsieh AC, Dilmitis S, Happy M, Sinyemu E (2015). Fundamental concerns of women living with HIV around the implementation of Option B+. J Int AIDS Soc.

[9] Orza L, Bewley S, Logie CH, Crone ET, Moroz S, Strachan S (2015). How does living with HIV impact on women's mental health? Voices from a global survey. J Int AIDS Soc.

[10] Orza L, Bewley S, Chung C, Crone ET, Nagadya H, Vazquez M (2015). “Violence. Enough already”: findings from a global participatory survey among women living with HIV. J Int AIDS Soc.

[11] (2015). Building a Safe House on Firm Ground [Internet].

[12] Kennedy CE, Haberlen S, Amin A, Baggaley R, Narasimhan M (2015). Safe disclosure of HIV serostatus for women living with HIV who experience or fear violence: a systematic review. J Int AIDS Soc.

[13] Chandra-Mouli V, Armstrong A, Amin A, Ferguson J (2015). A pressing need to respond to the needs and sexual and reproductive health problems of adolescent girls living with HIV in low and middle income countries. J Int AIDS Soc.

[14] World Health Assembly (2015). Women and health: 20 years of the Beijing Declaration and Platform for Action. A68/16 [Internet].

# Sub article: Addressing gender inequalities to improve the sexual and reproductive health and wellbeing of women living with HIV

**DOI:** 10.7448/IAS.18.6.20302

**Published:** 2015-12-01

**Authors:** Avni Amin

**Affiliations:** Department of Reproductive Health and Research, World Health Organization, Geneva, Switzerland

**Keywords:** gender inequalities, stigma, discrimination, laws, sexual and reproductive health

## Abstract

**Introduction:**

Globally, women constitute 50% of all persons living with HIV. Gender inequalities are a key driver of women's vulnerabilities to HIV. This paper looks at how these structural factors shape specific behaviours and outcomes related to the sexual and reproductive health of women living with HIV.

**Discussion:**

There are several pathways by which gender inequalities shape the sexual and reproductive health and wellbeing of women living with HIV. First, gender norms that privilege men's control over women and violence against women inhibit women's ability to practice safer sex, make reproductive decisions based on their own fertility preferences and disclose their HIV status. Second, women's lack of property and inheritance rights and limited access to formal employment makes them disproportionately vulnerable to food insecurity and its consequences. This includes compromising their adherence to antiretroviral therapy and increasing their vulnerability to transactional sex. Third, with respect to stigma and discrimination, women are more likely to be blamed for bringing HIV into the family, as they are often tested before men. In several settings, healthcare providers violate the reproductive rights of women living with HIV in relation to family planning and in denying them care. Lastly, a number of countries have laws that criminalize HIV transmission, which specifically impact women living with HIV who may be reluctant to disclose because of fears of violence and other negative consequences.

**Conclusions:**

Addressing gender inequalities is central to improving the sexual and reproductive health outcomes and more broadly the wellbeing of women living with HIV. Programmes that go beyond a narrow biomedical/clinical approach and address the social and structural context of women's lives can also maximize the benefits of HIV prevention, treatment, care and support.

## Introduction

Globally, women constitute half of all persons living with HIV. In sub-Saharan Africa, the region with the highest burden of HIV, women constitute 57% of persons living with HIV; and adolescent girls and young women are twice as likely to be living with HIV as compared to boys and young men. In low- and middle-income countries, female sex workers are 13.5 times more likely to be living with HIV as compared to the general population of women in reproductive age groups [1]. Globally, transgender women are 49 times more likely to be living with HIV as compared to all adults of reproductive age groups [2–4].

The sexual and reproductive health needs of women living with HIV require particular attention because these women are disproportionately vulnerable to certain reproductive health problems as compared to HIV-negative women and also in relation to the prevention of vertical transmission of HIV. Studies show that, as with women who are HIV negative, women living with HIV have high rates of unintended pregnancy and low rates of contraceptive use including condom use [5–9]. In sub-Saharan Africa, women living with HIV are significantly more likely to die during pregnancy or the postpartum period as compared to HIV-negative women [10,11]. Globally, women living with HIV are also more likely to have a higher incidence and progression of cervical neoplasia as compared to women who are HIV negative [12].

There has been increasing attention given to certain aspects of reproductive health of women living with HIV, particularly in the context of preventing vertical transmission of HIV. However, much of this focus has largely addressed the biomedical/clinical and health systems factors [13–15]. There has been less attention to a more holistic response that goes beyond disease prevention and addresses the sexual, emotional and mental health as well as social and economic wellbeing of women living with HIV as a legitimate focus of programming and research in its own right [16,17]. This state of affairs stands in stark contrast to what women living with HIV have articulated as their needs and priorities. These needs include the importance of addressing gender inequalities, violence against women, financial security and social support, reproductive health beyond pregnancy, and sexuality in a positive framework [18]. The UNAIDS Gap Report [3] highlights women living with HIV as one of the 12 priority populations. The report identifies stigma and discrimination, gender inequalities, and punitive laws and policies as three of the top four reasons for their vulnerability.

Nearly two decades of research and programming have highlighted that gender inequalities are a key structural driver of women's vulnerability to acquiring HIV. The importance of addressing gender inequalities is well recognized in key global commitments to ending HIV. Some countries are beginning to address them as part of their national HIV and AIDS responses [19,20]. However, concrete actions on a significant scale and in a sustained manner with concomitant resources are yet to materialize. The pathways by which gender inequalities shape women's risk of acquiring HIV are increasingly being mapped out, particularly as they relate to the intersections of intimate partner violence and HIV [21–23]. There is a small, but increasing body of evidence on interventions that work to address gender inequalities as a structural driver of women's risk of becoming infected with HIV, such as those that promote egalitarian gender norms, empower women and girls economically and in their sexual and reproductive decision-making, and reduce violence against women [23–27].

While gender inequalities affect HIV-negative women as well as women living with HIV in many similar ways, the latter face unique challenges related to stigma and discrimination, as well as pressures related to their sexual and childbearing decisions, economic security, mental health and emotional wellbeing. This paper describes how gender inequalities shape the sexual and reproductive health and wellbeing of women living with HIV, specifically via the following pathways: (1) unequal power relations, harmful gender norms and violence against women; (2) women's unequal access to and control over economic resources; (3) stigma and discrimination; and (4) punitive laws and gender-discriminatory policies. These pathways are examined in terms of four interrelated outcomes: (1) disclosure of HIV status; (2) ability to have safe and pleasurable sex; (3) fulfilment of fertility intentions and enabling of reproductive choices; and (4) management of treatment. The concept of wellbeing is included to underscore the importance of considering mental and emotional health as well as social and economic factors.

## Unequal power relations in sexual and reproductive decision-making: the role of harmful gender norms and violence against women

In many settings, gender norms privilege men's control over women or perpetuate unequal power relations. These norms prevent women from having autonomy in sexual and reproductive health decisions. Surveys of women of reproductive age (e.g. demographic and health surveys) show that in many settings, a large proportion of married women, especially young women, do not have a final say in their own healthcare decisions [3,28,29]. Analysis of sexual behaviours of women and men from surveys shows that in general, married women find negotiation of safer sex and condom use much more difficult than do single women [30].

In many societies, women living with HIV, like others, face tremendous social pressures to bear children. Women gain status and their worth is proven through their fertility. Research highlights the importance of partners’ dominance in decision-making with respect to condom use and desire for children in shaping the sexual and reproductive decisions of women living with HIV as well as in the uptake of prevention of mother-to-child HIV transmission (PMTCT) services [31–34]. Hence, women, including those living with HIV, face pressures to have unprotected sex in order to conceive or are unable to use contraception because of such social norms [18,35–37].

Gender norms related to sexuality confer different expectations for women and men to have consensual sex [38–40]. For women, a central issue is that of freedom from violence, which is a stark expression of men's power, control and entitlement over women. Globally 30% of women have experienced physical and/or sexual violence by an intimate partner in their lifetime [41,42]. Data show that intimate partner violence against women is associated with a 1.5-fold increase in risk of sexually transmitted infections (STIs) or HIV in some regions [41]. Data on prevalence of intimate partner violence among women living with HIV are not easily obtained. However, one systematic review of studies from the United States of America highlighted a higher proportion of women living with HIV experiencing partner violence as compared to women in the general population [43]. A large body of studies from sub-Saharan Africa show that women's fear or experience of violence are a major barrier to HIV disclosure [44,45]. Studies on HIV disclosure outcomes among women living with HIV show that rates of negative outcomes, including violence, range from 3 to 15% and up to 59% in a couple of studies [46–48]. Studies also show an association between partner violence and lower uptake of PMTCT, continued or increased sexual risk behaviours and poor adherence to antiretroviral therapy – in part explained by stress, poor mental health, and a lack of control over health-promoting behaviours [43,49–53].

## Unequal access to and control over economic resources: the role of food insecurity and lack of property and inheritance rights

An increasing number of studies highlight that, while antiretroviral therapy (ART) access has improved, there continue to be socio-economic barriers to uptake of and adherence to treatment. Food insecurity has been identified as a key barrier to ART adherence and quality of life for people living with HIV by a number of studies [54–56]. Women are disproportionately susceptible to food insecurity because of their lack of access to and control over economic resources in the form of ownership of land, assets and other property, and their lower access to formal employment than men. Research from sub-Saharan Africa and South Asia highlights how women living with HIV are denied their property and inheritance rights by relatives when their husbands die due to HIV-related conditions [57–60].

This denial of land and property rights contributes to food insecurity, which in turn increases sexual risk taking (e.g. transactional or commercial sex) and limits women's ability to leave abusive relationships. For example, a study from Swaziland and Botswana highlighted that food insecurity among women was associated with significantly higher odds of inconsistent condom use with a non-primary partner, transactional sex and lack of control in sexual relationships, but that these associations were weaker among men [61]. Similar findings were shown in a qualitative study on food insecurity among women living with HIV in Uganda [62]. Studies also highlight women's economic dependency and their fear of being abandoned as a barrier to HIV disclosure [44,45,63,64]. Other adverse consequences of food insecurity on women living with HIV are in relation to their increased nutritional and energy requirements during pregnancy and lactation as well as the increased stress and burden on them to procure food and clean water for family members, including children who may also be living with HIV [65–67].

## Stigma and discrimination

Stigma and discrimination are among key barriers that women living with HIV face in achieving their sexual and reproductive health. While all those who are living with HIV can face stigma because of judgments made about their behaviours by families and communities, women are more likely to be blamed because many societies have different expectations and standards for women's sexual conduct than for men's [68,69]. Moreover, in sub-Saharan Africa, as women are more likely to be tested first in the context of PMTCT programmes, they are also more likely to be blamed for bringing HIV into the family [44,45,70]. This potential consequence is likely not only to affect women's willingness to disclose their HIV status, but also to compromise their safety due to threats or experience of violence. Some women living with HIV report rejection of sexual relations by their partners or inability to find sexual partners because of their HIV status [18,71]. Women living with HIV may also experience internalized stigma that includes fear and anxiety that partners may not find them attractive [70,72,73]. In some settings, HIV programme staff discourage women living with HIV to have sex or blame them as being irresponsible if they have unprotected sex, which can affect their sexual, emotional and mental health and wellbeing [74,75].

For a number of women living with HIV who want children, there are pressures from institutions such as healthcare to not bear children [76]. Data from Bangladesh, the Dominican Republic and Ethiopia show that between a quarter to nearly half of all women living with HIV were advised by health workers to not have children [77]. Reports of women living with HIV being coerced into sterilizations have occurred in several settings (e.g. Bangladesh, Chile, Dominican Republic, Honduras, El Salvadaor, Mexico, Nicaragua and Namibia) [3,78]. Several countries surveyed as part of the stigma index (i.e. a survey-based tool to assess or measure levels of stigma experienced by people living with HIV) reported the proportion of women living with HIV who were denied family planning services in the last 12 months to be at least 10% [4]. These data highlight the contradictory pressures that women living with HIV face in relation to their fertility intentions and reproductive choices. The enactment of these contradictory pressures on women by healthcare institutions violates their reproductive rights.

## Laws that criminalize HIV transmission and gender-discriminatory HIV policy responses

Laws that criminalize HIV transmission, exposure and non-disclosure are not only unjust and difficult to enforce, but make for poor public health practice and outcomes by disempowering those living with HIV and discouraging them from testing, accessing treatment programmes or disclosing their HIV status [79]. Despite this, 61 countries have adopted laws that criminalize HIV transmission, while prosecutions for non-disclosure, exposure and transmissions have been recorded in at least 49 countries [3]. These laws are being adopted in a context of rapid expansion of HIV testing of pregnant women through PMTCT programmes. In West and Central Africa, laws criminalize women who transmit HIV to the foetus or child. This puts women in an impossible quandary, given that many are unable to demand condom use or disclose their HIV status due to fears of violence or abandonment by their partners [79]. Data show that punitive laws and law enforcement practices related to sex work and injecting drug use also contribute to stigma, violence and other rights violations against women living with HIV from key populations [3,79].

HIV policies have often failed to take into account gender inequalities in ways that further contribute to discrimination against women. Such policies have also failed to address the reasons behind men's lower access to HIV services. For example, HIV testing and counselling and disclosure has a distinct gendered pattern and dimension [44,45,80]. In a number of countries, more women are tested and know their HIV status compared to men, particularly in the context of women's higher frequency of use of maternal and child health services [81]. Studies from sub-Saharan Africa show that masculine norms and stigma prevent men from seeking HIV testing services [82,83]. Men use their partners' HIV status as a proxy for their own [45]. At the same time, an increasing number of countries are putting in place partner notification policies [45]. Hence, the onus of disclosure is on women, even as it brings with it the risk of violence and other negative consequences.

A number of countries with the highest burden of HIV among women and children have started to implement lifelong ART (Option B +) for all pregnant and postpartum women living with HIV and their infants [81]. The public health rationale and benefits for implementing Option B+ have been well established [84]. However, there is less consideration of the implications of early initiation and lifelong treatment, regardless of CD4+ count, on women that takes into account the gendered realities of their daily lives [85]. Data from Malawi, South Africa and Tanzania suggest that, while women are motivated to initiate and adhere to ART during pregnancy and post-partum periods in order to prevent HIV transmission to their child, they are less motivated to continue thereafter [86–88]. Qualitative data from Malawi, Tanzania and Uganda suggest that women living with HIV appreciate the positive benefits of Option B+, including the ability to prevent transmission to their children and partners, their own improved health and reduced stigma. However, they raise concerns about treatment and adherence in relation to the following: the lack of food security and nutrition that is required to maintain treatment; the requirement to disclose their HIV status, especially for those who face or fear partner violence; lack of information, support and counselling; and the side effects of treatment [87,89].

## Conclusions

Addressing gender inequalities is central to improving the sexual and reproductive health outcomes and more broadly, the wellbeing of women living with HIV. Even as HIV prevention, treatment and care services for women living with HIV are being expanded and bringing many benefits, the context of gender inequalities is undermining these efforts. Figure 1 summarizes the pathways by which gender inequalities shape the sexual and reproductive health and wellbeing of women living with HIV.

**Figure 1:** Pathways explaining how gender inequalities shape the sexual and reproductive health and wellbeing of women living with HIV.

This paper highlights the importance of interventions for women living with HIV to promote egalitarian and non-violent norms along with equitable decision-making between women and men. It also highlights the importance of interventions to address economic inequalities that contribute to food insecurity, such as interventions that promote land, property and inheritance rights of women living with HIV. Stigma and discrimination, particularly in healthcare settings, needs to be addressed in order to support the reproductive choices of women living with HIV. Strong advocacy is needed to repeal laws that criminalize HIV transmission. Finally, it is not enough to design HIV policies from a narrow biomedical/clinical and health systems framework. Instead, policies must take into account the social and structural context of women's lives from the very inception, so that women living with HIV feel less isolated and are more empowered to make informed choices and decisions with respect to their health and wellbeing. The evidence for effective women-centred approaches is limited. One such example is a study to improve the sexual and reproductive health of Canadian women living with HIV. As part of this study, a framework was developed to identify the elements of a women-centred model of care that addresses their physical health needs (i.e. from a clinical and biomedical perspective) as well as their social, emotional, mental, spiritual and cultural needs more broadly. The framework considers gender along with other intersecting social inequalities. It highlights the needs of women living with HIV for safety, respect, acceptance, self-determination, access to social and other supportive services, tailored and culturally sensitive information, and peer support, among others [90]. While this model is being empirically tested in one setting, it needs to be further applied in low- and middle-income country settings. A more holistic social science research agenda is needed to provide women-centred services to women living with HIV and promote their sexual and reproductive health and wellbeing – one that is grounded in social justice and human rights.

## Author information

Avni Amin is a technical officer for violence against women at the WHO. She has worked on issues of gender equality, violence against women and their linkages to sexual and reproductive health and HIV for the last 18 years.

## References

[1] Baral S, Beyrer C, Muessig K, Poteat T, Wirtz AL, Decker MR (2012). Burden of HIV among female sex workers in low-income and middle-income countries: a systematic review and meta-analysis. Lancet Infect Dis.

[2] Baral SD, Poteat T, Stromdahl S, Wirtz AL, Guadamutz TE, Beyrer C (2013). Worldwide burden of HIV in transgender women: a systematic review and meta-analysis. Lancet Infect Dis.

[3] Joint United Nations HIV/AIDS Programme (UNAIDS) (2014). The gap report.

[4] Joint United Nations HIV/AIDS Programme (UNAIDS) (2013). Global report: UNAIDS report on the global AIDS epidemic 2013.

[5] McCoy SI, Buzdugan R, Ralph LJ, Mushavi A, Mahomva A, Hakobyan A (2014). Unmet need for family planning, contraceptive failure and unintended pregnancy among HIV-infected and HIV-uninfected women in Zimbabwe. PLoS One.

[6] Melaku YA, Zeleke EG (2014). Contraceptive utilization and associated factors among HIV positive women on chronic follow up care in Tigray region, Northern Ethiopia: a cross-sectional study. PLoS One.

[7] Warren CE, Abuya T, Askew I, Integra Initiative (2013). Family planning practices and pregnancy intentions among HIV-positive and HIV-negative postpartum women in Swaziland: a cross-sectional survey. BMC Pregnancy Childbirth.

[8] Kikuchi K, Wakasugi N, Poudel KC, Sakisaka K, Jimba M (2011). High rate of unintended pregnancies after knowing of HIV infection among HIV positive women under antiretroviral treatment in Kigali, Rwanda. Biosci Trends.

[9] Loutfy M, Raboud J, Wong J, Yudin M, Diong C, Blitz S (2012). High prevalence of unintended pregnancies in HIV-positive women of reproductive age in Ontario, Canada: a retrospective study. HIV Med.

[10] Calvert C, Ronsmans C (2013). The contribution of HIV to pregnancy-related mortality: a systematic review and meta-analysis. AIDS.

[11] Zaba B, Calvert C, Marston M, Isingo R, Nakiyingi-Miiro J, Lutalo T (2013). Effect of HIV infection on pregnancy-related mortality in sub-Saharan Africa: secondary analyses of pooled community-based data from the network for analysing longitudinal population-based HIV/AIDS data on Africa (ALPHA). Lancet.

[12] Denslow SA, Rositch AF, Fimhaber C, Ting J, Smith JS (2014). Incidence and progression of cervical lesions in women with HIV: a systematic global review. Int J STD AIDS.

[13] Kendall T, Barnighausen T, Fawzi WW, Langer A (2014). Towards comprehensive women's health care in sub-Saharan Africa: addressing intersections between HIV, reproductive and maternal health. J Acquir Immune Defic Syndr.

[14] Abdool Karim Q, Banegura A, Cahn P, Christie C, Dintruff R, Distel M (2011). Asking the right questions: developing evidence-based strategies for treating HIV in women and children. BMC Public Health.

[15] Loutfy MR, Sherr L, Sonnenberg-Schwan U, Walmsley SL, Johnson M, d'Arminio Monforte A (2013). Caring for women living with HIV: gaps in evidence. J Int AIDS Soc.

[16] Gruskin S, Ferguson L, O'Malley J (2007). Ensuring sexual and reproductive health for people living with HIV: an overview of key human rights, policy and health systems issues. Reprod Health Matters.

[17] Ghanotakis E, Peacock D, Wilcher R (2012). The importance of addressing gender inequality in efforts to end vertical transmission of HIV. J Int AIDS Soc.

[18] Orza L, Welbourn A, Bewley S, Tyler Crone E, Vasquez MJ (2014). Building a safe house on firm ground: key findings from a global values and preferences survey regarding the sexual and reproductive health and human rights of women living with HIV.

[19] UN General Assembly (2011). Political declaration on HIV and AIDS: intensifying our efforts to eliminate HIV and AIDS. A/RES/65/277.

[20] Crone ET, Gibbs A, Willan S (2011). From talk to action: review of women, girls and gender equality in national strategic plans for HIV and AIDS in southern and eastern Africa.

[21] Jewkes R, Dunkle K, Nduna M, Shai N (2010). Intimate partner violence, relationship power inequity and incidence of HIV infection in young women in South Africa: a cohort study. Lancet.

[22] Dunkle KL, Decker M (2013). Gender-based violence and HIV: reviewing the evidence for links and causal pathways in the general population and high-risk groups. Am J Reprod Immunol.

[23] World Health Organization (2013). 16 ideas for addressing violence against women in the context of the HIV epidemic: a programming tool.

[24] Kyegombe N, Abramsky T, Devries KM, Starmann E, Michau L, Nakuti J (2014). The impact of SASA!, a comunity mobilization intervention on reported HIV-related risk behaviours and relationship dynamics in Kampala, Uganda. J Int AIDS Soc.

[25] Jewkes R, Nduna M, Levin J, Jama N, Dunkle KL, Puren A (2008). Impact of stepping stones on incidence of HIV and HSV-2 and sexual behaviour in rural South Africa: cluster randomised controlled trial. BMJ.

[26] Pronyk PM, Hargreaves JR, Kim JC, Morison LA, Phetla G, Watts C (2006). Effect of a structural intervention for the prevention of intimate partner violence and HIV in rural South Africa: a cluster randomized trial. Lancet.

[27] Wagman JA, Gray RH, Campbell JC, Thoma M, Ndyanabo A, Ssekasanvu J (2015). Effectiveness of an integrated intimate partner violence and HIV prevention intervention in Rakai, Uganda: analysis of an intervention in an existing cluster randomised cohort. Lancet Glob Health.

[28] Senarath U, Gunawardena NS (2009). Women's autonomy in decision making for health care in South Asia. Asia Pac J Public Health.

[29] Atteraya MS, Kimm H, Song IH (2014). Women's autonomy in negotiating safer sex to prevent HIV: findings from the 20 11 Nepal Demographic and Health Survey. AIDS Educ Prev.

[30] Wellings K, Collumbien M, Slaymaker E, Singh S, Hodges Z, Patel D (2006). Sexual behaviour in context: a global perspective. Lancet.

[31] Sofolahan Y, Airhjhenbuwa CO (2013). Cultural expectations and reproductive desires: experiences of South African women living with HIV/AIDS (WLHA). Health Care Women Int.

[32] Kawale P, Mindry D, Stramotas S, Chiliko P, Phoya A, Henry K (2014). Factors associated with desire for children among HIV-infected women and men: a quantitative and qualitative analysis from Malawi and implications for delivery of safer conception counselling. AIDS Care.

[33] Ketchen B, Armistead L, Cook SL (2009). HIV infection, stressful life events, and intimate relationship power: the moderating role of community resources for black South African women. Women Health.

[34] Hatcher AM, Woollet N, Pallitto CC, Mokoatle K, Stoeckl H, MacPhail C (2014). Bidirectional links between HIV and intimate partner violence in pregnancy: implications for prevention of mother-to-child transmission. J Int AIDS Soc.

[35] Wanvenze RK, Wagner GJ, Tumwesigye NM, Nannyonga M, Wabiwire-Mangen F, Kamya MR (2013). Fertility and contraceptive decision-making and support for HIV infected individuals: client and provider experiences and perceptions at two HIV clinics in Uganda. BMC Public Health.

[36] Nattabi B, Li J, Thompson SC, Orach CG, Earnest J (2009). A systematic review of factors influencing fertility desires and intentions among people living with HIV/AIDS: implications for policy and service delivery. AIDS Behav.

[37] Keogh SC, Urassa M, Roura M, Kumogola Y, Kalongoji S, Kimaro D (2012). The impact of antenatal HIV diagnosis on postpartum childbearing desires in northern Tanzania: a mixed methods study. Reprod Health Matters.

[38] Shannon K, Leiter K, Phaladze N, Hlanze Z, Tsai AC, Heisler M (2012). Gender inequity norms are associated with increased male-perpetrated rape and sexual risks for HIV infection in Botswana and Swaziland. PLoS One.

[39] Fladseth K, Gafos M, Newell ML, McGrath N (2015). The impact of gender norms on condom use among HIV-positive adults in Kwazulu-Natal, South Africa. PLoS One.

[40] Fulu E, Jewkes R, Roselli T, Garcia-Moreno C, UN Multi-country Cross-sectional Study on Men and Violence Research Team (2013). Prevalence of and factors associated with male perpetration of intimate partner violence: findings from the UN Multi-country cross-sectional study on men and violence in Asia and the Pacific. Lancet Glob Health.

[41] World Health Organization (WHO), London School of Hygiene and Tropical Medicine, South African Medical Research Council (2013). Global and regional estimates of violence against women: prevalence and health effects of intimate partner violence and non-partner sexual violence.

[42] Devries KM, Mak JY, Garcia-Moreno C, Petzold M, Child JC, Falder G (2013). The global prevalence of intimate partner violence against women. Science.

[43] Pantalone DW, Rood BA, Morris BW, Simoni JM (2014). A systematic review of the frequency and correlates of partner abuse in HIV-infected women and men who partner with men. J Assoc Nurses AIDS Care.

[44] Obermeyer CM, Baijal P, Pegurri E (2011). Facilitating HIV disclosure across diverse settings: a review. Am J Public Health.

[45] Bott S, Obermeyer C (2013). The social and gender context of HIV disclosure in sub-Saharan Africa: a review of policies and practices. SAHARA J.

[46] Medley A, Garcia-Moreno C, McGill S, Maman S (2004). Rates, barriers and outcomes of HIV serostatus disclosure among women in developing countries: implications for prevention of mother-to-child transmission programmes. Bull World Health Organ.

[47] Gari T, Habte D, Markos E (2010). HIV positive status disclosure among women attending ART clinic at Hawassa University Referral Hospital, South Ethiopia. East Afr J Public Health.

[48] Iliyasu Z, Abubakar IS, Babashani M, Galadanci HS (2011). Domestic violence among women living with HIV/AIDS in Kano, Northern Nigeria. Afr J Reprod Health.

[49] Mugavero M, Ostermann J, Whetten K, Leserman J, Swartz M, Stangl D (2006). Barriers to antiretroviral adherence: the importance of depression, abuse, and other traumatic events. AIDS Patient Care STDS.

[50] Mugavero MJ, Raper JL, Reif S, Mugavero MJ, Raper JL, Reif S (2009). Overload: impact of incident stressful events on antiretroviral medication adherence and virologic failure in a longitudinal, multisite human immunodeficiency virus cohort study. Pyschosom Med.

[51] Schafer KR, Brant J, Gupta S, Thorpe J, Winstead-Derlega C, Pinkerton R (2012). Intimate partner violence: a predictor of worse HIV outcomes and engagement in care. AIDS Patient Care STDS.

[52] Lopez EJ, Jones DL, Villar-Loubet OM, Arheart KL, Weiss SM (2010). Violence, coping, and consistent medication adherence in HIV-positive couples. AIDS Educ Prev.

[53] Trimble DD, Nava A, McFarlane J (2013). Intimate partner violence and antiretroviral adherence among women receiving care in an urban Southeastern Texas HIV clinic. J Assoc Nurses AIDS Care.

[54] Anema A, Vogenthaler N, Frongillo EA, Kadiyala S, Weiser SD (2009). Food insecurity and HIV/AIDS: current knowledge, gaps and research priorities. Curr HIV AIDS Rep.

[55] Weiser SD, Tuller DM, Frongillo EA, Senkungu J, Mukiibi N (2010). Food insecurity as a barrier to sustained antiretroviral therapy adherence in Uganda. PLoS One.

[56] McCoy S, Buzdugan R, Mushavi A, Mahomva A, Cowan FM, Padian NS (2015). Food insecurity is a barrier to prevention of mother-to-child HIV transmission services in Zimbabwe: a cross-sectional study. BMC Public Health.

[57] Lu T, Zwicker L, Kwena Z, Bukusi E, Mwaura-Muiru E, Dworkin SL (2013). Assessing barriers and facilitators of implementing an integrated HIV prevention and property rights program in western Kenya. AIDS Educ Prev.

[58] Swaminathan H, Bhatla N, Chakraborty S (2009). Women's property rights as an AIDS response: emerging perspectives from South Asia.

[59] Swaminathan H, Rugadya M, Walker C (2008). Women's property rights, HIV and AIDS and domestic violence: research findings from two districts in South Africa and Uganda.

[60] Strickland RS (2004). To have and to hold: women's property and inheritance rights in the context of HIV/AIDS in sub-Saharan Africa.

[61] Weiser SD, Leiter K, Bangsberg DR, Butler LM, Percy-deKorte F (2007). Food insufficiency is associated with high-risk sexual behaviour among women in Botswana and Swaziland. PLoS One.

[62] Miller CL, Bangsberg DR, Tuller DM, Senkugu J, Kawuma A, Frongillo EA (2011). Food insecurity and sexual risk in an HIV endemic community in Uganda. AIDS Behav.

[63] Deribe K, Woldemichael K, Njau BJ, Yakob B, Biadgilign S, Amberbir A (2010). Gender differences regarding barriers and motivators of HIV status disclosure among HIV-positive service users. SAHARA J.

[64] Visser MJ, Neufeld S, de Villiers A, Makin JD, Forsyth BW (2008). To tell or not to tell: South African women's disclosure of HIV status during pregnancy. AIDS Care.

[65] Addo AA, Marquis GS, Lartey AA, Perez-Escamilla R, Mazur RE, Harding KB (2011). Food insecurity and perceived stress but not HIV infection are independently associated with lower energy intakes among lactating Ghanain women. Matern Child Nutr.

[66] Levy JM, Webb AL, Sellen DW (2010). “On our own we can't manage”: experiences of infant feeding recommendations among Malawian mothers living with HIV. Int Breastfeed J.

[67] West BS, Hirsch JS, El Sadr W (2013). HIV and H2O: tracing the connections between gender, water and HIV. AIDS Behav.

[68] Malave S, Ramakrishna J, Heylen E, Bharat S, Ekstrand ML (2014). Differences in testing, stigma and perceived consequences of stigmatization among heterosexual men and women living with HIV in Bengaluru, India. AIDS Care.

[69] Geary C, Parker W, Rogers S, Haney E, Njihia C, Haile A (2014). Gender differences in HIV disclosure, stigma, and perceptions of health. AIDS Care.

[70] Visser M (2012). Women, HIV and stigma. Future Virol.

[71] Closson EF, Mimiaga MJ, Sherman SG, Tangmunkongyorakul A, Friedman RK, Limbada M (2015). Intimacy versus isolation: a qualitative study of sexual practices among sexually active HIV-infected patients in HIV care in Brazil, Thailand and Zambia. PLoS One.

[72] Brody LR, Stokes LR, Dale SK, Kelso GA, Cruise RC, Weber KM (2014). Gender roles and mental health in women with and at risk for HIV. Psychol Women Q.

[73] Simbayi LC, Kalichman S, Strebel A, Cloete A, Henda N, Mqeketo A (2007). Internalized stigma, discrimination, and depression among men and women living with HIV/AIDS in Cape Town, South Africa. Soc Sci Med.

[74] MacCarthy S, Rasanathan JJ, Ferguson L, Gruskin S (2012). The pregnancy decisions of HIV-positive women: the state of knowledge and way forward. Reprod Health Matters.

[75] Turan JM, Nyblade L (2013). HIV-related stigma as a barrier to achievment of global PMTCT and maternal health goals: a review of the evidence. AIDS Behav.

[76] Kawale P, Mindry D, Phoya A, Jansen P, Hoffman RM (2015). Provider attitudes about childbearing and knowledge of safer conception at two HIV clinics in Malawi. Reprod Health.

[77] International Planned Parenthood Federation (IPPF) (2011). Piecing it together for women and girls: the gender dimensions of stigma and discrimination.

[78] Kendall T, Albert C (2015). Experiences of coercion to sterilize and forced sterilization among women living with HIV in Latin America. J Int AIDS Soc.

[79] UNDP HIV/AIDS Group (2012). Global commission on HIV and the law: risk, rights and health.

[80] Maman S, Medley A (2004). Gender dimensions of HIV status disclosure to sexual partners: rates, barriers and outcomes. A review paper.

[81] World Health Organization (WHO) (2014). Global update on the health sector response to HIV, 2014.

[82] Skovdal M, Campbell C, Madanhire C, Mupambireyi Z, Nyamukapa C, Gregson S (2011). Masculinity as a barrier to men's use of HIV services in Zimbabwe. Glob Health.

[83] Nattrass N (2008). Gender and access to antiretroviral treatment in South Africa. Fem Econ.

[84] Ahmed S, Kim MH, Abrams EJ (2013). Risks and benefits of lifelong antiretroviral treatment for pregnant and breastfeeding women: a review of the evidence for the Option B+ approach. Curr Opin HIV AIDS.

[85] Colvin CJ, Konopka S, Chalker JC, Jonas E, Albertini J, Amzel A (2014). A systematic review of health system barriers and enablers for antiretroviral therapy (ART) for HIV-infected pregnant and postpartum women. PLoS One.

[86] Kamuyango AA, Hirschhorn LR, Wang W, Jansen P, Hoffman RM (2014). One-year outcomes of women started on antiretroviral therapy during pregnancy before and after the implementation of Option B+ in Malawi: a retrospective chart review. World J AIDS.

[87] Ngarina M, Tarimo EA, Naburi H, Kilewo C, Mwanyika-Sando M, Chalamilla G (2014). Women's preferences regarding infant or maternal antiretroviral prophylaxis for prevention of mother-to-child transmission of HIV during breastfeeding and their views on Option B+ in Dar es Salaam, Tanzania. PLoS One.

[88] Clouse K, Schwartz S, Van Rie A, Bassett J, Yende N, Pettifor A (2014). What they wanted was to give birth, nothing else: barriers to retention in option B+ HIV care among postpartum women in South Africa. J Acquir Immune Defic Syndr.

[89] Webb R, Cullel M (2013). Understanding the perspectives and/or experiences of women living with HIV regarding. Option B+ in Uganda and Malawi.

[90] Carter AJ, Bourgeois S, O'Brien N, Abelsohn K, Tharao W, Greene S (2013). Women-specific HIV/AIDS services: identifying and defining the components of holistic service delivery for women living with HIV/AIDS. J Int AIDS Soc.

# Sub article: Advancing the sexual and reproductive health and human rights of women living with HIV: a review of UN, regional and national human rights norms and standards

**DOI:** 10.7448/IAS.18.6.20280

**Published:** 2015-12-01

**Authors:** Rajat Khosla, Nuna Van Belle, Marleen Temmerman

**Affiliations:** Department of Reproductive Health and Research, World Health Organization, Geneva, Switzerland

**Keywords:** sexual and reproductive health, HIV, women living with HIV, human rights

## Abstract

**Introduction:**

The right to sexual and reproductive health (SRH) is an essential part of the right to health and is dependent upon substantive equality, including freedom from multiple and intersecting forms of discrimination that result in exclusion in both law and practice. Nonetheless, general and specific SRH needs of women living with HIV are often not adequately addressed. For example, services that women living with HIV need may not be available or may have multiple barriers, in particular stigma and discrimination. This study was conducted to review United Nations Human Rights Council, Treaty Monitoring Bodies and Special Rapporteur reports and regional and national mechanisms regarding SRH issues of women living with HIV. The objective is to assess areas of progress, as well as gaps, in relation to health and human rights considerations in the work of these normative bodies on health and human rights.

**Methods:**

The review was done using keywords of international, regional and national jurisprudence on findings covering the 2000 to 2014 period for documents in English; searches for the Inter-American Commission on Human Rights and national judgments were also conducted in Spanish. Jurisprudence of UN Treaty Monitoring Bodies, regional mechanisms and national bodies was considered in this regard.

**Results and discussion:**

In total, 236 findings were identified using the search strategy, and of these 129 were selected for review based on the inclusion criteria. The results highlight that while jurisprudence from international, regional and national bodies reflects consideration of some health and human rights issues related to women living with HIV and SRH, the approach of these bodies has been largely *ad hoc* and lacks a systematic integration of human rights concerns of women living with HIV in relation to SRH. Most findings relate to non-discrimination, accessibility, informed decision-making and accountability. There are critical gaps on normative standards regarding the human rights of women living with HIV in relation to SRH.

**Conclusions:**

A systematic approach to health and human rights considerations related to women living with HIV and SRH by international, regional and national bodies is needed to advance the agenda and ensure that policies and programmes related to SRH systematically take into account the health and human rights of women living with HIV.

## Introduction

Protection of the sexual and reproductive health (SRH) and human rights of women living with HIV/AIDS is fundamental to their dignity, health and wellbeing [1]. However, HIV continues to be a leading cause of death among women of reproductive age worldwide. To address this situation, the global HIV response must fully recognize the significant role that gender inequality and violation of human rights plays in increasing women and girls’ vulnerability to HIV [2].

Everyone has the equal rights concerning their SRH. However, women living with HIV/AIDS require special protection in this regard. HIV infection accelerates the natural history of some reproductive illnesses and increases the severity of others [1]. Moreover, infection with HIV has serious effects on the sexual health and wellbeing of women [1]. Studies demonstrate that women and girls living with HIV have less access to prevention, treatment, care and support [3]. There is a growing realization that protection and promotion of SRH and rights, including through improved and sustained investment in women and girls living with HIV, can help countries move towards universal access to HIV prevention, treatment, care and support services [4].

For decades, organizations and groups of women living with HIV, such as the Salamander Trust, the Athena Network, the Global Network for and by People Living with HIV and the International Community of Women Living with HIV/AIDS, have been at the forefront of development of research and normative standards in relation to the SRH and human rights of women living with HIV. The work of these organizations has not only helped in the galvanization of support for the development of normative standards in this regard, but also in the improvement of prevention of treatment and care for women living with HIV [5].

The right to SRH is an essential part of the right to health and is dependent upon substantive equality, including freedom from multiple and intersecting forms of discrimination that exacerbate exclusion in both law and practice [6]. Multiple reports highlight the fact that general and specific SRH needs of women living with HIV are often not adequately addressed [7–9]. For example, the SRH services that women living with HIV need may not be available or these women may face multiple barriers, in particular stigma and discrimination, in accessing existing services (see Supplementary Table 1) [8,10–13].

This study was conducted to review findings of international, regional and national bodies regarding SRH issues of women living with HIV. This study was conducted with the objective to assess key areas of progress and possible gaps in relation to normative development of human right standards by United Nations, regional and national human rights bodies regarding the SRH of women living with HIV.

## Method

The starting point for this study is the UN Population Fund (UNFPA) and the World Health Organization (WHO) guidelines, *Sexual and reproductive health of women living with HIV/AIDS* (2006) [1]. The recommendations on care, treatment and support for women living with HIV/AIDS and their children were used to define the search strategy for this study.

The study reviewed relevant findings of the UN Human Rights Council, Treaty Monitoring Bodies and Special Rapporteurs (these included reports, concluding observations and general comments) in relation to normative developments regarding the human rights of women living with HIV in the context of SRH. The review was done for findings covering 2000 to 2014 for documents in English; searches were also conducted in Spanish for the Inter-American Commission on Human Rights (IACHR/CIDH) including the site of the Organization of American States (OAS) and national judgments. The period of 2000 to 2014 was selected with the view that this is period in which the UN Committee on Economic, Social and Cultural Rights General Comment No. 14 on the right to health laid down the framework on health and human rights [14].

The review process was divided into three stages.

First, an international normative review was undertaken. This step included reviews of findings of the Human Rights Council, Treaty Monitoring Bodies and Special Rapporteur reports. Four databases were therefore used: the OHCHR Universal Human Rights Index; bayefsky.com; the University of Minnesota Human Rights Library; and the Universal Periodic Review (UPR). The list of search terms and databases used for the purposes of this review are included in Supplementary Annex 1. Findings include results from documents of the Committee against Torture; Committee on the Elimination of Discrimination against Women; Committee on the Rights of the Child; Committee on Economic, Social and Cultural Rights; Committee on Civil and Political Rights; Special Rapporteur on Health; Special Rapporteur on Mental and Physical Health; Special Rapporteur on Violence against Women; and UPR Working Group.

Second, a regional normative review was undertaken. This included reviews of findings from resolutions and decisions of regional human rights bodies. Sites from the IACHR/CIDH, including the site of the OAS; the African Commission of Human and Peoples’ rights (including the site of the African Union); and the European Commission of Human Rights (including the site of the Council of Europe) were reviewed. The list of search terms and databases used for this review are included in Supplementary Annex 2.

Third, a national normative review was undertaken. This step included reviews of data extracted from national judgments. Different databases were consulted, including LexisNexis, the Treatment Action Campaign database, the South African Legal Information Institute database, the Center of Reproductive Health database, the Global Health and Rights database and national databases with official publications of judgments. References to judgments were also found in the United Nations Development Programme (UNDP) *Compendium of Judgments for Judicial Dialogue on HIV, Human Rights and the Law in East and Southern Africa* of October 2013, the UNDP *Compendium of Judgments for Judicial Dialogue on HIV, Human Rights and the Law in Asia and the Pacific* of June 2013 and the UNAIDS *Judging the Epidemic: A Judicial Handbook on HIV, Human Rights and the Law* of May 2013. Subsequently, references were used to locate the original decisions, and data were directly extracted from official publications. Where the judgments could not be found, the data extraction table (Supplementary Table 1) indicates this.

In terms of the inclusion criteria, a decision was made to include not only findings where human rights bodies had *explicitly* made observations on the SRH of women living with HIV, but also those that were *implicitly* dealing with these issues even if not specifically addressing the nine agreed-upon human rights dimensions found in the WHO's *Ensuring Human Rights in the Provision of Contraceptive Information and Services* (2014):

1. Equality and non-discrimination (alternate terms: *reduce discrimination, reduce criminalization, combat negative social and cultural attitudes, stigma, prejudice, [domestic] violence, gender inequality*)
2. Participation (alternate terms: *involvement, advocacy, influence*)
3. Privacy and confidentiality
4. Informed decision-making (alternate terms: [*direct*] *consent, choice, coerced, forced, informed, comprehensible*)
5. Availability (alternate terms: *make available, provide, exist*)
6. Accessibility (alternate terms: *access, receive, affordable, eligible*)
7. Acceptability (alternate terms: *conscientious objection, medical ethics, human rights sensitivity*)
8. Quality of services (alternate terms: *proper medical care, adequacy*)
9. Accountability (alternate terms: *liability, responsibility, calling upon state parties, enforcement, legal measures*) [15].

Findings that dealt with issues related to HIV and SRH without a specific focus on issues related to women living with HIV were excluded. Similarly, findings that looked at SRH issues of women without a specific focus on women living with HIV were also excluded. Further, in order to capture the widest array of relevant observations to be found in the normative work, search terms also included *stigma*, *respect* and *disrespect*, as well as *choice*.

Data on determinants of health were included to a limited extent.

## Results and discussion

The principles that are most discussed by international, regional and national bodies or courts, in the context of SRH of women living with HIV, are non-discrimination (see Box 1), accessibility, informed decision-making and accountability (see Supplementary Table 1 for survey findings; see also Figure 1).

**Figure 1:** Review findings.

**Box 1: Key definitions**

1. The *principle of non-discrimination* obliges states to guarantee that human rights are exercised without discrimination of any kind based on, *inter alia*, colour, sex, language, religion, political or other opinion, national or social origin, property, birth or other status, such as disability, age, marital and family status, sexual orientation and gender identity, health status, place of residence and economic and social situation.
2. The *principle of accessibility* implies that health facilities, goods and services have to be accessible to everyone without discrimination.
3. The *principle of informed decision-making* implies giving each person the choice and opportunity to make autonomous reproductive choices. The principle of autonomy, expressed through free, full and informed decision-making, is a central theme in medical ethics and is embodied in human rights law.
4. The *principle of accountability* implies that generally states’ legal, policy and programmatic frameworks and practices should be in line with international, regional and national human rights standards. The establishment of effective accountability mechanisms is intrinsic to ensuring that the choices of individuals are respected, protected and fulfilled. Effective accountability requires individuals to be aware of their entitlements with regard to SRH and of the mechanisms available to them.

A total of 236 findings were identified based on the search strategy. Based on the inclusion criteria, 129 findings were selected, the full text was reviewed and data were extracted. The results of the review were classified according to the nine human rights principles and arranged on the basis of them being *most cited*, *less cited* or *rarely cited*. The authors manually reviewed the findings to ascertain how these principles had been dealt with and the frequency with which these principles were referred to in the human rights normative developments related to women living with HIV and SRH. For the purposes of this classification, principles cited >10 times were classified as *most cited*, principles cited <10 times as *less cited* and principles cited <5 times as *rarely cited*. In addition, principles that did not feature under the nine developed by the WHO but were frequently cited by the UN and/or regional and national human rights bodies were also noted.

### Most-cited human rights principles in relation to women living with HIV and SRH

#### The principle of non-discrimination

The review of international, regional and national jurisprudence of normative standards found that the most-cited human rights principle in relation to the SRH of women living with HIV is the need to combat discrimination and violence against women living with HIV. The findings from various human rights bodies refer to the need to eliminate discrimination against women, girls and adolescents living with HIV through challenging gender inequality, stereotypes, stigma, prejudice and violence. According to the findings, discrimination toward women living with HIV occurs primarily within families, communities and healthcare facilities.

Furthermore, violence is highlighted in the findings of human rights bodies as a central concern with regard to the SRH of women living with HIV. The findings highlight the need to eliminate violence by addressing gaps in legislation and policy. Violence or fear of violence is identified as a prime barrier to HIV testing and disclosure of a women's seropositive status. Different types of violence (psychological and physical) are mentioned, including sexual violence, prejudicial traditional or customary practices, coercion or abuse, early and forced marriage, fear of conflict with partners, forced vaginal examinations, mandatory testing and involuntary sterilization. The findings emphasize that women living with HIV are more likely to experience violence than men living with HIV [16]. In this context, it is important to highlight the findings on the need to empower women, support their economic independence and protect their fundamental rights and freedoms, including their SRH rights.

Human rights bodies also cite stigma and prejudice as leading obstacles to the enjoyment of SRH by women living with HIV. They impede the access of women living with HIV to justice and severely limit or deny the enjoyment of these women's SRH.

The findings of human rights bodies further identify gender inequalities and stereotypes as a major issue. The vulnerability of women and girls living with HIV/AIDS is a major human rights challenge because of the effect of inequality between the sexes. Mothers are held solely responsible for infecting their children. Women are held responsible for HIV transmission by the very person who infected them, and HIV-positive men sometimes believe that they have the right to maintain the pleasure of unprotected sex [17]. The findings also highlight the relationship between violence and gender stereotypes. The findings emphasize the need to combat discrimination and violence by addressing gaps in legislation and policy, putting programmes into place and implementing initiatives [18].

#### The principle of accessibility

Accessibility of information and services related to SRH remains a challenge for women living with HIV. The findings of human rights bodies indicate issues of discrimination in accessibility by women living with HIV to SRH information and services, in particular in family planning, pregnancy and childcare. Most findings are related to treatment of women in their reproductive years and some to female children; however, some categories of women, such as women without children and older women, are hardly taken into account. Nevertheless, one particular reference stresses the need for equitable access to SRH care throughout the lives of women living with HIV [19] and is one of the few examples whereby human rights bodies have made explicit reference to the importance of access to treatment throughout women's lives.

Findings of human rights bodies also point to the physical inaccessibility of most rural and marginalized women living with HIV to healthcare services, which leads to delays and difficulties in the utilization of adequate information and services. Furthermore, findings highlight that migrant women living with HIV also face social, language, legal and financial barriers and are exposed to the risk of inaccessibility to services when submitted to deportation [20,21].

Economically accessible information and SRH services, such as HIV testing, counselling, contraceptives and antiretroviral (ARV) treatment, are often supported, according to the findings of human rights bodies. However, as with the physical accessibility of services, all these references primarily focus on pregnant women's economic accessibility to services.

#### The principle of informed decision-making

Women living with HIV are often sterilized without their knowledge or consent, and there is a need for education about the effects of sterilization and the alternatives available [22]. In addition, pregnant women living with HIV are often advised or pressured to terminate their pregnancies [8]. The review of findings of human rights bodies highlights the need for these women to be informed about ARV medication during pregnancy and delivery and after birth. Findings also highlight that many women are submitted to mandatory HIV testing and therefore emphasize the need for free and informed consent with regard to all medical procedures [23]. Within this context, a large number of findings relate to the lack of information on prevention of mother-to-child transmission (PMTCT) of HIV.

#### The principle of accountability

Findings of human rights bodies refer to the need to encourage a policy, legal and social environment that promotes human rights for women living with HIV, ensuring the full recognition of their SRH and rights. Findings point towards the need to address existing gaps in HIV-related legislation and policy and further highlight the need to effectively use parliamentary processes. National mechanisms such as commissions, courts, legislation and coordinated strategies must be strengthened to protect, enforce and monitor the human rights of women living with HIV. Implementation and enforcement of protection in law for women living with HIV remains a challenge. The issue of criminalization of HIV transmission to others and, in the case of pregnant women, to the foetus is also emphasized in several human rights bodies’ findings [24,25].

Furthermore, the findings of human rights bodies points towards evidence that women living with HIV face multiple forms of discrimination with regard to access to justice. Findings highlight the need to put reinsertion programmes into place for women living with HIV who are victims of discrimination.

### Less-cited human rights principles in relation to women living with HIV and SRH

The results from the review of findings from human rights bodies reflect a primary focus on issues related to non-discrimination, accessibility, informed decision-making and accountability in international, regional and national jurisprudence related to women living with HIV and SRH. Some additional references are also found for other key health and human rights considerations, in particular availability and privacy and confidentiality.

#### Principle of availability

Within the principle of availability, the human rights bodies’ findings primarily focus on PMTCT [26–28]. A lot of references are made to the availability of sufficient quantity of goods and services and programmes [29]. The availability of goods focuses primarily on ARV treatment for PMTCT. The findings also refer to the availability of sufficient and regular paediatric ARV treatment and the availability of ARVs in prisons and public hospitals. The availability of services is also primarily dealt with in the context of PMTCT. Within this context, the findings underline the need for prevention of unintended pregnancies and for appropriate antenatal, delivery and postpartum care, including counselling on infant feeding options.

Furthermore, the findings underline the importance of the integration of HIV/AIDS services in SRH care and *vice versa*. Most findings relate to the importance of integrating HIV/AIDS issues in SRH programmes [25,30,31].

#### Privacy and confidentiality

Findings of human rights bodies on confidentiality and privacy primarily deal with the disclosure of women's HIV status. There is a lack of confidentiality in health facilities, schools, prisons and courts. Test results are made available to husbands, friends, families and the community at large.

### Least-cited human rights principles in relation to women living with HIV and SRH

The principles of acceptability, quality and participation are least dealt with in international, regional and national human rights jurisprudence related to women living with HIV and SRH. Whereas hardly any references are found regarding principles related to acceptability and quality, there are some references related to participation. Participation is primarily emphasized with respect to women living with HIV, as well as civil society at large, which must be encouraged to participate in the development and implementation of national policies and actions. Religious communities are encouraged to include provisions on premarital HIV counselling and testing in their by-laws.

### Newly cited principles: determinants of health

The analysis of jurisprudence also points to some references to the experience of discrimination faced by women living with HIV in access to housing, education, employment, healthcare and justice [32]. These principles are in addition to the nine health and human rights principles of WHO and are noted here for their relevance to the issue of women living with HIV and SRH.

### Gaps and challenges

These findings clearly illustrate that while international, regional and national bodies have been considering issues related to health and human rights of women living with HIV and SRH, various health and human rights considerations are often not systematically addressed.

The study identified some key limitations in the way that UN human rights mechanisms have dealt with issues related to women living with HIV and SRH. These include the following issues.

#### Ambiguity around the subject of women living with HIV

An overwhelming number of references to mother-to-child transmission or vertical transmission of the virus were found in the review, and although the prevalence of HIV among women is said to be “particularly concerning” in its own right, the focus was on *its potential to transmit the disease through child rearing*. In addition, many items promoting SRH for women living with HIV rely on vague terms. For example, some documents provide that states should “eliminate discrimination against women and persons living with HIV.” This statement leaves its subject unclear. Should member states not discriminate against women *and* persons with HIV (separate categories)? Or rather, should the statement be understood as non-discrimination against “women *and/or* persons living with HIV?” This ambiguity in the way the issues have been dealt with obscures meaning and impact of the findings. One cannot assume that lists of disadvantaged social categories incorporate persons at the intersection.

#### Ambiguity around the subject of sexual and reproductive health

Often, references to the “prevention and future control of HIV” and “human rights guarantees” for women living with HIV are mixed with specific human rights related to women living with HIV and SRH. Despite the fact that SRH *is* a human right, not all persons agree on the extent to which the former falls under the purview of the latter. These issues have therefore been handled with an overall lack of specificity.

This review points out that despite rhetorical attention, there is little jurisprudence and systematic integration of human rights related to women living with HIV in the context of SRH. As this review of the jurisprudence shows, there are clear gaps and areas of concern that have not yet been sufficiently addressed.

A number of critical human rights issues have not been well addressed, for example the economic independence and financial security of women living with HIV and its influence on their ability to exercise their sexual and reproductive rights [8]. The Global Commission on HIV and the Law noted that when women lack the protection of laws that recognize equal rights to property, they are more likely to be rendered economically dependent on, and susceptible to, control by their spouses in all domains, including their sexual lives [8].

Furthermore, while issues such as criminalization of SRH services are often dealt with by international, regional and national human rights bodies [33], a systematic analysis is often missing of issues related to misinformation, intimidation tactics and barriers faced by women living with HIV in access to SRH information and services [22]. Within the human rights jurisprudence, there are also persistent gaps in relation to dealing with specific SRH issues, such as unwanted pregnancy, cervical cancer screening and management for women living with HIV and safe abortion services [9]. The review also points towards gaps in relation to normative standards pertaining to fertility issues of women living with HIV generally, specifically in relation to the desire to have children [34], use of SRH services and advice from providers [35]. Overall issues related to training and preparedness of healthcare providers to provide services to women living with HIV are often inadequately dealt with in human rights jurisprudence. Evidence points towards the critical importance of these interventions [36].

## Conclusions

The last 20 years have seen improvements in SRH and human rights in many countries. This advance has been supported by awareness raised by women's health advocates, increasingly by youth groups, and also by organizations of health professionals [37]. In the HIV/AIDS area, the involvement of organizations of people living with HIV/AIDS is crucial to improve prevention and care. The advocacy done by women living with HIV has helped both in the normative development of standards related to women living with HIV and in the improvement of treatment and care at the country level [38]. However, after victories during the 1990s, whereby women's rights groups made strides by combatting opposition from social and political conservatives, recent years have seen the backsliding of gains made [37].

The right to SRH is an essential part of the right to health and is rooted in numerous international human rights instruments. Despite the development in international standards and jurisprudence, the full enjoyment of the right to SRH remains a distant goal for millions of people throughout the world. This analysis of key human rights principles shows that issues related to the human rights of women living with HIV regarding SRH have not been comprehensively dealt with by the UN or other human rights mechanisms. This leaves critical gaps in normative developments in this area, which often result in *ad hoc* integration of these issues into health policies and programming.

At the national level, governments have not dealt with many human rights principles and outcomes as part of their legal and policy response to the human rights of women living with HIV. For example, discrimination, stigma and prejudice against women living with HIV occur primarily within families, communities and healthcare facilities; however, these issues are not appropriately dealt with at the national level. National legislation rarely deals with issues related to availability, privacy and confidentiality, acceptability, quality of services and meaningful participation by the community of women living with HIV. Resulting policies lack human rights guarantees for women living with HIV. There is therefore a clear need for strengthening global, regional and national standard setting for this underserved population. Within the findings of different human rights bodies at the global, regional and national levels, it was found that the language used for articulation of recommendations and standards is often pejorative and stereotypical and does not take into account the health and human rights of women living with HIV.

Further work is also needed to strengthen normative standards at the country level and enhance accountability for the violations of human rights of women living with HIV. Clear normative guidance is needed at the global, regional and national levels to address the SRH and human rights needs of women living with HIV. This work should build on the work of organizations and groups of women living with HIV. Furthermore, regular monitoring of implementation of the recommendations by the UN Human Rights Council through its UPR Working Group and Special Procedure mechanism can help enhance accountability for the human rights of women living with HIV.

A promising vision has been created by the growing youth movement for SRH and rights and the potential for opening up larger alliances around sexual and bodily rights with HIV/AIDS activists, sex workers, people living with HIV and AIDS and human rights organizations [38]. Together these alliances can lead to a meaningful change in the lives of this vulnerable group [39].

## Supplementary Material

Advancing the sexual and reproductive health and human rights of women living with HIV: a review of UN, regional and national human rights norms and standards Click here for additional data file.

Advancing the sexual and reproductive health and human rights of women living with HIV: a review of UN, regional and national human rights norms and standards Click here for additional data file.

## References

[1] WHO (2006). Sexual and reproductive health of women living with HIV.

[2] UNAIDS (2014). The GAP report.

[3] Shapiro K, Ray S (2007). Sexual health for people living with HIV. Reprod Health Matters.

[4] UNAIDS (2011). The right of women and girls living with HIV to sexual and reproductive health takes centre stage at the CSW.

[5] UN Population Division, International Conference on Population and Development, Cairo (1994).

[6] http://www.salamandertrust.net.

[7] UNFPA (2014). Framework of actions for the follow-up to the programme of action of the international conference on population and development.

[8] El Feki S, Avafia T, Fidalgo TM, Divan V, Chauvel C, Dhaliwal M (2014). The Global Commission on HIV and the Law: recommendations for legal reform to promote sexual and reproductive health and rights. Reprod Health Matters.

[9] de Bruyn M (2012). HIV, unwanted pregnancy and abortion – where is the human rights approach?. Reprod Health Matters.

[10] Campero L, Kendall T, Caballero M, Mena AL, Herrera C (2010). Exercising sexual and reproductive rights: a qualitative study of heterosexual people with HIV in Mexico. Salud Publica Mex.

[11] Bell E, Mthembu P, O'Sullivan S, Moody K, International Community of Women Living with HIV/AIDS, Global Network of People Living with HIV/AIDS (2007). Sexual and reproductive health services and HIV testing: perspectives and experiences of women and men living with HIV and AIDS. Reprod Health Matters.

[12] Leiter K, Suwanvanichkij V, Tamm I, Iacopino V, Beyrer C (2006). Human rights abuses and vulnerability to HIV/AIDS: the experiences of Burmese women in Thailand. Health Hum Rights.

[13] de Bruyn M (2004). Living with HIV: challenges in reproductive health care in South Africa. Afr J Reprod Health.

[14] (2000). UN, Committee on Economic, Social and Cultural Rights: general Comment 14, E/C.12/2000/4.

[15] WHO (2014). Ensuring human rights in the provision of contraceptive information and services.

[16] (2007). COE, Parliamentary Assembly, Recommendation 1785.

[17] UN Special Rapporteur on Violence Against Women, Report to the Commission on Human Rights, E/CN.4. (2005) Para 68.

[18] (2007). CIM (AOS), El Salvador, OEA/Ser.L/II.2.33; CIM/doc.49/06 rev. 4.

[19] (2007). OAS, Inter-American Commission of Women, CIM/DEC.4/07 (VII-E/07).

[20] (2014). CoE, Committee on Migration, Refugees and Displaced Persons, Doc. 13391.

[21] (2014). CoE, Parliamentary Assembly, Resolution 1997.

[22] Kendall T, Albert C (2015). Experiences of coercion to sterilize and forced sterilization among women living with HIV in Latin America. J Int AIDS Soc.

[23] (2013). ACHPR, Gambia, 54th Ordinary Session, Resolution 260.

[24] UN Special Rapporteur on Right to Health, A/66/150 para 40–41.

[25] UN Special Rapporteur on Violence against Women, E/CN.4/2005/72 para 84.

[26] UN Special Rapporteur on Right to Health, A/58/427 para 60–61.

[27] CRC, C/8/Add.23 (1995) para 88.

[28] High Court of Delphi at New Delphi, AZ v Medical superintendent SMT.

[29] (2010). Tribunal Constitucional de Bolivia.

[30] (2006). AU, Mozambique, Sp/MIN/CAMH/5(I).

[31] (2006). AU, Ethiopia, Sp/Assembly/ATM/2 (I) Rev.3.

[32] Industrial Court of Botswana, Diau v. Botswana Building Society, IC NO 50 OF 2003; 2003 (2) B.L.R. 409 (BwIC) [2003]; High Court of Lagos State, Georgina Ahamefule v. Imperial Medical Centre (Suit No. ID/16272000), [2012]; High Court of Calcutta, Payel Sarkar v. Central Board of Secondary Education and Ors., AIR 2010 Calcutta 74 [2010].

[33] Fried ST, Kelly B (2011). Gender, race+geography=jeopardy: marginalized women, human rights and HIV in the United States. Womens Health Issues.

[34] Mantell JE, Smit JA, Stein ZA (2009). The right to choose parenthood among HIV-infected women and men. J Public Health Policy.

[35] Messersmith LJ, Semrau K, Anh TL, Trang NN, Hoa DM, Eifler K (2012). Women living with HIV in Vietnam: desire for children, use of sexual and reproductive health services, and advice from providers. Reprod Health Matters.

[36] Erhabor O, Akani CI, Eyindah CE (2012). Reproductive health options among HIV-infected persons in the low-income Niger Delta of Nigeria. HIV AIDS (Auckl).

[37] Germain A, Liljestrand J (2009). Women's groups and professional organizations in advocacy for sexual and reproductive health and rights. Int J Gynaecol Obstet.

[38] Corrêa S, Germain A, Petchesky RP (2005). Thinking beyond ICPD + 10: where should our movement be going?. Reprod Health Matters.

[39] Salamander Trust (2015). Building a safe house on firm group.

# Sub article: Human rights and the sexual and reproductive health of women living with HIV – a literature review

**DOI:** 10.7448/IAS.18.6.20290

**Published:** 2015-12-01

**Authors:** Shubha Kumar, Sofia Gruskin, Rajat Khosla, Manjulaa Narasimhan

**Affiliations:** 1 Department of Preventive Medicine, Institute for Global Health, University of Southern California, Los Angeles, CA, USA; 2 Program on Global Health & Human Rights, Institute for Global Health, University of Southern California, Los Angeles, CA, USA; 3 Department of Reproductive Health & Research, World Health Organization, Geneva, Switzerland

**Keywords:** human rights, sexual health, reproductive health, women living with HIV, healthcare

## Abstract

**Introduction:**

Even as the number of women living with HIV around the globe continues to grow, realization of their sexual and reproductive health and human rights remains compromised. The objective of this study was to review the current state of knowledge on the sexual and reproductive health and human rights of women living with HIV to assess evidence and gaps.

**Methods:**

Relevant databases were searched for peer-reviewed and grey literature. Search terms included a combination of MeSH terms and keywords representing women, HIV/AIDS, ART, human rights, sexual and reproductive health. We included both qualitative and quantitative literature published in English, French, or Spanish between July 2011 and December 2014.

**Results and discussion:**

The search yielded 2228 peer-reviewed articles, of which 40 met the inclusion criteria in the final review. The grey literature search yielded 2186 documents of which seven met the inclusion criteria in the final review. Of the articles and documents reviewed, not a single peer-reviewed article described the explicit implementation of rights in programming, and only two documents from the grey literature did so. With one possible exception, no articles or documents were found which addressed rights comprehensively, or addressed the majority of relevant rights (i.e. equality; non-discrimination; participation; privacy and confidentiality; informed decision making; availability, accessibility, acceptability and quality (3AQ) of services individually or in their totality; and accountability). Additional findings indicate that the language of rights is used most often to describe the apparent neglect or violation of human rights and what does exist only addresses a few rights in the context of a few areas within sexual and reproductive health.

**Conclusions:**

Findings from this review suggest the need to better integrate rights into interventions, particularly with attention to provider training, service delivery, raising awareness and capacity building among the community of women living with HIV. Further research is urgently needed to support the sexual and reproductive health and rights of women living with HIV, to identify what works and to inform future programming and policies to improve care, treatment and support for women living with HIV.

## Introduction

Realization of the sexual and reproductive health (SRH) of women living with HIV remains a key challenge, in part due to a lack of integration of human rights in health programming and policies affecting them, and often because of the outright neglect and violation of their human rights in many aspects of their lives. Reproductive health can be understood as a state of complete physical, mental and social well-being, and not merely the absence of disease or infirmity, in all matters relating to the reproductive system and to its functions and processes. Sexual health is concerned with the enhancement of life and personal relations, and not merely with counselling and care related to reproduction and sexually transmitted diseases.

Universal access to quality SRH information and services is essential to achieving the highest attainable standard for health, including SRH, and requires respecting, protecting and fulfilling the human rights of all individuals, including women living with HIV. In 2006, the World Health Organization (WHO) released the *Guideline on Sexual and Reproductive Health of Women Living with HIV/AIDS: Guidelines on Care, Treatment and Support for Women Living with HIV/AIDS and Their Children* [1]. In 2014, WHO advanced a framework which focused attention on specific human rights in the provision of contraceptive information and services [2]. Given the need to consider relevant rights with explicit attention to women living with HIV, along with changes in both the bio-medical and political aspects of the HIV response, a key step in support of advancing this process is a review of the literature to identify the existing evidence and gaps in interventions to address/promote human rights in the broader context of SRH programmes and policies for women living with HIV.

This literature review builds on a study by MacCarthy *et al*. [3] that examined the pregnancy-related needs, rights and decisions of women living with HIV and reported several issues: contraceptive options for pregnancy prevention for women living with HIV are insufficient – condoms are not always available or acceptable, and other options are limited by affordability, availability or efficacy; coerced sterilizations of women living with HIV is widely reported; information gaps persist in relation to effectiveness, safety and best practices regarding assisted reproductive technologies; attention to neonatal outcomes generally outweighs attention to the health of women before, during and after pregnancy; access to safe abortion and post-abortion care services are often curtailed; there is inadequate attention to HIV-positive sex workers, injecting drug users and adolescents; and the many challenges women living with HIV encounter in their interactions with SRH services contribute to their pregnancy decisions. Authors suggested it was critical that women living with HIV be more involved in the design and implementation of research, policies and programmes related to their pregnancy-related needs and rights. Authors noted several human rights issues in their analysis, including the availability, accessibility, affordability, acceptability and quality of services, participation, as well as the neglect of human rights more generally as requiring attention for the fulfilment of the SRH and rights of women living with HIV [3]. A review of United Nations and regional human rights norms and standards relevant to the SRH and human rights of women living with HIV are found elsewhere in this volume [4]. The objective of the present study is to review the current state of knowledge on the SRH and human rights of women living with HIV in order to assess evidence and gaps, and suggest areas requiring further study and action.

## Methods

With a focus on current knowledge, we conducted a targeted search of peer-reviewed and grey literature published in English, French or Spanish between July 1, 2011, and December 31, 2014. This review, which brings explicit attention to many aspects of the SRH and rights of women living with HIV, builds on the findings and conclusions of the literature review by MacCarthy *et al*. [3] summarizing the literature through June 2011 with an explicit focus on the pregnancy intentions of women living with HIV [3]. This review looks at the literature since that time and casts a wider net to include the topics included in the WHO guideline (2006) such as sexual health; family planning; antenatal, intrapartum, postpartum and newborn care; eliminating unsafe abortion; sexually transmitted infections (STIs), reproductive tract infections (RTIs) and cervical cancer, as well as violence and ageing. Databases including PubMed, Web of Science, Social Science Research Network (SSRN), Global Health, Public Affairs Information Service (PAIS) and International Bibliography of Social Sciences were searched for peer-reviewed literature using a combination of MeSH terms and keywords representing women, HIV/AIDS, ART, human rights and the above named areas within SRH. Databases including ProQuest Dissertations and Theses, New York Academy of Medicine Grey Literature Report, WHO Global Health Library, Scopus, Popline and PAIS were searched for grey literature using combinations of the keywords above. With respect to human rights, the decision was made to cast a wide net in order to include not only articles and documents where researchers had explicitly determined that human rights were part of their actions, but also those that were implicitly dealing with human rights using the nine agreed upon human rights dimensions found in the WHO Framework for Ensuring Human Rights in the Provision of Contraceptive Information and Services as the basis of this work [2] (i.e. equality; non-discrimination; participation; privacy and confidentiality; informed decision making; availability, accessibility, acceptability and quality (3AQ) of services individually or in their totality; and accountability) [2]. Thus, in order to capture the widest array of rights concepts to be found in the literature, search terms included also equity, stigma, respect and disrespect, as well as reproductive choice [2] (more details on the exact search strategy can be found in the appendix).

The peer-reviewed search results were imported into EndNote organized by SRH topic. By contrast, due to limitations in the search capacity of the grey databases, grey literature search results were imported into EndNote in relation to SRH more broadly. An initial review of each abstract or summary was completed followed by a full-text review of all articles and documents appearing to meet the inclusion criteria. Articles or documents were included in the review if they had as their primary focus the SRH and rights of women living with HIV or women receiving ART, including (but not limited to) SRH education and promotion, family planning, pregnancy, childbirth, eliminating unsafe abortion, STIs, violence and ageing. Finally, in order to broaden the pool of articles and documents reviewed, it was agreed that they need not focus only on women to be included, but that the extent to which attention was given to women in each specific case would be noted. Upon full-text review, any articles or documents which were then determined not to meet the inclusion criteria were removed.

Through this process, a final group of articles and documents meeting the inclusion criteria was identified. Of note, as there is a plethora of articles and documents that address stigma, but do not take discrimination or any other human rights standard into account in doing so, stigma-related articles and documents were included only if they explicitly made linkages to discrimination or other human rights. Relevant data (i.e. author(s), year, title, article/document type, abstract, population focus, geographic focus, human rights dimension(s) and SRH topic(s)) from all articles and documents identified for inclusion were abstracted and entered into an Excel table. Finally, a qualitative content analysis was conducted of the final pool of articles and documents to determine how exactly human rights had been addressed in each, including the types and frequency of mention of human rights and the context in which they were raised (i.e. human rights-based programming, human rights violations, etc.). Based on analysis and discussion of individual articles and documents within each of the SRH topics, broader themes that emerged within the topic were identified and are summarized in each topical sub-section of the results.

## Results and discussion

### Results of the search

The search of peer-reviewed literature yielded 2228 unique articles. All abstracts were reviewed and 42 were selected for full-text review, 40 of which met the inclusion criteria in the final review (Figure 1). Of the 40 publications, 32 represented original empirical studies (80% of findings) including 5 quantitative research studies, 14 qualitative research studies, 6 mixed methods studies and 7 policy analyses, while 8 (20% of findings) publications were either literature reviews, conceptual pieces, or viewpoints. Twenty-seven (68%) of these publications focused or drew on data from low- and middle-income countries (LMIC), while eight (20%) were global in scope. Thirty-nine (98%) of them were written in English, one in Spanish (2%) and none in French.

**Figure 1:** Results of the search strategy (peer-reviewed and grey). *Of the 42 full-text peer-reviewed articles assessed for inclusion, one was removed based on full-text review and one was removed due to inaccessibility of the full-text article.

The search for grey literature yielded 2186 documents of which seven met the inclusion criteria in the final review. Of the seven documents, three were programme reports (43%), one was a programming guide (14%) and three were general documents related to the subject (43%). Three (43%) of these documents focused or drew on data from LMIC while four (57%) were global in scope. All seven (100%) of them were written in English.

Consistent with the topics included in the WHO guideline (2006), and the additional emphasis on violence and ageing, retrieved citations were organized by SRH topics pertinent to women living with HIV and women receiving ART. Upon review of the literature, it became evident the distinction between women living with HIV and those receiving ART was no longer pronounced in the literature. Results were therefore combined. In addition, the search brought to light some articles and documents which could not be easily placed within the identified topical areas but were relevant to the task at hand. These were ultimately placed in two new groupings: one on integrated models of care and the other on structural, societal and contextual factors impacting the SRH and rights of women living with HIV.

Table 1 indicates the final number of articles and documents that met the inclusion criteria according to the primary topic they addressed from the total citations retrieved.

**Table 1:** Number of articles and documents that met inclusion criteria by primary SRH topical area

Topics	Peer-reviewed articles: women living with HIV and/or women receiving ART (combined)	Grey documents: women living with HIV and/or women receiving ART (combined)
Promoting sexual health	3	1
Providing high-quality services for family planning	12	1
Improving antenatal, intrapartum, postpartum and newborn care	6	1
Eliminating unsafe abortion	5	0
Combating sexually transmitted infections, reproductive tract infections and cervical cancer	1	0
Reducing violence	1	2
Promoting healthy ageing	0	1
Integrated models of care	4	1
Structural, societal and contextual factors impacting sexual and reproductive health and rights	8	0
Total	40	7^a^
Total (peer-reviewed or grey)	47	

a Salamander Trust [8] is counted only once in the “promoting healthy ageing” category in the above table even as the document equally focused on other categories (i.e. promoting sexual health, providing high-quality services for family planning; and reducing violence) but is referred to in the text within each of those categories.

As the primary focus of this review was to determine how human rights concerns are addressed in the literature, an effort was made also to group articles so as to highlight which rights principles are most often addressed and with respect to which SRH topics. Table 2 presents all the peer-reviewed articles included in our review organized by author(s), year of publication, title, and the human right(s) and/or right(s)-related issue(s) addressed in each (i.e.(general) human rights, “human rights principles,” human rights violations, reproductive rights, reproductive and sexual rights, right to health, women's rights and rights-based approach). Table 3 presents all the grey documents in our review organized similarly by author(s), year of publication, title, and the human right(s) and/or right(s)-related issue(s) addressed in each.

**Table 2:** Human rights aspects addressed by peer-reviewed articles included in the review

Author(s)	Year	Title	Human right(s) and/or rights-related issue(s) addressed
C. Barroso & S. Sippel	2011	Sexual and reproductive health and rights: integration as a holistic and rights-based response to HIV/AIDS	Discrimination; equality; human rights violations; reproductive and sexual rights; rights-based approach; quality; women's rights
C. Beyrer, S. Baral, D. Kerrigan, N. El-Bassel, L.-G. Bekker and D. D. Celentano	2011	Expanding the space: inclusion of most-at-risk populations in HIV prevention, treatment, and care services	Accessibility; criminalization; discrimination; human rights principles; participation
C. H. Logie, L. James, W. Tharao and M. R. Loutfy	2011	HIV, gender, race, sexual orientation, and sex work: a qualitative study of intersectional stigma experienced by HIV-positive women in Ontario, Canada	Accessibility; discrimination
H. MacGregor and E. Mills	2011	Framing rights and responsibilities: accounts of women with a history of AIDS activism	Accessibility; gender inequality; privacy and confidentiality; reproductive and sexual rights; right to health
E. Mykhalovskiy	2011	The problem of “significant risk”: exploring the public health impact of criminalizing HIV non-disclosure	Criminalization; privacy and confidentiality
P. Orner, M. d. Bruyn and D. Cooper	2011	“It hurts, but I don't have a choice, I'm not working and I'm sick”: decisions and experiences regarding abortion of women living with HIV in Cape Town, South Africa	Discrimination; laws/policies; 3AQ
P. J. Orner, M. de Bruyn, R. M. Barbosa, H. Boonstra, J. Gatsi-Mallet and D. D. Cooper	2011	Access to safe abortion: building choices for women living with HIV and AIDS	Accessibility; laws/policies; reproductive and sexual rights
G. Anderson, G. Caswell, O. Edwards, A. Hsieh, B. Hull, C. Mallouris, … C. Nöstlinger	2012	Community voices: barriers and opportunities for programmes to successfully prevent vertical transmission of HIV identified through consultations among people living with HIV	Accessibility; human rights violations; participation; privacy and confidentiality; quality; reproductive and sexual rights
R. M. Barbosa, A. A. Pinho, N. S. Santos and W. V. Villela	2012	Exploring the relationship between induced abortion and HIV infection in Brazil	Accessibility; laws/policies; quality; reproductive and sexual rights
M. de Bruyn	2012	HIV, unwanted pregnancy and abortion – where is the human rights approach?	Accessibility; discrimination; human rights; laws/policies; reproductive rights; women's rights
O. Erhabor, C.I. Akani, & C.E. Eyindah	2012	Reproductive health options among HIV-infected persons in the low-income Niger Delta of Nigeria	Discrimination; laws/policies; reproductive and sexual rights; 3AQ
Z. Essack and A. Strode	2012	I feel like half a woman all the time: the impacts of coerced and forced sterilisations on HIV-positive women in South Africa	Discrimination; human rights violations; informed consent; reproductive and sexual rights
S. Fried, B. Harrison, K. Starcevich, C. Whitaker and T. O'Konek	2012	Integrating interventions on maternal mortality and morbidity and HIV: a human rights-based framework and approach	Accountability; discrimination; gender inequality; human rights violations; participation; right to health; 3AQ
E. Ghanotakis, D. Peacock and R. Wilcher	2012	The importance of addressing gender inequality in efforts to end vertical transmission of HIV	Accessibility; gender inequality
A. Gibbs, E. T. Crone, S. Willan and J. Mannell	2012	The inclusion of women, girls and gender equality in National Strategic Plans for HIV and AIDS in southern and eastern Africa	Accessibility; accountability; gender inequality; laws/policies; reproductive and sexual rights
A. Gibbs, M. Mushinga, E. T. Crone, S. Willan and J. Mannell	2012	How do national strategic plans for HIV and AIDS in southern and eastern Africa address gender-based violence? A women's rights perspective	Human rights violations; laws/policies; women's rights
C. H. Logie, L. James, W. Tharao and M. R. Loutfy	2012	We don't exist: a qualitative study of marginalization experienced by HIV-positive lesbian, bisexual, queer and transgender women in Toronto, Canada	Accessibility; discrimination; participation; quality
S. MacCarthy, J. J. K. Rasanathan, L. Ferguson and S. Gruskin	2012	The pregnancy decisions of HIV-positive women: the state of knowledge and way forward	Participation; reproductive and sexual rights; 3AQ
L. J. Messersmith, K. Semrau, T.L. Anh, N.N. Trang, D.M. Hoa, K. Eifler, & L. Sabin	2012	Women living with HIV in Vietnam: desire for children, use of sexual and reproductive health services, and advice from providers	Accessibility; discrimination; human rights violations; reproductive and sexual rights
G. Paz-Bailey, V. I. Fernandez, S. Morales Miranda, J. O. Jacobson, S. Mendoza, M. A. Paredes, D. C. Danaval, D. Mabey and E. Monterroso	2012	Unsafe sexual behaviours among HIV-positive men and women in Honduras: the role of discrimination, condom access, and gender	Accessibility; discrimination; gender inequality
M. M. Schaan, M. Taylor, J. Puvimanasinghe, L. Busang, K. Keapoletswe and R. Marlink	2012	Sexual and reproductive health needs of HIV-positive women in Botswana – a study of health care worker's views	Discrimination; 3AQ
J. A. Smit, K. Church, C. Milford, A. D. Harrison and M. E. Beksinska	2012	Key informant perspectives on policy- and service-level challenges and opportunities for delivering integrated sexual and reproductive health and HIV care in South Africa	Accessibility; laws/policies
A. Strode, S. Mthembu and Z. Essack	2012	She made up a choice for me: 22 HIV-positive women's experiences of involuntary sterilization in two South African provinces	Discrimination; informed consent; reproductive and sexual rights
C. J. Badul and A. Strode	2013	LM and Others v Government of the Republic of Namibia: the first sub-Saharan African case dealing with coerced sterilisations of HIV-positive women – Quo vadis?	Discrimination; human rights violations; informed consent
S. Bott and C. M. Obermeyer	2013	The social and gender context of HIV disclosure in sub-Saharan Africa: a review of policies and practices	Criminalization; human rights; laws/policies; privacy and confidentiality
B. Chimphamba Gombachika, E. Chirwa, A. Malata, J. Sundby and H. Fjeld	2013	Reproductive decisions of couples living with HIV in Malawi: what can we learn for future policy and research studies?	Accessibility; availability; laws/policies
K. Church, A. Wringe, P. Fakudze, J. Kikuvi, D. Simelane and S. H. Mayhew	2013	Are integrated HIV services less stigmatizing than stand-alone models of care? A comparative case study from Swaziland	Discrimination; 3AQ
G. G. Eamer and G. E. Randall	2013	Barriers to implementing WHO's exclusive breastfeeding policy for women living with HIV in sub-Saharan Africa: an exploration of ideas, interests and institutions	Accessibility; availability; laws/policies; participation
T. Kendall	2013	Falling short of universal access to reproductive health: unintended pregnancy and contraceptive use among Mexican women with HIV	Accessibility; discrimination; reproductive rights
E. J. King, S. Maman, J. M. Bowling, K. E. Moracco and V. Dudina	2013	The influence of stigma and discrimination on female sex workers’ access to HIV services in St. Petersburg, Russia	Accessibility; discrimination; quality
A. K. Laar	2013	Reproductive rights and options available to women infected with HIV in Ghana: perspectives of service providers from three Ghanaian health facilities	Quality; reproductive rights
M. Loutfy, U. Sonnenberg-Schwan, S. Margolese, & L. Sherr	2013	A review of reproductive health research, guidelines and related gaps for women living with HIV	Accessibility; discrimination; reproductive rights
M. Malta and C. Beyrer	2013	The HIV epidemic and human rights violations in Brazil	Discrimination; laws/policies; rights-based approach
K. Clouse, S. Schwartz, A. Van Rie, J. Bassett, N. Yende and A. Pettifor	2014	What they wanted was to give birth; nothing else: barriers to retention in option B plus HIV care among postpartum women in South Africa	Accessibility; discrimination; laws/policies
S. MacCarthy, J. J. K. Rasanathan, A. Crawford-Roberts, I. Dourado and S. Gruskin	2014	Contemplating abortion: HIV-positive women's decision to terminate pregnancy	Laws/policies; reproductive and sexual rights; 3AQ
P. Madhivanan, K. Krupp, V. Kulkarni, S. Kulkarni, N. Vaidya, R. Shaheen, … C. Fisher	2014	HIV testing among pregnant women living with HIV in India: are private healthcare providers routinely violating women's human rights?	Discrimination; human rights violations; informed consent; privacy and confidentiality; rights-based approach; right to health; quality
S. Malavé, J. Ramakrishna, E. Heylen, S. Bharat and M. L. Ekstrand	2014	Differences in testing, stigma, and perceived consequences of stigmatization among heterosexual men and women living with HIV in Bengaluru, India	Gender inequality
E. Marsicano, R. Dray-Spira, F. Lert, C. Aubriere, B. Spire and C. Hamelin	2014	Multiple discriminations experienced by people living with HIV in France: results from the ANRS-Vespa2 study	Discrimination
S. A. Spangler, M. Onono, E. A. Bukusi, C. R. Cohen, & J. M. Turan	2014	HIV-positive status disclosure and use of essential PMTCT and maternal health services in rural Kenya	Women's rights
M. G. van Dijk, K. S. Wilson, M. Silva, X. Contreras, H. D. Fukuda and S. G. Garcia	2014	Health care experiences of HIV-infected women with fertility desires in Mexico: a qualitative study	Discrimination; quality; reproductive rights

**Table 3:** Human rights aspects addressed by grey documents included in the review

Author(s)	Year	Title	Human right(s) and/or rights-related issue(s) addressed
Jain, S., Greene, M., Douglas, Z., Betron, M., & Fritz, K.	2011	Addressing HIV and gender from the ground up. Maanisha Community Focused Initiative to Control HIV: a program to build the capacity of civil society organizations in Kenya	Laws/policies; participation; women's rights
Khan, A.	2011	Gender-based violence and HIV: a program guide for integrating gender-based violence prevention and response in PEPFAR programs	Accessibility; discrimination; gender inequality; human rights; human rights violations; informed consent; participation; privacy and confidentiality; quality; women's rights
Kundu, N. K.	2011	SANGRAM's Collectives. Engaging communities in India to demand their rights	Discrimination; gender inequality; human rights; participation; rights-based approach
Open Society Institute	2011	Against her will: forced and coerced sterilization of women worldwide	Accessibility; accountability; discrimination; human rights violations; informed consent
Orza, L.	2011	Community innovation: achieving sexual and reproductive health and rights for women and girls through the HIV response	Accessibility; discrimination; gender inequality; human rights; laws/policies; participation; reproductive and sexual rights; rights-based approach; women's rights
Amnesty International	2014	Struggle for maternal health: barriers to antenatal care in South Africa	Discrimination; human rights violations; informed consent; privacy and confidentiality; right to health; 3AQ
Salamander Trust	2014	Building a safe house on firm ground: key findings from a global values and preferences survey regarding the sexual and reproductive health and human rights of women living with HIV	Discrimination; gender inequality; human rights; human rights principles; human rights violations; laws/policies; participation; reproductive and sexual rights; 3AQ

Findings are presented in the following sections mirroring the organization of the WHO guideline (2006), followed by the additional topics of violence; ageing; integrated models of care; and structural, societal and contextual factors. Within each category, findings are separated by those which emerged from the peer-reviewed literature, and those which emerged from the grey literature.

### Promoting sexual health

Of the 1469 citations that were retrieved from the peer-reviewed literature search in relation to promoting the sexual health of women living with HIV (as consistent with the WHO guideline this included SRH education, availability of information, access to family planning counselling, etc.), only three met the criteria for inclusion in this review. All three addressed disclosure of HIV status in one way or another. Taken together, several themes emerge: 1) there is a dearth of peer-reviewed literature on the promotion of sexual health for women living with HIV which includes attention to their rights, and what does exist is entirely within the context of disclosure; 2) research is needed as to how best to support women living with HIV in HIV disclosure not only in the immediate but throughout their sexual lives; and 3) discrimination by health providers based on HIV status for women who remain sexually active remains a persistent problem [5–7].

The search of the grey literature yielded a report describing a programme in India, SANGRAM Plus, which states that it deliberately implements rights-based principles in their approaches to enable collective transformation to help marginalized populations, including female sex workers living with HIV, to support one another, including increasing awareness of free health services available to people living with HIV and mechanisms to address violations. A second report from the grey literature presents findings from a global survey on SRH and human rights of women living with HIV. It represents the values and preferences of women living with HIV in a manner intended to be analogous to the form of building a safe house with foundations, walls, and a roof. Among the findings, it identifies challenges faced in a fulfilling and pleasurable sex life associated with fears of the consequences of disclosure, and difficulties with condom availability and negotiation. Taken together, these findings echo the need to support women living with HIV throughout their sexual lives and the findings also provide examples of a rights-based approach to programming to promote sexual health among this population [8,9].

### Providing high-quality services for family planning

Of the 149 citations that were retrieved from the peer-reviewed literature search in relation to family planning services for women living with HIV (as consistent with the WHO guideline this included family planning counselling, contraception and dual protection, sterilization, counselling for pregnancy planning, conception, etc.), 12 met the criteria for inclusion in this review. Taken together, the following key themes emerge: 1) the increased accessibility and availability of ART has profoundly impacted the ability of women living with HIV to realize their reproductive desires and rights although barriers still remain; 2) a wide range of experiences with healthcare providers has been reported and there is a demonstrated need to provide further clinical and rights-based training to providers to ensure women living with HIV can realize their reproductive desires and rights; 3) forced sterilizations continue to violate the rights of women living with HIV and must be addressed everywhere it occurs as a matter of urgency; and 4) recent court judgments are beginning to show promise in upholding the reproductive rights of women living with HIV although key barriers still remain [10–21].

The search of the grey literature found one report documenting cases of forced sterilization of women living with HIV in Chile, the Dominican Republic, Mexico, Namibia, South Africa and Venezuela [22]. The report on findings from a global survey on SRH and human rights of women living with HIV referenced earlier identifies a lack of quality care in family planning services for women living with HIV and calls for compassionate, holistic, unconditional care and support for informed choice [9]. Findings from these reports echo the demonstrated need to provide further rights-based training to providers to ensure women living with HIV can realize their reproductive desires and rights.

### Improving antenatal, intrapartum, postpartum and newborn care

Of the 233 citations that were retrieved from the peer-reviewed literature search in relation to antenatal, intrapartum, postpartum and newborn care (as consistent with the WHO guideline this included counselling during pregnancy, childbirth and the postpartum period; preventing HIV infection among infants; and skilled care during pregnancy, childbirth, and postpartum period), only six met the criteria for inclusion in this review. Taken together, the following themes emerge: while ART and related efforts have significantly reduced transmission of HIV from mothers to infants, 1) there remains inadequate attention to the challenges with disclosure and the attitudes of providers in the context of pregnancy, childbirth and postpartum care; and 2) despite some attention to human rights in prevention of mother-to-child transmission (PMTCT) programmes, more research is needed as to the potentially positive impacts of human rights-based approaches in PMTCT as well as other antenatal, intrapartum, postpartum and newborn care programmes [3,23–27].

The search of the grey literature yielded one report by Amnesty International in relation to antenatal care in South Africa which identified cases of discrimination and abuses by healthcare workers (including breaches of privacy and confidentiality) faced by women living with HIV. The findings in this report once again affirm that there remains inadequate attention to the discrimination faced by women living with HIV particularly in the context of pregnancy and childbirth [28].

### Eliminating unsafe abortion

While only three citations were retrieved from the peer-reviewed literature search in relation to abortion (as consistent with the WHO guideline this included abortion counselling, surgical and medical methods of abortion, post-abortion care and family planning), after full-text review of all articles meeting the inclusion criteria for review, five were ultimately found to be relevant to abortion. Taken together, the following key themes emerge: 1) restrictive abortion laws fundamentally infringe upon the reproductive rights of women living with HIV often resulting in the need to seek unsafe abortion; and 2) even in settings where abortion law is relatively liberal, there is a need for attention to other reasons that may result in women living with HIV needing to seek unsafe abortion including stigma and discrimination, socio-economic status and health service implementation factors [29–33].

The report on findings from a global survey on SRH and human rights of women living with HIV referenced earlier identifies a lack of informed consent in reproductive decision making. Findings suggest that more consistency is needed among global, regional and national policy and programming documents to ensure women living with HIV have access to safe abortion services and are able to exercise their reproductive rights [9].

### Combating STIs, RTIs and cervical cancer

Of the 252 citations that were found from the peer-reviewed literature search in relation to STIs, RTIs and cervical cancer (as consistent with the WHO guideline this included STIs and RTIs, screening for STIs, comprehensive case management, cervical cancer screening and treatment, etc.), only one article ultimately met the criteria for inclusion in the review. This study suggested the need to pay attention to rights in relation to reducing STI prevalence, improving service delivery and helping to establish an enabling legal and policy environment. No article made an explicit linkage between denial or promotion of rights and research findings. Overall, the limited literature on human rights and STIs, RTIs and cervical cancer affecting women living with HIV suggests the need for additional research with explicit attention to these linkages [34].

The search of the grey literature did not retrieve any documents specifically on this topic, echoing the need for additional research and documentation with explicit attention to these linkages.

### Reducing violence

Of the 53 citations that were retrieved from the peer-reviewed literature search in relation to violence among women living with HIV (as consistent with the WHO guideline this included sexual violence, physical assault and psychological violence), only one article had a primary focus on violence. This suggests an urgent need for research which considers the linkages between human rights; the various forms of violence, including gender-based violence, intimate partner violence, and structural forms of violence; and how these act as key barriers to HIV prevention, access, treatment and health outcomes for women living with HIV [35].

The search of the grey literature yielded a report of an initiative in Kenya which emphasizes the importance of community participation in addressing the links between HIV and gender-based violence [36]. In addition, the global survey report on SRH and human rights of women living with HIV referenced earlier identified the human rights dimensions of the violence experienced in homes, communities and institutions (including health services) and calls for safety in all of these settings [9]. Finally, the grey literature search yielded a guide for programme managers on integrating human rights into gender-based violence and HIV services [37]. While limited, these documents can offer insights into the need for and rights-based approaches to reduce violence among women living with HIV although more evidence is clearly needed in this area.

### Promoting healthy ageing

Of the 73 citations that were found from the peer-reviewed literature search in relation to ageing among women living with HIV, none gave attention to its human rights dimensions.

The search of the grey literature only retrieved the findings from the global survey on SRH and human rights of women living with HIV referenced earlier [9]. Respondents reported sizeable gaps in clinical care, practice, policy and research for women and girls outside the reproductive years and/or not wanting to have children and made a request for health services to adopt a holistic, women-centred con(tra)ception to old-age approach to sexual and reproductive healthcare, with a comprehensive package of age and stage-appropriate (i.e. infancy, adolescence, adulthood) services. This echoes the need for additional attention to human rights in relation to ageing for women living with HIV.

### Integrated models of care

Of the 40 articles included in this review from the peer-reviewed literature search, four emphasized integrated models of care as relevant to the SRH and rights of women living with HIV – three discussed integrated SRH and HIV services while another specifically discussed the potential synergies of integrating maternal health and HIV services. While some studies found clear consensus among decision makers on the need for more integrated systems of SRH and HIV care and the effectiveness integrated care can provide, a study from Swaziland suggested patients may experience increased felt-stigma in integrated settings. Taken together, the literature in this area suggests the following themes: 1) while integrated models of care likely provide opportunities to reduce discrimination and stigma for women living with HIV and improve accessibility and quality of care, results are not uniformly positive; and 2) rights-based approaches to SRH and HIV remain to be better defined but can elucidate both shared determinants and solutions in improving service delivery for and health outcomes of women living with HIV [38–41].

The search of the grey literature yielded one report addressing integrated models of care as relevant to the sexual and reproductive health and rights (SRHR) of women living with HIV. The report documents case studies of successful approaches at the intersection of SRHR and HIV innovated and/or led by women living with HIV with the intention of strengthening in practice the integration of services. Among the case studies are examples of projects integrating SRH and HIV services in the city of Memphis within the United States, programmes in Russia and Malawi reducing violence against women living with HIV, and home-based care for women and girls living with HIV in Kenya [42]. The report echoes that integrated models of care may improve accessibility and quality of care for women living with HIV although more research is needed.

### Structural, societal and contextual factors impacting SRH and rights

Of the 40 articles included in our review of the peer-reviewed literature, eight can be considered to have raised larger structural, societal and contextual factors impacting the SRH and rights of women living with HIV. Taken together, the literature in this area suggests the following themes: 1) gender inequality remains a key barrier to be addressed to improve the SRH of women living with HIV, with potential impacts also on all aspects of HIV prevention, access and treatment; and 2) discrimination against women living with HIV, and particularly women from key populations (in particular sex workers, injecting drug users and transgender women), continues to be pervasive and influence health behaviours, care-seeking, adherence to treatment and health outcomes [43–50].

Findings from all of the grey literature reviewed provide support and depth consistent with the conclusions above [8,9,22,28,36,37,42].

## Conclusions

Human rights increasingly form part of the language and approach of international organizations, governments, non-governmental organizations and civil society groups concerned with the SRH of women living with HIV. Most, if not all, States have committed themselves to promote and protect relevant human rights by ratifying international and/or regional human rights instruments, and making political commitments [51]. Beyond rhetorical commitments, the on-the-ground implications of a commitment to human rights for the lives and health of women living with HIV remain unclear. Even when efforts are made by States to improve the SRH of women living with HIV in line with their human rights commitments, legal, policy, structural and systems barriers continue to exist both inside and outside the health sector. Further, even as efforts are increasingly being made by a number of different actors to bring human rights into relevant SRH programming, documentation is inadequate and inconsistent. While there are tremendous strides taking place at the normative level and very important work happening on the ground, within the literature there are few well-documented examples of bringing human rights into the work undertaken to support the SRH of women living with HIV.

In this review, we found the amount of peer-reviewed literature to directly address human rights and the SRH of women living with HIV to be far more limited than expected in terms of quantity, and what does exist only addresses a few rights in the context of a few areas within SRH. Once the inclusion criteria were applied, the search yielded few articles. Even within the literature that met the criteria for inclusion, the language of rights is used most often to describe their apparent neglect or violation rather than their promotion or inclusion in programming or services. Several articles do point to the need to better integrate rights into interventions, particularly as it concerns provider training, raising client awareness and service delivery. From issues related to privacy and confidentiality to forced sterilization, awareness and integration of human rights in provider training is of critical importance. Increased education and information incorporating human rights is necessary for women living with HIV to claim their rights and make informed decisions regarding their healthcare. Finally, additional efforts must be made to test, validate and write up the inclusion of rights in comprehensive, quality care and service delivery models geared towards women living with HIV.

Similarly, the available and accessible grey literature in this search that directly addresses the application of human rights to address the SRH of women living with HIV is also limited. While a handful of examples were found, the grey literature search did not identify significant existing evidence on interventions to address/promote human rights in the context of SRH programmes and policies for women living with HIV. Rather, the grey literature offers significant examples of violations of rights and a few case studies of successful interventions. These give context and substance by providing information that demonstrates the SRH rights and needs of women living with HIV are real issues to be addressed.

While rights may be explicitly addressed in policies relevant to the lives of women living with HIV, there was not a single peer-reviewed article found through this review that described the conscious and explicit implementation of rights concepts in programming to address the SRH of women living with HIV. With the possible exception of one article by Fried *et al*. [40], none were found which addressed rights comprehensively, or addressed the majority of rights considered central to a rights-based approach, with only a handful addressing particular rights within a SRH topic. Several articles were included because it was determined that they met the criteria for implicit use of rights, particularly in relation to different aspects of the 3AQ, but it remains unclear if researchers had consciously brought rights concepts into these interventions, if they would as a consequence have been shaped differently and ultimately would have been more effective in health and rights terms. While it can be argued that articles and documents excluded from our review might also have raised rights implicitly to some degree, in particular the literature found on stigma, these lacked a sufficiently explicit link to human rights.

In sum, there appears to be a significant disconnect between those who are implementing rights-based interventions and those who are publishing in the peer-reviewed and grey literature. What can be found in the literature is limited, insufficiently funded and ultimately inadequate to represent the evidence base in this area. While it is possible that there may be wonderful examples of implementation of rights-based approaches to SRH of women living with HIV happening on the ground, this is not reflected in the literature, be it peer-reviewed or grey. There is an urgent need for more research in this area and to conduct rigorous interviews with institutions and organizations implementing rights-based interventions geared to supporting the SRH and rights of women living with HIV, including in-depth interviews with key informants working with women living with HIV (e.g. International Community of Women Living with HIV/AIDS (ICW), Global Coalition on Women and AIDS (GCWA), Athena, among others). Along with more research, attention to the normative work happening both globally and within regions and countries is warranted. Most importantly, the voices and experiences of women living with HIV must ultimately frame the discussion and inform evidence-based guidelines to improve the treatment, care and support of women living with HIV.

## References

[1] WHO (2006). Guideline on sexual and reproductive health of women living with HIV/AIDS: guidelines on care, treatment and support for women living with HIV/AIDS and their children [Internet].

[2] WHO (2014). Framework for ensuring human rights in the provision of contraceptive information and services [Internet].

[3] MacCarthy S, Rasanathan JJK, Ferguson L, Gruskin S (2012). The pregnancy decisions of HIV-positive women: the state of knowledge and way forward. Reprod Health Matters.

[4] Khosla R, Van Belle N, Temmerman M (2015). Advancing the sexual and reproductive health and human rights of women living with HIV: A review of UN, regional and national human rights norms and standards. J Int AIDS Soc.

[5] MacGregor H, Mills E (2011). Framing rights and responsibilities: accounts of women with a history of AIDS activism. BMC Int Health Hum Rights.

[6] Bott S, Obermeyer CM (2013). The social and gender context of HIV disclosure in sub-Saharan Africa: a review of policies and practices. SAHARA J.

[7] Mykhalovskiy E (2011). The problem of “significant risk”: exploring the public health impact of criminalizing HIV non-disclosure. Soc Sci Med.

[8] Kundu NK (2011). SANGRAM's collectives. Engaging communities in India to demand their rights: Arlington, Virginia, John Snow [JSI], AIDS Support and Technical Assistance Resources [AIDSTAR-One].

[9] Salamander Trust (2014). Building a safe house on firm ground: key findings from a global values and preferences survey regarding the sexual and reproductive health and human rights of women living with HIV.

[10] van Dijk MG, Wilson KS, Silva M, Contreras X, Fukuda HD, Garcia SG (2014). Health care experiences of HIV-infected women with fertility desires in Mexico: a qualitative study. J Assoc Nurses AIDS Care.

[11] Kendall T (2013). Falling short of universal access to reproductive health: unintended pregnancy and contraceptive use among Mexican women with HIV. Cult Health Sex.

[12] Chimphamba Gombachika B, Chirwa E, Malata A, Sundby J, Fjeld H (2013). Reproductive decisions of couples living with HIV in Malawi: what can we learn for future policy and research studies?. Malawi Med J.

[13] Strode A, Mthembu S, Essack Z (2012). “She made up a choice for me”: 22 HIV-positive women's experiences of involuntary sterilization in two South African provinces. Reprod Health Matters.

[14] Essack Z, Strode A (2012). “I feel like half a woman all the time”: the impacts of coerced and forced sterilisations on HIV-positive women in South Africa. Agenda.

[15] Badul CJ, Strode A (2013). LM and others v Government of the Republic of Namibia: the first sub-Saharan African case dealing with coerced sterilisations of HIV-positive women – Quo vadis?. Afr Hum Rights Law J.

[16] Laar AK (2013). Reproductive rights and options available to women infected with HIV in Ghana: perspectives of service providers from three Ghanaian health facilities. BMC Womens Health.

[17] Schaan MM, Taylor M, Puvimanasinghe J, Busang L, Keapoletswe K, Marlink R (2012). Sexual and reproductive health needs of HIV-positive women in Botswana – a study of health care worker's views. AIDS Care.

[18] Anderson G, Caswell G, Edwards O, Hsieh A, Hull B, Mallouris C (2012). Community voices: barriers and opportunities for programmes to successfully prevent vertical transmission of HIV identified through consultations among people living with HIV. J Int AIDS Soc.

[19] Erhabor O, Akani CI, Eyindah CE (2012). Reproductive health options among HIV-infected persons in the low-income Niger Delta of Nigeria. HIV AIDS (Auckl).

[20] Messersmith LJ, Semrau K, Anh TL, Trang NN, Hoa DM, Eifler K (2012). Women living with HIV in Vietnam: desire for children, use of sexual and reproductive health services, and advice from providers. Reprod Health Matters.

[21] Loutfy M, Sonnenberg-Schwan U, Margolese S, Sherr L (2013). on behalf of Women for Positive Action. A review of reproductive health research, guidelines and related gaps for women living with HIV. AIDS Care.

[22] Open Society Institute (2011). Against her will: forced and coerced sterilization of women worldwide.

[23] Eamer GG, Randall GE (2013). Barriers to implementing WHO's exclusive breastfeeding policy for women living with HIV in sub-Saharan Africa: an exploration of ideas, interests and institutions. Int J Health Plann Manage.

[24] Ghanotakis E, Peacock D, Wilcher R (2012). The importance of addressing gender inequality in efforts to end vertical transmission of HIV. J Int AIDS Soc.

[25] Clouse K, Schwartz S, Van Rie A, Bassett J, Yende N, Pettifor A (2014). “What they wanted was to give birth; nothing else”: barriers to retention in option B plus HIV care among postpartum women in South Africa. J Acquir Immune Defic Syndr.

[26] Madhivanan P, Krupp K, Kulkarni V, Kulkarni S, Vaidya N, Shaheen R (2014). HIV testing among pregnant women living with HIV in India: are private healthcare providers routinely violating women's human rights?. BMC Int Health Hum Rights.

[27] Spangler SA, Onono M, Bukusi EA, Cohen CR, Turan JM (2014). HIV-positive status disclosure and use of essential PMTCT and maternal health services in rural Kenya. J Acquir Immune Defic Syndr.

[28] Amnesty International Struggle for maternal health: barriers to antenatal care in South Africa.

[29] Orner P, Bruyn MD, Cooper D (2011). ‘It hurts, but I don't have a choice, I'm not working and I'm sick’: decisions and experiences regarding abortion of women living with HIV in Cape Town, South Africa. Cult Health Sex.

[30] Orner PJ, de Bruyn M, Barbosa RM, Boonstra H, Gatsi-Mallet J, Cooper DD (2011). Access to safe abortion: building choices for women living with HIV and AIDS. J Int AIDS Soc.

[31] Barbosa RM, Pinho AA, Santos NS, Villela WV (2012). Exploring the relationship between induced abortion and HIV infection in Brazil. Reprod Health Matters.

[32] MacCarthy S, Rasanathan JJK, Crawford-Roberts A, Dourado I, Gruskin S (2014). Contemplating abortion: HIV-positive women's decision to terminate pregnancy. Cult Health Sex.

[33] de Bruyn M (2012). HIV, unwanted pregnancy and abortion – where is the human rights approach?. Reprod Health Matters.

[34] Paz-Bailey G, Fernandez VI, Morales Miranda S, Jacobson JO, Mendoza S, Paredes MA (2012). Unsafe sexual behaviours among HIV-positive men and women in Honduras: the role of discrimination, condom access, and gender. Sex Transm Dis.

[35] Gibbs A, Mushinga M, Crone ET, Willan S, Mannell J (2012). How do national strategic plans for HIV and AIDS in southern and eastern Africa address gender-based violence? A women's rights perspective. Health Hum Rights.

[36] Jain S, Greene M, Douglas Z, Betron M, Fritz K (2011). Addressing HIV and gender from the ground up. Maanisha Community Focused Initiative to Control HIV: a program to build the capacity of civil society organizations in Kenya: Arlington, Virginia, John Snow [JSI], AIDS Support and Technical Assistance Resources [AIDSTAR-One].

[37] Khan A (2011). Gender-based violence and HIV: a program guide for integrating gender-based violence prevention and response in PEPFAR programs: Arlington, Virginia, John Snow [JSI], AIDS Support and Technical Assistance Resources [AIDSTAR-One].

[38] Church K, Wringe A, Fakudze P, Kikuvi J, Simelane D, Mayhew SH (2013). Are integrated HIV services less stigmatizing than stand-alone models of care? A comparative case study from Swaziland. J Int AIDS Soc.

[39] Smit JA, Church K, Milford C, Harrison AD, Beksinska ME (2012). Key informant perspectives on policy- and service-level challenges and opportunities for delivering integrated sexual and reproductive health and HIV care in South Africa. BMC Health Serv Res.

[40] Fried S, Harrison B, Starcevich K, Whitaker C, O'Konek T (2012). Integrating interventions on maternal mortality and morbidity and HIV: a human rights-based framework and approach. Health Hum Rights.

[41] Barroso C, Sippel S (2011). Sexual and reproductive health and rights: integration as a Holistic and rights-based response to HIV/AIDS. Women's Health Issues.

[42] Orza L (2011). Community innovation: achieving sexual and reproductive health and rights for women and girls through the HIV response.

[43] Gibbs A, Crone ET, Willan S, Mannell J (2012). The inclusion of women, girls and gender equality in National Strategic Plans for HIV and AIDS in southern and eastern Africa. Global Public Health.

[44] Logie CH, James L, Tharao W, Loutfy MR (2011). HIV, gender, race, sexual orientation, and sex work: a qualitative study of intersectional stigma experienced by HIV-positive women in Ontario, Canada. PLoS Medi.

[45] Logie CH, James L, Tharao W, Loutfy MR (2012). “We don't exist”: a qualitative study of marginalization experienced by HIV-positive lesbian, bisexual, queer and transgender women in Toronto, Canada. J Int AIDS Soc.

[46] Marsicano E, Dray-Spira R, Lert F, Aubriere C, Spire B, Hamelin C (2014). Multiple discriminations experienced by people living with HIV in France: results from the ANRS-Vespa2 study. AIDS Care.

[47] Malavé S, Ramakrishna J, Heylen E, Bharat S, Ekstrand ML (2014). Differences in testing, stigma, and perceived consequences of stigmatization among heterosexual men and women living with HIV in Bengaluru, India. AIDS Care.

[48] King EJ, Maman S, Bowling JM, Moracco KE, Dudina V (2013). The influence of stigma and discrimination on female sex workers’ access to HIV services in St. Petersburg, Russia. AIDS Behav.

[49] Beyrer C, Baral S, Kerrigan D, El-Bassel N, Bekker L-G, Celentano DD (2011). Expanding the space: inclusion of most-at-risk populations in HIV prevention, treatment, and care services. J Acquir Immune Defic Syndr.

[50] Malta M, Beyrer C (2013). The HIV epidemic and human rights violations in Brazil. J Int AIDS Soc.

[51] Center for Reproductive Rights (2013). ICPD and human rights: 20 years of advancing reproductive rights though UN treaty bodies and legal reform [Internet].

# Sub article: Sexual inactivity and sexual satisfaction among women living with HIV in Canada in the context of growing social, legal and public health surveillance

**DOI:** 10.7448/IAS.18.6.20284

**Published:** 2015-12-01

**Authors:** Angela Kaida, Allison Carter, Alexandra de Pokomandy, Sophie Patterson, Karène Proulx-Boucher, Adriana Nohpal, Paul Sereda, Guillaume Colley, Nadia O'Brien, Jamie Thomas-Pavanel, Kerrigan Beaver, Valerie J Nicholson, Wangari Tharao, Mylène Fernet, Joanne Otis, Robert S Hogg, Mona Loutfy, on behalf of the CHIWOS Research Team

**Affiliations:** 1 Faculty of Health Sciences, Simon Fraser University, Burnaby, British Columbia, Canada; 2 British Columbia Centre for Excellence in HIV/AIDS, Vancouver, British Columbia, Canada; 3 Chronic Viral Illness Service, McGill University Health Centre, Montreal, Quebec, Canada; 4 Department of Family Medicine, McGill University, Montreal, Quebec, Canada; 5 Women's College Research Institute, Women's College Hospital, Toronto, Ontario, Canada; 6 Women's Health in Women's Hands Community Health Centre, Toronto, Ontario, Canada; 7 Département de Sexologie, Université du Québec à Montréal, Montréal, Quebec, Canada; 8 Department of Medicine and Institute of Health Policy, Management and Evaluation, University of Toronto, Toronto, Ontario, Canada

**Keywords:** HIV, women, Canada, sexual and reproductive health, sexual abstinence, sexual satisfaction, community-based research, antiretroviral therapy, CHIWOS

## Abstract

**Introduction:**

Women represent nearly one-quarter of the 71,300 people living with HIV in Canada. Within a context of widespread HIV-related stigma and discrimination and on-going risks to HIV disclosure, little is known about the influence of growing social, legal and public health surveillance of HIV on sexual activity and satisfaction of women living with HIV (WLWH).

**Methods:**

We analyzed baseline cross-sectional survey data for WLWH (≥16 years, self-identifying as women) enrolled in the Canadian HIV Women's Sexual and Reproductive Health Cohort Study (CHIWOS), a multisite, longitudinal, community-based research study in British Columbia (BC), Ontario (ON) and Quebec (QC). Sexual inactivity was defined as no consensual sex (oral or penetrative) in the prior six months, excluding recently postpartum women (≤6 months). Satisfaction was assessed using an item from the Sexual Satisfaction Scale for Women. Multivariable logistic regression analysis examined independent correlates of sexual inactivity.

**Results:**

Of 1213 participants (26% BC, 50% ON, 24% QC), median age was 43 years (IQR: 35, 50). 23% identified as Aboriginal, 28% as African, Caribbean and Black, 41% as White and 8% as other ethnicities. Heterosexual orientation was reported by 87% of participants and LGBTQ by 13%. In total, 82% were currently taking antiretroviral therapy (ART), and 77% reported an undetectable viral load (VL<40 copies/mL). Overall, 49% were sexually inactive and 64% reported being satisfied with their current sex lives, including 49% of sexually inactive and 79% of sexually active women (*p*<0.001). Sexually inactive women had significantly higher odds of being older (AOR=1.06 per year increase; 95% CI=1.05–1.08), not being in a marital or committed relationship (AOR=4.34; 95% CI=3.13–5.88), having an annual household income below $20,000 CAD (AOR: 1.44; 95% CI=1.08–1.92), and reporting high (vs. low) HIV-related stigma (AOR=1.81; 95% CI=1.09–3.03). No independent association was found with ART use or undetectable VL.

**Conclusions:**

Approximately half of WLWH in this study reported being sexually inactive. Associations with sexual dissatisfaction and high HIV-related stigma suggest that WLWH face challenges navigating healthy and satisfying sexual lives, despite good HIV treatment outcomes. As half of sexually inactive women reported being satisfied with their sex lives, additional research is required to determine whether WLWH are deliberately choosing abstinence as a means of resisting surveillance and disclosure expectations associated with sexual activity. Findings underscore a need for interventions to de-stigmatize HIV, support safe disclosure and re-appropriate the sexual rights of WLWH.

## Introduction

Globally, women account for over half of all adults living with HIV [1]. In Canada, approximately one-quarter of the 71,300 people living with HIV (PLWH) are women, nearly double the proportion observed in 1999 (12%) [2]. With early and sustained use of antiretroviral therapy (ART), women living with HIV (WLWH) are living longer and healthier lives [3–5] with improved sexual and reproductive options accompanying lowered risks of sexual and perinatal HIV transmission [6–8]. This altered landscape of HIV risk has re-ignited global discourse regarding the need for a rights-based approach to sexual health [9–12]. Sexual health research and programming targeting WLWH, however, are largely focused on risk behaviours (such as condom use) rather than broader sex-positive considerations of sexual intimacy, well-being and satisfaction [13,14]. Moreover, prevailing concern about individual-level behaviours ignores the broader clinical, legal and social factors that regulate the sexual lives and rights of WLWH [15,16].

The criminalization of HIV non-disclosure [17] and “Treatment as Prevention” (TasP) [18] are two legal and public health strategies aimed at preventing HIV transmission that have emerged in many global settings, including Canada [11,19–21]. While both initiatives place sexual activity of PLWH and “Positive Prevention” (HIV prevention strategies directed at PLWH) at the centre of their efforts, they are theoretically opposing and deviate from the “shared responsibility” messaging for HIV prevention endorsed by the Global Network of People Living with HIV (GNP+), the Canadian AIDS Society and other international agencies [22,23]. Growing evidence demonstrates that early and sustained use of ART for greater than six months with viral suppression generates a very low risk of HIV transmission during condomless sex between HIV sero-discordant sexual partners, with recent studies reporting a transmission risk approaching zero [24–26]. These findings have supported the implementation of TasP initiatives in a number of global jurisdictions [20,27,28], where PLWH are monitored along a care cascade from HIV diagnosis to linkage and retention in care, and initiation and adherence to ART to achieve sustained viral suppression [29,30].

Despite the evidence and growing optimism about what it means to live with HIV, in terms of improved quality of life, clinical health and lowered transmission risks, Canada has among the most aggressive judicial approaches to prevent perceived sexual exposure to HIV through the criminalization of HIV non-disclosure [19,21]. In October 2012, the Supreme Court of Canada ruled that PLWH are legally required to disclose their HIV status to sexual partners prior to sexual activity that poses a “realistic possibility” of HIV transmission [17,31]. The Supreme Court defined realistic possibility as any sexual activity without the use of a condom and without a low HIV plasma viral load (defined by the court as VL<1500 copies/mL). PLWH who fail to meet both criteria and do not disclose their HIV status to sexual partners risk a criminal charge of aggravated sexual assault. If convicted, this charge results in jail time with a maximum sentence of life imprisonment and mandatory listing on a national Sexual Offender Registry. The inconsistency between legal definitions of the “realistic possibility” of HIV transmission and contemporary scientific assessments of HIV transmission risk and prognosis detract from rights-based approaches to improving the sexual health of WLWH and propagate misconceptions about the sexual and reproductive realities of living with HIV.

For many WLWH around the world, such public health and legal HIV prevention strategies have introduced increased surveillance (including monitoring VL, HIV disclosure and condom use) and present attendant consequences to their sexual lives. Importantly, expectations of HIV status disclosure exist within a context of widespread HIV-related fear, violence, and stigma and discrimination, which when combined with gender and relationship power inequities, disproportionately compromise WLWH's navigation of intimate relationships [32–35]. Together, these social factors mute conversations about sexuality and HIV, deny risks that WLWH face with forced disclosure and/or condom use, compromise opportunities for safe disclosure and sexual relationships for WLWH and reduce the willingness and agency of WLWH to seek out critical health services [36]. In Canada and elsewhere, increasing use of ART has not substantially alleviated the presence or impact of stigma and discrimination [37] despite attempts to normalize HIV through initiatives under the TasP umbrella [38,39].

In response to growing social, legal and public health surveillance, WLWH may adopt various strategies to protect and navigate their sexual lives, including the avoidance of sexual and romantic partnerships. Indeed, previous research has suggested that WLWH may forgo sexual activity to avoid expectations and risks of HIV disclosure to sexual partners [32–34]. To understand drivers of sexual activity decision-making among WLWH in Canada, we measured the prevalence of sexual inactivity and sexual satisfaction among a cohort of WLWH and assessed demographic, HIV clinical and socio-structural correlates of sexual inactivity.

## Methods

### Study setting

In Canada, approximately 16,600 women were living with HIV in 2011 [40], of whom 81% lived in one of the three provinces: British Columbia (BC), Ontario or Quebec.

### Study design

Baseline cross-sectional data were analyzed for WLWH enrolled in the Canadian HIV Women's Sexual and Reproductive Health Cohort Study (CHIWOS; www.chiwos.ca), a large, multisite, longitudinal, community-based research (CBR) project conducted by, with and for WLWH in BC, Ontario and Quebec (full cohort: *n*=1427). The primary objective of CHIWOS is to assess the prevalence, barriers and facilitators to use of women-centred HIV care, and the impact of such patterns of use on health outcomes. CHIWOS is grounded in Critical Feminist theory [41] and CBR principles, and guided by a Social Determinants of Women's Health framework [42,43] (described in detail elsewhere [44]). Consistent with CBR methodology, WLWH and allied clinicians, researchers, community partners, social and public health service delivery personnel, and policy-makers were involved in all stages of this research.

### Study population and recruitment

This analysis includes participants enrolled in CHIWOS between 27 August 2013 and 13 March 2015. WLWH aged ≥16 years from one of the three enrolling study provinces were eligible to participate. CHIWOS is inclusive to self-identified trans and cis gendered women, two-spirited women, gender queer and women of other gender identities. Given the importance of including young women's voices in the study, an exemption was sought and approved such that parental/guardian consent for women aged 16–18 years was not required.

WLWH were recruited for participation through peer word-of-mouth, HIV clinics in large and small communities across the three provinces, AIDS Service Organizations (ASOs), non-HIV community-based organizations (e.g. immigration and refugee services, women's shelters, harm reduction and sex worker support services), the networks of our national Steering Committee and three provincial Community Advisory Boards, and online methods (e.g. Listservs for WLWH, and our study website (www.chiwos.ca), Facebook page (www.facebook.com/CHIWOS) and Twitter feed (www.twitter.com/CHIWOSresearch).

Consistent with data showing an over-representation of Aboriginal and African, Caribbean and Black (ACB) women among WLWH in Canada, targeted efforts were made to recruit Aboriginal and ACB participants. These efforts included hiring a diverse national team of 38 WLWH as Peer Research Associates (PRAs), including 6 Aboriginal and 14 ACB PRAs, and the creation of a CHIWOS Aboriginal Advisory Board whose members assisted and advised on community outreach and recruitment.

Overall, 1355 women were enrolled in CHIWOS at time of analysis. For this analysis, 142 (10%) women were excluded, including 84 who requested to skip the Sexual Health section of the survey, 7 who reported a live birth at or less than six months prior to interview, 40 who reported never having had consensual sex, and 13 who were missing data on recent consensual sex (the primary outcome), yielding an analytic sample of 1213 WLWH. Given our objective to measure intentional sexual activity, women who experienced non-consensual sex in the six months prior to interview without report of consensual sexual activity, were not considered sexually active.

### Data collection

All participants completed a structured, online questionnaire (supported by FluidSurveys™ software) at baseline (study enrolment), administered by PRAs (WLWH hired as members of the study team). PRAs underwent extensive training in research ethics, CBR, consenting, administering questionnaires, social positioning, self-care and support for participants [45]. Questionnaires were conducted in English or French and administered in-person in a confidential setting at collaborating HIV clinics, ASOs or community organizations, or in women's homes. For some participants in rural or remote areas, questionnaires were administered via phone or Skype. Median survey completion time was 89 minutes [IQR: 71, 115] and participants received a $50 honorarium for their participation, regardless of whether all questions or sections of the questionnaire were completed.

### Measures

A national team of experts in women's health and HIV contributed to the development of the CHIWOS questionnaire, which was designed to maximize psychometric validity and reliability [46] and collected information on socio-demographics, HIV medical history and clinical care, experiences of violence and stigma and discrimination, and sexual, reproductive, women's and emotional health outcomes. Given the sensitive nature of questions about sexual health and violence, participants were given the option of completing these survey sections with the PRA, independently, or skipping the sections entirely.

#### Primary outcomes: sexual inactivity and sexual satisfaction

The primary outcome was recent sexual inactivity (vs. sexual activity) defined as no (vs. any) consensual sex (oral or penetrative) in the six months prior to interview.

Among all women, regardless of recent sexual activity or inactivity, sexual satisfaction was assessed using a five-point Likert scale item from the Sexual Satisfaction Scale for Women (SSS-W) [47]: “Overall, how satisfactory or unsatisfactory is your present sex life.” Responses were dichotomized into satisfactory (“Completely/Very/Reasonably satisfactory”) vs. unsatisfactory (“Not very/Not at all satisfactory”).

#### Covariates

Covariates of sexual inactivity included socio-demographic characteristics (i.e. age, education, gender identity, sexual orientation, ethnicity, annual household income, relationship status, and number of children); self-reported clinical HIV history and outcomes, including years living with HIV, receipt of HIV medical care in past year (yes vs. no), receipt of ART in past year (yes vs. no), most recent VL (undetectable (<40 copies/mL) vs. detectable ≥40 copies/mL, shown to have a high positive predictive value [48]), most recent CD4 cell count (<200 cells/mm^3^ vs. 200–500 cells/mm^3^ vs. >500 cells/mm^3^), as well as having ever discussed the impact of VL on risk of HIV transmission with a healthcare provider (yes vs. no) and perceptions of how ART changes personal risk of transmitting HIV (lowers the risk of transmission vs increases the risk of transmission or makes little difference); the SF-12 physical and mental health-related quality of life summary scores (scored on a 0–100 scale, where a higher score indicates better health) [49,50]; depression (measured using the 10-item Center for Epidemiologic Studies Depression Scale (CES-D 10), scored on a 0–30 scale, with higher scores indicating greater depression symptomology and a cutoff score of ≥10 considered indicative of “probable depression”) [51,52]; and HIV-related stigma measured using the 10-item HIV Stigma Scale (HSS) [53,54]. Scores for the HSS range from 0 to 100, with higher scores indicating higher stigma. Scores greater or equal to the scale median were considered “high HIV-related stigma” vs. “low HIV-related stigma.”

Among sexually active women, we describe types of sexual partnerships and knowledge about HIV status. In the survey, “Regular Sexual Partners” were defined to include, but not limited to, “spouses, common law partners, long term relationships, friends with benefits, or partners seen on and off for some time.” “Casual Sexual Partners” were defined to include, but not limited to, “serious sexual relationships that have recently begun, new sexual relationships that exist but you're not sure about, chance sexual encounters, or one night stands.”

### Data analysis

Descriptive statistics (median Interquartile Range [IQR]) for continuous variables and n (%) for categorical variables) were used to characterize baseline distributions of study variables. Baseline differences between sexually inactive and sexually active women were compared using Wilcoxon rank sum test for continuous variables and Pearson χ^2^ or Fisher's exact test for categorical variables.

We fit a multivariable logistic regression explanatory model to the data to examine covariates of sexual inactivity. After testing normality assumptions and collinearity, variables with a significant association with sexual inactivity in bivariate analyses (at *p*<0.20) were considered for the full model to obtain the relative contribution of each covariate. Model selection was achieved by minimizing the Akaike Information Criterion while maintaining Type III *p*-values for covariates below 0.20 [55]. All statistical tests were two-sided and were considered statistically significant at α=0.05. Data were analyzed using SAS version 9.3 (SAS Institute, Inc., Cary, NC).

### Ethical statement

All participants provided written, voluntary informed consent (or oral consent with a study team member present as a witness for surveys conducted by phone or Skype) at enrolment in the survey phase of the CHIWOS study. Ethical approval for all study procedures was provided by the Research Ethics Boards of Simon Fraser University, University of British Columbia/Providence Health, Women's College Hospital and McGill University Health Centre.

## Results

### Baseline characteristics

Of 1213 participants, 26% were from BC, 50% from Ontario and 24% from Quebec. Median age was 43 years [IQR: 35, 50], 95% identified as cis gender women and 5% identified as trans women, two-spirited or gender queer. Eighty-seven percent were heterosexual and 13% identified as LGBTQ (Lesbian, Gay, Bi-Sexual, Two-spirited, or Queer). Nearly one-quarter (23%) identified as Aboriginal, 28% ACB, 41% White and 8% other ethnicities. Sixty-three percent had an annual household income <$20,000 CAD, and 34% were married, common law or in a relationship.

Median years living with HIV was 10.8 [IQR: 5.9, 16.8], 94% reported receiving HIV medical care in the past year and 82% were currently taking ART. By self-report, 77% had undetectable VL and 50% had CD4 cell count >500 cells/mm^3^ at most recent visit. Sixty-nine percent of participants reported discussing the impact of VL on HIV transmission risk with their healthcare provider, and overall, 81% perceived that use of antiretrovirals lowers HIV transmission risk. Median SF-12 physical and mental health summary scores were 48 [IQR: 33, 56] and 42 [IQR: 31, 52], respectively. Median HIV stigma score was 60 [IQR: 50, 73] with 48% classified as experiencing high (vs. low) HIV-related stigma. Median depression symptom severity score (based on CES-D) was 9 [IQR: 4, 15], with 49% meeting the criteria for probable depression (Table 1).

**Table 1:** Baseline characteristics of women living with HIV enrolled in CHIWOS overall and by sexual activity (*n*=1213)

	Overall (*n*=1213)		Sexually active (*n*=618)	Sexually inactive (*n*=595)	
			
Characteristic	*n* (%) or median [IQR]	Total *n*	*n* (%) or median [IQR]		*p*-value
Median age [IQR] (years)	43 [35, 50]	1213	40 [33, 46]	46 [38, 54]	<0.001
Age category (years)					
16 to <30	114 (9.4)	1213	83 (13)	31 (5.2)	<0.001
30 to <40	366 (30)		218 (35)	148 (25)	
40 to <50	392 (32)		215 (35)	177 (30)	
50+	341 (28)		102 (17)	239 (40)	
Province					
British Columbia (BC)	317 (26)	1213	172 (28)	145 (24)	0.009
Ontario	607 (50)		283 (46)	324 (55)	
Quebec	289 (24)		163 (26)	126 (21)	
Gender identity^a^					
cis gender woman	1157 (95)	1213	581 (94)	576 (97)	0.02
Trans woman, two-spirited, gender queer, or other gender	56 (5)		37 (6)	19 (3)	
Sexual orientation^b^					
Heterosexual	1050 (87)	1209	525 (85)	525 (88)	0.121
LGBTQ	159 (13)		90 (15)	69 (12)	
Ethnicity					
Aboriginal	277 (23)	1213	142 (23)	135 (23)	0.391
African, Caribbean and Black Canadian (ACB)	338 (28)		164 (27)	174 (29)	
White	502 (41)		268 (43)	234 (39)	
Other ethnicities	96 (8)		44 (7.1)	52 (8.7)	
Education					
<High school	180 (15)	1207	93 (15)	87 (15)	0.798
≥High school	1027 (85)		520 (85)	507 (85)	
Annual household income (CAD)					
≥$20,000	419 (35)	1213	242 (39)	177 (30)	<0.001
<$20,000	759 (63)		353 (57)	406 (68)	
Don't know/prefer not to answer	35 (3)		23 (3.7)	12 (2.0)	
Relationship status					
Single/separated/divorced/widowed	802 (66)	1207	306 (50)	496 (84)	<0.001
Married/common law/in a relationship	405 (34)		307 (50)	98 (17)	
Number of children					
0	372 (32)	1162	175 (30)	197 (34)	0.204
1–3	630 (54)		332 (57)	298 (52)	
4+	160 (14)		78 (13)	82 (14)	
Median time living with HIV [IQR] (years)	10.8 [5.9, 16.8]	1180	10.1 [5.6, 15.8]	11.7 [6.2, 17.6]	0.004
Received HIV medical care in the past year					
Yes	1139 (94)	1212	574 (93)	565 (95)	0.102
No	73 (6.0)		44 (7.1)	29 (4.9)	
Currently taking ART					
Yes	999 (82)	1213	500 (81)	499 (84)	0.176
No	214 (18)		118 (19)	96 (16)	
Current plasma viral load (self-reported)					
Detectable (≥40 copies/mL)	173 (14)	1213	91 (15)	82 (14)	0.869
Undetectable (<40 copies/mL)	939 (77)		477 (77)	462 (78)	
Don't know/never received results	101 (8.3)		50 (8.1)	51 (8.6)	
Current CD4 cell count (self-reported)					
<200 cells/mm^3^	66 (5.5)	1211	34 (5.5)	32 (5.4)	0.597
200–500 cells/mm^3^	329 (27)		166 (27)	163 (28)	
>500 cells/mm^3^	606 (50)		302 (49)	304 (51)	
Don't know/never received results	210 (17)		116 (19)	94 (16)	
Discussed VL on HIV transmission risk with healthcare provider					
Yes	826 (69)	1201	461 (75)	365 (62)	<0.001
No	375 (31)		151 (25)	224 (38)	
Perception of how ART changes HIV transmission risk					
Lowers risk of transmission	979 (81)	1213	509 (82)	470 (79)	0.214
Increases risk of transmission or makes little difference	130 (11)		57 (9.2)	73 (12)	
Don't know	104 (8.6)		52 (8.4)	52 (8.7)	
Median health-related quality of life score [IQR]					
Physical health summary score	48 [33, 56]	1206	50 [36, 56]	46 [32, 55]	0.004
Mental health summary score	42 [31, 52]	1206	42 [31, 52]	43 [32, 53]	0.537
HIV stigma scale					
High HIV-related stigma	575 (48)	1200	280 (46)	295 (50)	0.155
Low HIV-related stigma	625 (52)		330 (54)	295 (50)	
Satisfaction with present sex life					
Completely, very or reasonably satisfied	777 (64)	1213	488 (79)	289 (49)	<0.001
Not very or not at all satisfied	347 (29)		115 (19)	232 (39)	
Prefer not to answer	89 (7.3)		15 (2.4)	74 (12)	
Probable depression (CESD-10)					
No (score<10)	600 (51)	1173	330 (55)	270 (47)	0.007
Yes (score ≥10)	573 (49)		270 (45)	303 (53)	

Notes:

a Gender identity was self-reported as cis or trans gender woman, two-spirited, gender queer or an ‘other’ gender identity. Given small numbers, we grouped participants self-identifying as trans gender, two-spirited, gender queer or ‘other’ gender for this analysis. Of the 56 women included in this grouped category, 48 (86%) identified as transgender women.

b LGBTQ includes participants who identify as Lesbian, Gay, Bi-sexual, Two-spirited or Queer; CAD=Canadian dollars; ART=antiretroviral therapy; VL=viral load.

### Sexual inactivity, sexual satisfaction and summary of sexual partnerships

Forty-nine percent (49%) of participants reported being sexually inactive in the six months prior to baseline interview. Sixty-four percent (64%) of women reported being satisfied with their current sex lives, including 49% of sexually inactive women and 79% of sexually active women (*p*<0.001).

Of sexually active WLWH (*n*=618), 81% had one and 6% had two or more regular sexual partners in the previous six months. Of those with at least one regular sexual partner (*n*=543), 27% reported that their primary sexual partner was HIV-positive, 64% reported that the partner was HIV-negative and for 9% the partner's HIV status was unknown. Ninety-one percent (91%) reported that their current or most recent regular partner knew the participant's HIV status at their last sexual encounter. One-fifth (21%) of all sexually active participants reported having a casual sexual partner in the previous six months.

### Correlates of sexual inactivity

In unadjusted analyses, sexual inactivity was associated (at *p*<0.20) with older age, gender identity, sexual orientation, household income <$20,000 per year, not being in a martial or committed relationship, longer time living with HIV, current ART use, poorer physical health, high HIV-related stigma scores, probable depression, sexual dissatisfaction and having discussed the role of VL on HIV transmission risk with a healthcare provider (Table 2).

**Table 2:** Unadjusted and adjusted odds ratios for correlates of sexual inactivity in the previous six months among women living with HIV enrolled in CHIWOS

	Sexually inactive vs. Sexually active over the last six months	
	
Characteristics	Unadjusted OR (95% CI)	Adjusted OR (95% CI)
Age, per one year increase	1.06 (1.05–1.08)	1.06 (1.05–1.08)
Sexual orientation		
Heterosexual	1.0	
LGBTQ	0.75 (0.54–1.05)	
Annual household income (CAD)		
≥$20,000	1.0	1.0
<$20,000	1.57 (1.24–2.0)	1.44 (1.08–1.92)
Relationship status		
Married/common law/in a relationship	1.0	1.0
Single/separated/divorced/widowed	5.00 (3.84–6.67)	4.34 (3.13–5.88)
Currently taking ART		
Yes	1.0	
No	0.82 (0.61–1.10)	
Discussed VL on HIV transmission risk with healthcare provider		
Yes	1.0	1.0
No	1.87 (1.46–2.4)	1.57 (1.16–2.11)
HIV-related stigma		
Low HIV-related stigma	1.0	1.0
High HIV-related stigma	1.18 (0.94–1.47)	1.81 (1.09–3.03)
Probable depression (CESD-10)		
No (score <10)	1.0	
Yes (score ≥10)	1.37 (1.09–1.73)	
Physical Health Summary score	0.99 (0.98–0.99)	
Satisfaction with present sex life		
Completely, very, or reasonably satisfied	1.0	
Not very or Not at all satisfied	3.41 (2.61–4.45)	
Prefer not to answer	8.33 (4.69–14.8)	

Note: Model adjusted for Province of residence; LGBTQ includes participants who identify as Lesbian, Gay, Bi-sexual, Two-spirited or Queer; CAD=Canadian dollars; ART=antiretroviral therapy; VL=viral load.

In the logistic regression model, sexually inactive women had significantly higher adjusted odds of being older (AOR=1.06 per year increase in age; 95% CI=1.05–1.08), not being in a marital or committed relationship (AOR=4.34; 95% CI=3.13–5.88), having an annual household income below $20,000 CAD (AOR=1.44; 95% CI: 1.08–1.92), and reporting high (vs. low) HIV-related stigma (AOR=1.81; 95% CI=1.10–3.03). No independent association was found with current ART use, undetectable plasma HIV VL or CD4>500 cells/mL. However, sexually inactive women were significantly more likely to report not having discussed the role of VL on decreasing HIV transmission risk with a healthcare provider (AOR=1.57; 95% CI: 1.16–2.11; Table 2).

## Discussion

This is the first multisite cohort study to evaluate sexual inactivity among WLWH in Canada in the modern era of TasP and HIV criminalization. Nearly half of WLWH in this Canadian cohort reported being sexually inactive and over two-thirds report being satisfied with their sexual lives. Other studies have similarly shown that sexual inactivity is common among PLWH, particularly women. The Women's Interagency HIV Study (WIHS), an on-going cohort study of WLWH in the United States (US), found that 35% of WLWH reported no vaginal, oral or anal sex over a six-month time period compared with 23% of HIV-negative women [56]. Other US research has found that WLWH are significantly more likely to be sexually inactive (34%) compared with HIV-positive gay and bisexual men (28%) [57]. Similar results were seen in a study by Bogart *et al*. [58], where a higher proportion of WLWH (18%) were deliberately abstinent compared to gay and bisexual men with HIV (11%). The prevalence of sexual inactivity (49%) in our study was notably higher than estimates from other HIV cohorts. Some of this difference may be explained by the older median age of WLWH enrolled in CHIWOS compared with other referenced cohorts and the evidence that sexual activity and satisfaction decline with increasing age [59,60]; and shown in Table 1. Some difference may also be explained by our distinction between and exclusion of non-consensual sex in our definition of sexual activity. However, the remaining difference may be explained by a high reported prevalence of HIV stigma and the context of surveillance for WLWH in the Canadian setting [61]. Our estimates of sexual inactivity are also significantly higher than those reported in the general population (10–14%) [62], echoing previous research that an HIV diagnosis impacts the sexual health of women [63] even several years after the initial diagnosis [64].

The association between sexual inactivity and high HIV-related stigma and sexual dissatisfaction but not ART use or viral suppression suggests that WLWH face challenges navigating healthy, satisfying and safe sexual lives, despite good treatment outcomes. This suggests that good treatment outcomes alone do not lead to higher likelihood of sexual activity or eliminate prevailing socio-structural barriers to sexual health for WLWH.

HIV-related stigma and discrimination are critical barriers to cultivating loving, intimate relationships, facilitating HIV status disclosure and engaging with healthcare for WLWH [65,66]. The increasingly strict use of the criminal law for HIV non-disclosure against PLWH in Canada presents an additional form of stigmatization [67] and additional challenges for WLWH initiating sexual relationships and navigating HIV disclosure to sexual partners. This judicial approach shifts the responsibility for condom use and the burden of proof onto PLWH, introducing the potential for false accusations of HIV non-disclosure by sexual partners and concerns of secondary disclosure within the wider community [68,69]. Observations that the sexuality of PLWH is being policed [70] and reports of discriminating medical surveillance in the reproductive healthcare setting [61] raise concerns that the sexual rights of WLWH are being unjustly compromised in the current legal climate. While we are unable to directly assess the effect of criminalization of HIV non-disclosure on sexual activity within this study, qualitative studies in Canada have found that fear, anxiety and uncertainty related to the current legal obligation to disclose HIV serostatus to sexual partners have resulted in some PLWH abstaining from sexual activity altogether [70]. Forgoing sexual activity removes expectations and risks of HIV disclosure to sexual partners, difficulties negotiating condom use and risks of sexual HIV transmission. For some WLWH, sexual abstinence may also be a response to fear and threats of rejection by sexual partners, secondary disclosure, relationship dissolution, violence and social isolation following disclosure of HIV status [32,58,71,72].

Despite the high prevalence of sexual inactivity in our cohort, a large proportion of women overall report being satisfied with their current sex lives (64%). While satisfaction differs by sexual activity, it is higher overall than expected based on previous research [60]. Among sexually inactive women, half report being satisfied with their present sex lives, suggesting that sexual abstinence may be deliberate or intentional, not simply circumstantial. However, sexually inactive women report higher HIV-related stigma. This suggests that HIV-related stigma plays an important role in sexual decision-making, expression and lives of WLWH. Furthermore, WLWH experiencing high HIV-related stigma may be actively choosing sexual inactivity as a strategy to avoid having to navigate disclosure and risk stigma and discrimination in initiating sexual partnerships, and for many women this is a satisfactory approach to their sexual lives.

Interventions are required to reframe the approach to sexual health among WLWH. A component of this reframing is a movement towards models that advocate for Positive Health, Dignity and Prevention frameworks [23,73], which place the person living with HIV at the centre of their health, care and well-being, well beyond a role in “positive prevention” of on-going transmission of HIV. For example, the Au-delà du VIH: être femme Plurielle [Beyond HIV: being a woman in its multiple meanings] program in Quebec is grounded in an empowerment approach to sexual health for WLWH, and supports the capacity of WLWH to respond to their sexual needs and mobilize available resources to gain control over their sexual lives. This, and other similar interventions, recognize WLWH as sexual beings with sexual rights and aim to overcome beliefs and taboos of female sexuality and desire [74].

Limitations to this study are acknowledged. First, cross-sectional analyses preclude determination of causality between correlates and sexual inactivity, and reverse causality is possible (e.g. sexual inactivity leading to depression). Second, there is risk of reporting bias whereby women may underreport sexual activity because of prevailing stigma and discrimination against sexually active WLWH. However, surveys were administered by PRAs, a diverse group of WLWH with identities and life experiences in common with participants, which may have decreased the extent of social desirability bias in reports about sexual activity (with <10% of participants completing the sexual health section independently). Third, we are unable to report whether sexual inactivity represents deliberate or intentional abstinence for sex or a lack of opportunity for sex; however, this difference will be explored in the CHIWOS 18-month follow-up questionnaire. Finally, several important psychosocial aspects of sexual inactivity, including sensation seeking, desire and motivation, as well as knowledge and perceived effect of criminalization of HIV non-disclosure on sexual activity, were not directly assessed in this study, and are likely to be important explanatory pathways to understanding sexual inactivity among WLWH. These pathways will also be assessed in the follow-up questionnaire.

## Conclusions

A satisfying sexual life is a critical component of health and well-being for all people, including WLWH. The observed high rate of sexual inactivity coupled with associations with HIV-related stigma and sexual dissatisfaction underscores a need to revisit the narrative about sexual activity among WLWH. This narrative has focused heavily on HIV risk reduction approaches and now increasingly on the public health importance of viral suppression and the legal test for HIV non-disclosure, but much less has been said about healthy sexuality. A transitioning of the approach to healthy sexuality among WLWH requires that we finally erase the current view of WLWH as “vectors, vessels and victims” of HIV [75], rather as empowered individuals with agency and deserving of intimate relationships. These findings underscore an urgent need for public health and socio-structural interventions to de-stigmatize HIV, support safe disclosure and re-appropriate the sexual rights of WLWH.

## References

[1] UNAIDS The Gap Report 2014.

[2] Public Health Agency of Canada (2011). Estimates of HIV prevalence and incidence in Canada, 2011.

[3] Samji H, Cescon A, Hogg RS, Modur SP, Althoff KN, Buchacz K (2013). Closing the gap: increases in life expectancy among treated HIV-positive individuals in the United States and Canada. PLoS One.

[4] Hogg RS, Yip B, Chan KJ, Wood E, Craib KJP, O'Shaughnessy MV (2001). Rates of disease progression by baseline CD4 cell count and viral load after initiating triple-drug therapy. J Am Med Assoc.

[5] Patterson S, Cescon A, Samji H, Chan K, Zhang W, Raboud J (2015). Life expectancy of HIV-positive individuals on combination antiretroviral therapy in Canada. BMC Infect Dis.

[6] Forbes JC, Alimenti AM, Singer J, Brophy JC, Bitnun A, Samson LM (2012). A national review of vertical HIV transmission. AIDS.

[7] Castilla J, Del Romero J, Hernando V, Marincovich B, García S, Rodríguez C (2005). Effectiveness of highly active antiretroviral therapy in reducing heterosexual transmission of HIV. J Acquir Immune Defic Syndr.

[8] Weber S, Waldura JF, Cohan D (2013). Safer conception options for HIV serodifferent couples in the United States: the experience of the National Perinatal HIV Hotline and Clinicians’ Network. J Acquir Immune Defic Syndr.

[9] UNAIDS (2014). Post-2015: a global movement for HIV and sexual and reproductive health and rights.

[10] World Health Organization (2002). Defining sexual health. Report of a Technical Consultation on Sexual Health.

[11] Salmander Trust (2014). Building a safe house on firm ground: key findings from a global values and preferences survey regarding the sexual and reproductive health and human rights of women living with HIV.

[12] Gruskin S, Ferguson L (2009). Government regulation of sex and sexuality: in their own words. Reprod Health Matters.

[13] Kennedy CE, Spaulding AB, Brickley DB, Almers L, Mirjahangir J, Packel L (2010). Linking sexual and reproductive health and HIV interventions: a systematic review. J Int AIDS Soc.

[14] Gay J, Hardee K, Croce-Galis M, Hall C (2011). What works to meet the sexual and reproductive health needs of women living with HIV/AIDS. J Int AIDS Soc.

[15] Shapiro K, Ray S (2007). Sexual health for people living with HIV. Reprod Health Matters.

[16] Greene S (M)othering With HIV: Reconstructing & Resisting Health & Social Surveillance.

[17] Canadian HIV/AIDS Legal Network The criminalization of HIV non-disclosure in Canada and internationally.

[18] Montaner JS (2011). Treatment as prevention – a double hat-trick. Lancet.

[19] The Global Network of People Living with HIV (GNP+) (2010). The Global Criminalization Scan Report 2010.

[20] UNAIDS (2014). 90-90-90: an ambitious treatment target to help end the AIDS epidemic.

[21] Bernard E, Bennett-Carlson R (2012). Criminalization of HIV non-disclosure, exposure and transmission: background and current landscape.

[22] Canadian AIDS Society (2011). Positive prevention position statement (March 2011).

[23] Global Network of People Living with HIV (GNP+) U (2011). Positive health, dignity and prevention: a policy framework.

[24] Cohen MS, Chen YQ, McCauley M, Gamble T, Hosseinipour MC, Kumarasamy N (2011). Prevention of HIV-1 infection with early antiretroviral therapy. N Engl J Med.

[25] Loutfy MR, Wu W, Letchumanan M, Bondy L, Antoniou T, Margolese S (2013). Systematic review of HIV transmission between heterosexual serodiscordant couples where the HIV-positive partner is fully suppressed on antiretroviral therapy. PLoS One.

[26] Rodger A BT, Cambiano V, Vernazza P, Estrada V, Van Lunzen J, Collins S (2014). HIV transmission risk through condomless sex if HIV+ partner on suppressive ART: PARTNER Study.

[27] Lourenço L, Lima VD, Heath K, Nosyk B, Gilbert M, Colley G (2014). Process monitoring of an HIV treatment as prevention program in British Columbia, Canada. J Acquir Immune Defic Syndr.

[28] World Health Organization (2015). Maximizing the treatment and prevention potential of antiretroviral drugs: early country experiences towards implementing a treat-all policy.

[29] Nosyk B, Montaner JS, Colley G, Lima VD, Chan K, Heath K (2014). The cascade of HIV care in British Columbia, Canada, 1996–2011: a population-based retrospective cohort study. Lancet Infect Dis.

[30] Gardner EM, McLees MP, Steiner JF, del Rio C, Burman WJ (2011). The spectrum of engagement in HIV care and its relevance to test-and-treat strategies for prevention of HIV infection. Clin Infect Dis.

[31] Loutfy M, Tyndall M, Baril J-G, Montaner JS, Kaul R, Hankins C (2014). Canadian consensus statement on HIV and its transmission in the context of criminal law. Can J Infect Dis Med Microbiol.

[32] World Health Organization (2004). Gender dimensions of HIV status disclosure to sexual partners: rates, barriers and outcomes. A Review Paper.

[33] Rouleau G, Côté J, Cara C (2012). Disclosure experience in a convenience sample of Quebec-born women living with HIV: a phenomenological study. BMC Women's Health.

[34] Obermeyer CM, Baijal P, Pegurri E (2011). Facilitating HIV disclosure across diverse settings: a review. Am J Public Health.

[35] Adam BD, Corriveau P, Elliott R, Globerman J, English K, Rourke S (2015). HIV disclosure as practice and public policy. Crit Public Health.

[36] Mahajan AP, Sayles JN, Patel VA, Remien RH, Ortiz D, Szekeres G (2008). Stigma in the HIV/AIDS epidemic: a review of the literature and recommendations for the way forward. AIDS.

[37] Lowther K, Selman L, Harding R, Higginson IJ (2014). Experience of persistent psychological symptoms and perceived stigma among people with HIV on antiretroviral therapy (ART): a systematic review. Int J Nurs Stud.

[38] BC Centre for Excellence in HIV/AIDS (2013). STOP HIV/AIDS: seek and treat for optimal prevention of HIV/AIDS.

[39] BC Centre for Excellence in HIV/AIDS (2013). It's different now.

[40] Public Health Agency of Canada (2012). HIV and AIDS in Canada: Surveillance Report to December 31, 2012.

[41] De Reus LA, Few AL, Blume LB, Bengtson VL, Acock AC, Allen KR, Dilworth-Anderson P, Klein DM (2005). Multicultural and critical race feminisms: theorizing families in the third wave. Sourcebook of Family Theory and Research.

[42] Raphael D (2009). Social determinants of health: Canadian Perspectives.

[43] Benoit C, Shumka L (2007). Gendering the population health perspective: fundamental determinants of women's health. Final Report Prepared for the Women's Health Research Network.

[44] Carter A, Greene S, Nicholson V, O'Brien N, Sanchez M, Pokomandy AD (2015). Breaking the glass ceiling: increasing the meaningful involvement of women living with HIV/AIDS (MIWA) in the design and delivery of HIV/AIDS services. Health Care Women Int.

[45] Kaida A, Carter A, Lemay J, O'Brien N, Greene S, Nicholson V (2014). Hiring, training, and supporting peer researchers: operationalizing community-based research principles within epidemiological studies by, with, and for women living with HIV.

[46] Abelsohn K, Benoit AC, Conway T, Cioppa L, Smith S, Kwaramba G “Hear(ing) new voices”: peer reflections from community based survey development with women living with HIV. Prog Community Health Partnersh.

[47] Meston C, Trapnell P (2005). Development and validation of a five-factor sexual satisfaction and distress scale for women: the Sexual Satisfaction Scale for Women (SSS-W). J Sex Med.

[48] Carter A, Sereda P, Nohpal A, Colley G, Proulx-Boucher K, Thomas-Pavanel J (2015). Validating self-report of undetectable viral load against laboratory plasma viral load measures in a multi-site cohort of women living with HIV in British Columbia.

[49] Revicki DA, Sorensen S, Wu AW (1998). Reliability and validity of physical and mental health summary scores from the Medical Outcomes Study HIV Health Survey. Med Care.

[50] Wu AW (1999). MOS-HIV health survey users manual.

[51] Zhang W, O'Brien N, Forrest JI, Salters KA, Patterson TL, Montaner JS (2012). Validating a shortened depression scale (10 item CES-D) among HIV-positive people in British Columbia, Canada. PLoS One.

[52] Radloff LS (1977). The CES-D scale a self-report depression scale for research in the general population. Appl Psychol Meas.

[53] Wright K, Naar-King S, Lam P, Templin T, Frey M (2007). Stigma scale revised: reliability and validity of a brief measure of stigma for HIV+ youth. J Adolesc Health.

[54] Berger BE, Ferrans CE, Lashley FR (2001). Measuring stigma in people with HIV: psychometric assessment of the HIV stigma scale. Res Nurs Health.

[55] Akaike H (1974). A new look at the statistical model identification. IEEE Trans Automat Control.

[56] Wilson TE, Jean-Louis G, Schwartz R, Golub ET, Cohen MH, Maki P (2010). HIV infection and women's sexual functioning. J Acquir Immune Defic Syndr (1999).

[57] Ciccarone DH, Kanouse DE, Collins RL, Miu A, Chen JL, Morton SC (2003). Sex without disclosure of positive HIV serostatus in a US probability sample of persons receiving medical care for HIV infection. Am J Public Health.

[58] Bogart LM, Collins RL, Kanouse DE, Cunningham W, Beckman R, Golinelli D (2006). Patterns and correlates of deliberate abstinence among men and women with HIV/AIDS. Am J Public Health.

[59] Siegel K, Schrimshaw EW (2003). Reasons for the adoption of celibacy among older men and women living with HIV/AIDS. J Sex Res.

[60] Shahhosseini Z, Gardeshi ZH, Pourasghar M, Salehi F (2014). A review of affecting factors on sexual satisfaction in women. Mater Sociomed.

[61] Greene S, Symington A, Ion A, Carvalhal A, Loutfy M (2014). Judging mothers: criminalization's creep into the health and social care of HIV-positive mothers. Poster 213.

[62] Leigh BC, Temple MT, Trocki KF (1993). The sexual behavior of US adults: results from a national survey. Am J Public Health.

[63] Siegel K, Schrimshaw EW, Lekas H-M (2006). Diminished sexual activity, interest, and feelings of attractiveness among HIV-infected women in two eras of the AIDS epidemic. Arch Sex Behav.

[64] Sow K, Desclaux A (2011). Stratégies féminines face au risque de transmission sexuelle du VIH au temps des antirétroviraux. Les femmes à l'épreuve du VIH au Sud.

[65] Logie CH, James L, Tharao W, Loutfy MR (2011). HIV, gender, race, sexual orientation, and sex work: a qualitative study of intersectional stigma experienced by HIV-positive women in Ontario, Canada. PLoS Med.

[66] Carr RL, Gramling LF (2004). Stigma: a health barrier for women with HIV/AIDS. J Assoc Nurses AIDS Care.

[67] O'Byrne P, Willmore J, Bryan A, Friedman DS, Hendriks A, Horvath C (2013). Nondisclosure prosecutions and population health outcomes: examining HIV testing, HIV diagnoses, and the attitudes of men who have sex with men following nondisclosure prosecution media releases in Ottawa, Canada. BMC Public Health.

[68] Adam B, Elliott R, Corriveau P, English K (2014). Impacts of criminalization on everyday lives of people living with HIV in Canada. Sex Res Soc Policy J.

[69] Mykhalovskiy E, Betteridge G, McLay D (2010). HIV non-disclosure and the criminal law: establishing policy options for Ontario. [Section 4: Social research on the use of the criminal law to address HIV non-disclosure in Ontario: findings from a qualitative research study].

[70] Mykhalovskiy E (2011). The problem of “significant risk”: exploring the public health impact of criminalizing HIV non-disclosure. Soc Sci Med.

[71] Keegan A, Lambert S, Petrak J (2005). Sex and relationships for HIV-positive women since HAART: a qualitative study. Aids Patient Care STDS.

[72] Bott S, Obermeyer CM (2013). The social and gender context of HIV disclosure in sub-Saharan Africa: a review of policies and practices. SAHARA J.

[73] UNAIDS Positive health, dignity and prevention.

[74] St-Pierre-Gagné S, Otis J, Massie L, Kéïta DB, Yattassaye A, Dème R (2014). Emotional and sexual well-being of women living with HIV in Quebec: Cultural adaptation and formative evaluation of the program 〈〈PLURIELLES〉〉.

[75] Csete J (2005). ‘Vectors, vessels and victims’: HIV/AIDS and women's human rights in Canada.

# Sub article: The time has come to make cervical cancer prevention an essential part of comprehensive sexual and reproductive health services for HIV-positive women in low-income countries

**DOI:** 10.7448/IAS.18.6.20282

**Published:** 2015-12-01

**Authors:** Megan J Huchko, May Maloba, Miriam Nakalembe, Craig R Cohen

**Affiliations:** 1 Department of Obstetrics, Gynecology and Reproductive Sciences, University of California San Francisco, San Francisco, CA, USA; 2 Family AIDS Care and Education Services, Kenya Medical Research Institute, Kisumu, Kenya; 3 Department of Obstetrics and Gynaecology and Infectious Disease Institute, Makerere University, Kampala, Uganda

**Keywords:** cervical cancer prevention, HIV, integration, low- and middle-income countries

## Abstract

**Introduction:**

HIV and cervical cancer are intersecting epidemics that disproportionately affect one of the most vulnerable populations in the world: women in low- and middle-income countries (LMICs). Historically, the disparity in cervical cancer risk for women in LMICs has been due to the lack of organized screening and prevention programmes. In recent years, this risk has been augmented by the severity of the HIV epidemic in LMICs. HIV-positive women are at increased risk for developing cervical precancer and cancer, and while the introduction of antiretroviral therapy has dramatically improved life expectancies among HIV-positive women it has not been shown to improve cancer-related outcomes. Therefore, an increasing number of HIV-positive women are living in LMICs with limited or no access to cervical cancer screening programmes. In this commentary, we describe the gaps in cervical cancer prevention, the state of evidence for integrating cervical cancer prevention into HIV programmes and future directions for programme implementation and research.

**Discussion:**

Despite the biologic, behavioural and demographic overlap between HIV and cervical cancer, cervical cancer prevention has for the most part been left out of sexual and reproductive health (SRH) services for HIV-positive women. Lower cost primary and secondary prevention strategies for cervical cancer are becoming more widely available in LMICs, with increasing evidence for their efficacy and cost-effectiveness. Going forward, cervical cancer prevention must be considered a part of the essential package of SRH services for HIV-positive women. Effective cervical cancer prevention programmes will require a coordinated response from international policymakers and funders, national governments and community leaders. Leveraging the improvements in healthcare infrastructure created by the response to the global HIV epidemic through integration of services may be an effective way to make an impact to prevent cervical cancer among HIV-positive women, but more work remains to determine optimal approaches.

**Conclusions:**

Cervical cancer prevention is an essential part of comprehensive HIV care. In order to ensure maximal impact and cost-effectiveness, implementation strategies for screening programmes must be adapted and rigorously evaluated through a framework that includes equal participation with policymakers, programme planners and key stakeholders in the target communities.

## Introduction

The combined threat that cervical cancer and HIV present to women's quality of life, reproductive capacity and overall mortality highlights a glaring inequality in global women's health. The enormous international disparity in the incidence of and survival from cervical cancer has historically aligned most closely with country income [1]. Nearly 85% of cases and 87% of deaths occur in less developed regions, where several factors conspire to make cervical cancer a leading cause of cancer and cancer-related mortality [2]. Inadequate healthcare and public health infrastructure, competing health priorities and persistent poverty prevent large-scale cervical cancer prevention programmes from gaining traction, resulting in only a small minority of the population being screened [3]. Rates of cervical cancer in less developed countries are similar to those seen in the United States prior to the introduction of widespread screening [4].

In the last 20 years, the increased burden of cervical cancer has been intensified by the contribution of HIV to cervical precancer and cancer. Global maps showing country-specific HIV prevalence match the global maps of cervical cancer incidence and mortality. For example, the incidence of cervical cancer is 42.7 per 100,000 women in Eastern Africa, a high HIV-prevalence region with low screening coverage, compared to 30.6/100,000 in Middle Africa (moderate HIV prevalence, low screening) and 6.6/100,000 in Northern Africa (low HIV prevalence, moderate screening coverage) [5]. The high rate of HIV infection in many low- and middle-income countries (LMICs) has potentiated the already increased risk for cervical cancer for women living in these countries. The decrease in cellular immunity caused by HIV increases the risk for new and persistent human papillomavirus (HPV) infections – the primary cause of cervical cancers and precancerous cervical lesions – and contributes to an accelerated incidence and progression of cervical neoplasia [6, 7].

Increased availability of HIV care and treatment, combined with greater coverage of antiretroviral therapy (ART) in recent years, has been lifesaving for entire populations of HIV-positive women. In contrast to other AIDS-related malignancies, which show improvement with ART, the positive effect of ART on cervical cancer outcomes is not clear [8–11]. Conversely, researchers have shown that the risk of anal cancer, another HPV-related malignancy, actually increases after ART use, making it plausible that the biologic risk for cervical cancer may increase [12]. Regardless of the direct biologic effect of ART on cervical cancer risk, in the many LMICs that have addressed their high HIV prevalence through improved HIV testing and access to treatment, there is a significant increase in the number of HIV-positive women living longer with excess cervical cancer risk [13]. This makes the implementation of effective screening programmes an urgent public health priority, especially for the HIV-positive women who are most vulnerable to the disease.

There is a precedent of successful partnerships between international donors and local governments to strengthen healthcare infrastructure and build local capacity in ways that helped to stem the HIV epidemic. Many government health systems have successfully leveraged these gains in the healthcare system and numbers of trained healthcare workers to address other healthcare needs such as tuberculosis, malaria, family planning, maternal health and other non-communicable diseases (NCDs) [14–16]. As evidence for the efficacy and cost-effectiveness of integrating these other health services increases, there has been an increase in donor funding and policy commitment to support integration. However, cervical cancer is routinely excluded from the definition of *sexual and reproductive health* (SRH) *services*, which often focus on family planning, prevention of maternal-to-child transmission (PMTCT) of HIV and sexually transmitted infection (STI) prevention [17–20]. While the World Health Organization (WHO) *2006 Guidelines on Sexual and Reproductive Health for Women Living with HIV* do include cervical cancer screening as a topic area, inclusion of cervical cancer prevention as part of essential services for HIV-positive women is not a focus of that document. Rather, the section on cervical cancer concludes with recommendations for HIV-positive women to have the same access to cervical cancer screening as HIV-negative women [21]. As research into integration of reproductive health and HIV services evolved, more recent documents that focus on provision of comprehensive care for people living with HIV in LMICs include recommendations on how to integrate family planning, STI prevention and PMTCT, while cervical cancer is not mentioned [22, 23]. The global health community is failing women in a crucial way: it has neglected prevention, screening and treatment for cervical cancer among the highest risk population, HIV-positive women in LMICs. In this commentary, we describe the current policy and evidence around strategies for implementing cervical cancer into HIV care and recommend future research and policy directions to ensure that cervical cancer prevention is included as part of essential SRH services for HIV-positive women.

## Discussion

There are several reasons for the exclusion of cervical cancer as part of comprehensive care for women living with HIV. Primarily, the majority of the world's HIV-positive women live in countries where there is no access to cervical cancer prevention for anyone, regardless of their serostatus. One of the effects of this lack of screening infrastructure is an absence of cancer registries in most LMICs. Without accurate estimates of the number of cases each year and the impact of HIV on the incidence and prevalence of cervical cancer, it is impossible to set targets and track progress in addressing this issue. Another reason for the exclusion of cervical cancer from SRH services offered to HIV-positive women is that, despite being caused by an infectious agent, it is often conceptualized as an NCD, rather than a component of SRH. Instead of receiving increased attention by having a home in two different content areas, this dual identity has actually led to less focus and attention for cervical cancer prevention, which is often seen as not fully belonging in either category. As the immediate and pressing needs of the HIV epidemic have begun to abate, there is an opportunity to use the lessons from both NCD and SRH management to address cervical cancer prevention in a way that best fits the unique characteristics of the disease.

Cervical cancer prevention fits into an NCD paradigm of integrating preventative care into existing clinics through periodic, evidence-based screening, with treatment of early or preclinical disease. Importantly, though, because of the counselling, outreach, screening techniques and fertility implications for treatment of invasive disease, cervical cancer prevention has a natural place in SRH services. Providers who are more comfortable talking to women about their reproductive health and family planning, and who can ably counsel women and perform pelvic exams, may be better suited to perform cervical cancer screening [24]. A successful cervical cancer prevention programme should include elements from NCD prevention strategies (disease awareness, coupled with periodic, universal screening and access to risk-reduction interventions) when providing services under the paradigm of reproductive health.

Another key reason for the exclusion of cervical cancer from primary healthcare in LMICs, and more recently from comprehensive HIV care, was the lack of feasible and affordable prevention strategies. We now have a wide range of low-cost and effective primary and secondary prevention options that can be operationalized in LMICs, making dramatic global reductions in cervical cancer incidence a realistic goal within a generation. HPV vaccination is the most successful and cost-effective strategy for cervical cancer prevention, especially in high HIV-prevalence areas [25–27]. The WHO has prequalified two HPV vaccines that could dramatically reduce cervical cancer deaths in LMICs if vaccination coverage can be scaled up [28]. GAVI, the Global Vaccine Alliance, is supporting initiatives to provide vaccines in selected LMICs, and pilot delivery programmes are ongoing [29]. The vaccination of adolescent girls also provides an opportunity to provide them with other reproductive health services and health education (including family planning and menstrual hygiene); it would provide primary prevention of HPV and cervical cancer prior to sexual exposure to HIV. Ensuring that adolescent girls have the opportunity to receive a vaccine that protects them from the morbidity and mortality related to cervical cancer should be a key global health priority.

Conventional screening methods, using Pap smears and biopsies, require infrastructure and clinical expertise and are hard to scale up in LMICs. However simpler, cheaper screening techniques, such as visual inspection with acetic acid (VIA) and HPV DNA testing, hold great promise and are undergoing widespread evaluation [30, 31]. The WHO Global Action Plan on NCDs describes screening with VIA as a “best buy,” meaning that it is both highly cost effective (i.e. it costs less than the per capita gross domestic product to avert one disability adjusted life-year) and it is feasible to implement in settings with constrained health systems [32]. There are promising results from large trials, suggesting that VIA can reduce cervical cancer incidence by 25 to 30% [33], with similar performance characteristics among HIV-positive women compared to HIV-negative women [34]. Although screening with HPV is more expensive than with VIA, a study by Goldie *et al*. [35] in five LMICs found that HPV screening is very cost-effective, and a single test at age 35 years reduces lifetime cancer risk by 25 to 36%. This finding has been supported in models of HPV screening among HIV-positive women [36]. Ongoing and completed studies are looking at novel strategies to maximize uptake of HPV screening, including self-collection and community health campaign models, in low-resource/high HIV-prevalence settings [37, 38]. The WHO has recognized and summarized the evidence for low-cost cervical cancer prevention strategies in their *2013 Comprehensive Cervical Cancer Prevention and Control Manual* [39], which includes recommendations for screening strategies for HIV-positive women.

One strategy for ensuring that HIV-positive women access cervical cancer screening and prevention is through service integration. Integrating care for HIV, sexual health, reproductive health and maternal health has been shown to improve uptake of services, reduce HIV-related stigma and improve the quality of care received by women [40, 41]. Although there are many definitions of *integration*, the model that is most feasible for cervical cancer and HIV care is integration of cervical cancer services into existing HIV-care programmes, given the lack of standalone cervical cancer prevention clinics and periodicity of screening. There is growing evidence for the feasibility of integrating cervical cancer prevention into HIV services using low-cost screening strategies coupled with treatment for precancerous lesions [42–45]. Furthermore, integrating cervical cancer prevention services into HIV primary-care facilities, rather than referring women to a separate family planning or reproductive health facility, provides an opportunity to include and educate male partners, which may be particularly important in areas where men have control over healthcare decisions [46, 47].

However, integration may not be feasible or successful in all settings. While integration holds the promise of leveraging stronger health systems to improve access to and uptake of secondary services in higher risk populations through a decrease in the visit burden and loss to follow-up, several studies in sub-Saharan Africa have shown significant weaknesses in models of various health services integrated into HIV care. These include limited interest among the general population in receiving care through integrated models [41], concerns about disclosure and resultant stigma in general outpatient settings [40], lack of clear policies, unacceptable clinical load on the staff, longer wait times and concerns about quality of care [48].

While the promise of integration has not been borne out in every setting, this does not mean that it should be discarded for the next big idea in service delivery. One randomized study of integrated HIV and antenatal services showed high rates of attrition in both arms, suggesting that there are structural barriers to uptake that lie outside of the care model [49]. This finding, along with the difficulties experienced in different settings, speaks to the need for community-driven, context-specific adaptation of the evidence-proven interventions for cervical cancer prevention, specifically VIA, HPV testing and “see and treat” strategies. While the efficacy and effectiveness of these low-cost strategies have been clearly shown in large, well-conducted trials, there are few implementation studies done in partnership with target communities to adapt and iteratively evaluate the effectiveness of the resulting intervention and implementation strategy. Implementation and dissemination science, or “the scientific study of methods to promote the systematic uptake of research findings and other evidence-based practices into routine practice, and hence, to improve the quality and effectiveness of health services and care,” provides tools to bridge the gap between scientific evidence and public health practices and policy. In addition to the standard clinical effectiveness outcomes, implementation studies evaluate a combination of quantitative, qualitative and process measures to evaluate the feasibility and sustainability of the implementation, essentially exploring and explaining the individual, interpersonal, community and policy-level factors necessary for the success of evidence-based interventions. The above-cited studies of self-collected HPV in Uganda and Kenya are examples of using implementation science research to address the gap between evidence-based cervical cancer prevention, policy and uptake.

## Conclusions

HIV and cervical cancer are intersecting epidemics that disproportionately affect one of the most vulnerable populations in the world: women in LMICs. Despite the biologic, behavioural and demographic overlap, cervical cancer prevention has for the most part been left out of SRH services for HIV-positive women. Similar to the coordinated and multilateral response to the HIV epidemic, an effective programme for cervical cancer prevention among HIV-positive women needs international, national and community leadership for a broad-based and sustainable response. International guidelines for HIV care in LMICs must include a mandate to provide cervical cancer prevention as part of comprehensive SRH care. Funding agencies and local governments must then consider this a key component of HIV care and provide the funding, training, support supervision and accountability necessary to ensure maximal coverage of services. Implementation studies done in partnership with local governments, key stakeholders and programmes providing HIV care will facilitate cervical cancer prevention strategies that are not only included as part of the essential package of services, but are provided in a context-specific way. Cervical cancer prevention has the potential to be effective, sustainable and cost-effective. A crucial part of the implementation strategy will be developing a monitoring and evaluation programme to measure the coverage and quality of cervical cancer prevention services provided as part of comprehensive SRH services for HIV-positive women.

The climate is right for a coordinated response to the dual threat posed by HIV and cervical cancer in LMICs: low-cost strategies, improved health infrastructure and engagement in the healthcare system by a high-risk population. The ability to impact this long-standing global health disparity is well within our reach.

## References

[1] Farmer P, Frenk J, Knaul FM, Shulman LN, Alleyne G, Armstrong L (2010). Expansion of cancer care and control in countries of low and middle income: a call to action. Lancet.

[2] Ferlay J, Shin H, Bray F, Forman D, Mathers C, Parkin DM (2010). GLOBOCAN 2008 v2.0. Cancer Incidence and Mortality Worldwide: IARC CancerBase No. 10.

[3] Adefuye PO, Broutet NJ, de Sanjose S, Denny LA (2013). Trials and projects on cervical cancer and human papillomavirus prevention in sub-Saharan Africa. Vaccine.

[4] Gustafsson L, Ponten J, Bergstrom R, Adami HO (1997). International incidence rates of invasive cervical cancer before cytological screening. Int J Cancer.

[5] Ferlay J, Soerjomataram I, Dikshit R, Eser S, Mathers C, Rebelo M (2015). Cancer incidence and mortality worldwide: sources, methods and major patterns in GLOBOCAN 2012. Int J Cancer.

[6] Ezechi OC, Ostergren PO, Nwaokorie FO, Ujah IA, Odberg Pettersson K (2014). The burden, distribution and risk factors for cervical oncogenic human papilloma virus infection in HIV positive Nigerian women. Virol J.

[7] Palefsky J (2006). Biology of HPV in HIV infection. Adv Dent Res.

[8] Blitz S, Baxter J, Raboud J, Walmsley S, Rachlis A, Smaill F (2013). Evaluation of HIV and highly active antiretroviral therapy on the natural history of human papillomavirus infection and cervical cytopathologic findings in HIV-positive and high-risk HIV-negative women. J Infect Dis.

[9] Bratcher LF, Sahasrabuddhe VV (2010). The impact of antiretroviral therapy on HPV and cervical intraepithelial neoplasia: current evidence and directions for future research. Infect Agents Cancer.

[10] Patrelli TS, Gizzo S, Peri F, Franchi L, Volpi L, Esposito F (2013). Impact of highly active antiretroviral therapy on the natural history of cervical precancerous lesions: a 17-year institutional longitudinal cohort study. Reprod Sci.

[11] Dryden-Peterson S, Medhin H, Kebabonye-Pusoentsi M, Seage GR, Suneja G, Kayembe MK (2015). Cancer incidence following expansion of HIV treatment in Botswana. PLoS One.

[12] Piketty C, Selinger-Leneman H, Grabar S, Duvivier C, Bonmarchand M, Abramowitz L (2008). Marked increase in the incidence of invasive anal cancer among HIV-infected patients despite treatment with combination antiretroviral therapy. AIDS.

[13] Franceschi S, Jaffe H (2007). Cervical cancer screening of women living with HIV infection: a must in the era of antiretroviral therapy. Clin Infect Dis.

[14] FHI360 integration of HIV and noncommunicable disease care.

[15] Haraka F, Glass TR, Sikalengo G, Gamell A, Ntamatungiro A, Hatz C (2015). A bundle of services increased ascertainment of tuberculosis among HIV-infected individuals enrolled in a HIV cohort in rural sub-Saharan Africa. PLoS One.

[16] Suthar AB, Rutherford GW, Horvath T, Doherty MC, Negussie EK (2014). Improving antiretroviral therapy scale-up and effectiveness through service integration and decentralization. AIDS.

[17] Dickinson C, Attawell K, Druce N (2009). Progress on scaling up integrated services for sexual and reproductive health and HIV. Bull World Health Organ.

[18] Dhana A, Luchters S, Moore L, Lafort Y, Roy A, Scorgie F (2014). Systematic review of facility-based sexual and reproductive health services for female sex workers in Africa. Global Health.

[19] Hope R, Kendall T, Langer A, Barnighausen T (2014). Health systems integration of sexual and reproductive health and HIV services in sub-Saharan Africa: a scoping study. J Acquir Immune Defic Syndr.

[20] IPPF, UNFPA, WHO, UNAIDS, GNP+, ICW (2009). Rapid assessment tool for sexual and reproductive health and HIV linkages [Internet].

[21] World Health Organization (2006). Sexual and reproductive health of women living with HIV/AIDS: guidelines on care, treatment and support for women living with HIV/AIDS and their children in resource-constrained settings.

[22] World Health Organization (2008). Operations manual for delivery of HIV prevention, care and treatment at primary health centers in high-prevalence, resource-constrained settings.

[23] World Health Organization (2010). Priority interventions – HIV/AIDS prevention, treatment and care in the health sector.

[24] Rosser JI, Hamisi S, Njoroge B, Huchko MJ (2015). Barriers to cervical cancer screening in rural Kenya: perspectives from a provider survey. J Community Health.

[25] Seto K, Marra F, Raymakers A, Marra CA (2012). The cost effectiveness of human papillomavirus vaccines: a systematic review. Drugs.

[26] Goldie SJ, O'Shea M, Campos NG, Diaz M, Sweet S, Kim SY (2008). Health and economic outcomes of HPV 16,18 vaccination in 72 GAVI-eligible countries. Vaccine.

[27] Joura EA, Garland SM, Paavonen J, Ferris DG, Perez G, Ault KA (2012). Effect of the human papillomavirus (HPV) quadrivalent vaccine in a subgroup of women with cervical and vulvar disease: retrospective pooled analysis of trial data. BMJ.

[28] Parry J (2007). Vaccinating against cervical cancer. Bull World Health Organ.

[29] GAVI Alliance Human papillomavirus vaccine support [Internet].

[30] Sankaranarayanan R, Esmy PO, Rajkumar R, Muwonge R, Swaminathan R, Shanthakumari S (2007). Effect of visual screening on cervical cancer incidence and mortality in Tamil Nadu, India: a cluster-randomised trial. Lancet.

[31] Sankaranarayanan R, Nene BM, Dinshaw K, Rajkumar R, Shastri S, Wesley R (2003). Early detection of cervical cancer with visual inspection methods: a summary of completed and on-going studies in India. Salud Publica Mex.

[32] World Health Organization Choosing interventions that are cost effective: cost-effectiveness thresholds [Internet].

[33] Sankaranarayanan R, Nene BM, Dinshaw KA, Mahe C, Jayant K, Shastri SS (2005). A cluster randomized controlled trial of visual, cytology and human papillomavirus screening for cancer of the cervix in rural India. Int J Cancer.

[34] Huchko MJ, Sneden J, Zakaras JM, Smith-McCune K, Sawaya G, Maloba M (2015). A randomized trial comparing the diagnostic accuracy of visual inspection with acetic acid to visual inspection with Lugol's iodine for cervical cancer screening in HIV-infected women. PLoS One.

[35] Goldie SJ, Gaffikin L, Goldhaber-Fiebert JD, Gordillo-Tobar A, Levin C, Mahe C (2005). Cost-effectiveness of cervical-cancer screening in five developing countries. N Eng J Med.

[36] Goldie SJ, Freedberg KA, Weinstein MC, Wright TC, Kuntz KM (2001). Cost effectiveness of human papillomavirus testing to augment cervical cancer screening in women infected with the human immunodeficiency virus. Am J Med.

[37] Moses E, Pedersen HN, Mitchell SM, Sekikubo M, Mwesigwa D, Singer J (2015). Uptake of community-based, self-collected HPV testing vs. visual inspection with acetic acid for cervical cancer screening in Kampala, Uganda: preliminary results of a randomised controlled trial. Trop Med Int Health.

[38] Evaluating a Community-Driven Cervical Cancer Prevention Strategy in Western Kenya https://clinicaltrials.gov/ct2/show/NCT02124252.

[39] WHO (2013). Comprehensive cervical cancer prevention and control: a healthier future for girls and women. WHO Guidance Note.

[40] Church K, Wringe A, Fakudze P, Kikuvi J, Simelane D, Mayhew SH (2013). Are integrated HIV services less stigmatizing than stand-alone models of care? A comparative case study from Swaziland. J Int AIDS Soc.

[41] Mak J, Birdthistle I, Church K, Friend-Du Preez N, Kivunaga J, Kikuvi J (2013). Need, demand and missed opportunities for integrated reproductive health-HIV care in Kenya and Swaziland: evidence from household surveys. AIDS.

[42] Ekong J, Kakande C, Mutabazi M, Kakande H, Castano F, Uhuru K (2013). Integration of cervical cancer screening using visual inspection with acetic acid and cryotherapy treatment into HIV/AIDS services in rural districts of Western Uganda.

[43] Mutyaba T, Mirembe F, Sandin S, Weiderpass E (2010). Evaluation of ‘see-see and treat’ strategy and role of HIV on cervical cancer prevention in Uganda. Reprod Health.

[44] Denny LA, Sankaranarayanan R, De Vuyst H, Kim JJ, Adefuye PO, Alemany L (2013). Recommendations for cervical cancer prevention in sub-Saharan Africa. Vaccine.

[45] Huchko MJ, Bukusi EA, Cohen CR (2011). Building capacity for cervical cancer screening in outpatient HIV clinics in the Nyanza province of western Kenya. Int J Gynaecol Obstet.

[46] Rosser JI, Zakaras JM, Hamisi S, Huchko MJ (2014). Men's knowledge and attitudes about cervical cancer screening in Kenya. BMC Womens Health.

[47] Mutyaba T, Mirembe F, Sandin S, Weiderpass E (2009). Male partner involvement in reducing loss to follow-up after cervical cancer screening in Uganda. Int J Gynaecol Obstet.

[48] Cooper D, Mantell JE, Moodley J, Mall S (2015). The HIV epidemic and sexual and reproductive health policy integration: views of South African policymakers. BMC Public Health.

[49] Washington S, Owuor K, Turan JM, Steinfeld RL, Onono M, Shade SB (2015). The effect of integration of HIV care and treatment into antenatal care clinics on mother-to-child HIV transmission and maternal outcomes in Nyanza, Kenya: results from the SHAIP cluster randomized controlled trial. J Acquir Immune Defic Syndr.

# Sub article: A discussion of key values to inform the design and delivery of services for HIV-affected women and couples attempting pregnancy in resource-constrained settings

**DOI:** 10.7448/IAS.18.6.20272

**Published:** 2015-12-01

**Authors:** Renee Heffron, Natasha Davies, Ian Cooke, Angela Kaida, Reid Mergler, Sheryl van der Poel, Craig R Cohen, Okeoma Mmeje

**Affiliations:** 1 Department of Global Health, University of Washington, Seattle, WA, USA;; 2 Department of Epidemiology, University of Washington, Seattle, WA, USA; 3 Wits Reproductive Health and HIV Institute, University of Witwatersrand, Johannesburg, South Africa; 4 Department of Human Metabolism, University of Sheffield, Sheffield, UK; 5 Faculty of Health Sciences, Simon Fraser University, Burnaby, Canada; 6 Albert Einstein College of Medicine, Bronx, NY, USA; 7 Department of Reproductive Health and Research Including the Human Reproduction Research Programme, World Health Organization, Geneva, Switzerland; 8 Department of Obstetrics, Gynecology & Reproductive Sciences, University of California San Francisco, San Francisco, CA, USA; 9 Department of Obstetrics & Gynecology, University of Michigan, Ann Arbor, MI, USA; 10 Department of Health Behavior and Health Education, University of Michigan, Ann Arbor, MI, USA

**Keywords:** HIV, pregnancy, values, reproductive health, fertility, women, couples

## Abstract

**Introduction:**

HIV-affected women and couples often desire children and many accept HIV risk in order to attempt pregnancy and satisfy goals for a family. Risk reduction strategies to mitigate sexual and perinatal HIV transmission include biomedical and behavioural approaches. Current efforts to integrate HIV and reproductive health services offer prime opportunities to incorporate strategies for HIV risk reduction during pregnancy attempts. Key client and provider values about services to optimize pregnancy in the context of HIV risk provide insights for the design and implementation of large-scale “safer conception” programmes.

**Discussion:**

Through our collective experience and discussions at a multi-disciplinary international World Health Organization–convened workshop to initiate the development of guidelines and an algorithm of care to support the delivery of services for HIV-affected women and couples attempting pregnancy, we identified four values that are key to the implementation of these programmes: (1) understanding fertility care and an ability to identify potential fertility problems; (2) providing equity of access to resources enabling informed decision-making about reproductive choices; (3) creating enabling environments that reduce stigma associated with HIV and infertility; and (4) creating enabling environments that encourage disclosure of HIV status and fertility status to partners. Based on these values, recommendations for programmes serving HIV-affected women and couples attempting pregnancy include the following: incorporation of comprehensive reproductive health counselling; training to support the transfer and exchange of knowledge between providers and clients; care environments that reduce the stigma of childbearing among HIV-affected women and couples; support for safe and voluntary disclosure of HIV and fertility status; and increased efforts to engage men in reproductive decision-making at times that align with women's desires.

**Conclusions:**

Programmes, policies and guidelines that integrate HIV treatment and prevention, sexual and reproductive health and fertility care services in a manner responsive to user values and preferences offer opportunities to maximize demand for and use of these services. For HIV-affected women and couples attempting pregnancy, the provision of comprehensive services using available tools – and the development of new tools that are adaptable to many settings and follow consensus recommendations – is a public health imperative. The impetus now is to design and deliver value-driven inclusive programming to achieve the greatest coverage and impact to reduce HIV transmission during pregnancy attempts.

## Introduction

Attempting pregnancy to achieve goals for family size is a basic reproductive right for all individuals, including HIV-affected women and couples, whether they be HIV-infected women with partners of known and unknown HIV status, HIV-serodiscordant couples, or HIV-uninfected women with a high risk for HIV acquisition [1]. HIV-affected women and couples face a dilemma when attempting pregnancy, a period associated with heightened risk of HIV transmission. Many accept increased HIV risk in order to satisfy their fertility goals [2]. Pregnancy rates among HIV-affected women and couples have been documented at 10 to 15% per year, including substantial proportions with partners of unknown or HIV-serodiscordant status and important proportions that are unintended [3]. Antiretroviral use by women is associated with improved health and hopefulness for the future, contributing to desires to have more children while seeking care to minimize HIV risk during pregnancy attempts [4–9]. Thus, there is a great clinical need and a public health imperative to provide services for HIV-affected women and couples desiring pregnancy to maximize the potential for a healthy pregnancy while minimizing sexual and perinatal HIV transmission risks.

Biomedical and behavioural strategies are available to support pregnancy attainment while minimizing HIV risk (i.e. “safer conception” strategies) and international guidelines to optimize pregnancy and HIV outcomes are under development [10]. For HIV-affected women and couples with normal fertility, evidence-based strategies include antiretroviral therapy use by the HIV-infected partner, the use of pre-exposure prophylaxis (PrEP) by the HIV-uninfected partner, limiting condomless sex to periods of peak fertility, treatment of sexually transmitted infections, voluntary medical male circumcision and manual insemination (for serodiscordant couples with an HIV-uninfected male partner) [11–15]. Fertility care services, including fertility screening and management, offer additional options when fertility may be compromised. The availability of multiple strategies offers choice and the possibility of combining strategies to maximize harm reduction.

The integration of HIV and reproductive health care services provides a natural opportunity to incorporate pregnancy planning and fertility management for HIV-affected women and couples attempting pregnancy [16–18]. Models that include comprehensive reproductive, sexual, maternal and child health services – and can accommodate individuals at any stage in their reproductive life – have the potential to ensure more equitable service provision and reduce missed opportunities to prevent HIV transmission, unintended pregnancy and poor pregnancy outcomes [19]. Within this integration, opportunities also arise to create programmes that encourage the engagement of men as supporters of HIV-infected women in reproductive health decision-making and as partners in health optimization prior to and during pregnancy. Thus, recommendations for the design and delivery of services for HIV-affected women and couples attempting pregnancy can be contextualized within the premise of integrated systems, generating programmes that minimize burden, are cost-effective and place HIV prevention and reproductive health care within a holistic and rights-based framework.

This commentary, developed through collaboration by HIV prevention and fertility care experts, discusses key values of HIV-affected women and couples seeking services to reduce HIV risk during pregnancy attempts as well as those of healthcare providers encountering opportunities to initiate discussion about fertility goals and provide pre-pregnancy care. A greater understanding of these values is essential for maximizing the impact of safer conception programmes and services. HIV-affected women and couples can also meet their family goals through adoption, donor gamete or surrogacy, but values related to these methods are beyond the scope of this discussion.

## Discussion

In December 2014, the WHO convened a meeting to initiate the development of guidelines and an algorithm of care to support the delivery of services for HIV-affected women and couples attempting pregnancy. During discussions, the authors identified four key values to incorporate into the development of programmes for HIV-affected women and couples. The attendees were experts in HIV prevention and treatment as well as reproductive medicine whose experiences and knowledge about each other's fields was initially limited. Through structured debate among the attendees, four key values emerged as being central to programmatic recommendations: (1) understanding fertility care and the ability to identify potential fertility problems; (2) providing equity of access to resources enabling informed decision-making about reproductive choices; (3) creating enabling environments that reduce stigma associated with HIV and infertility; and (4) creating enabling environments that encourage disclosure of HIV status and fertility status to partners.

### Understanding fertility care and the ability to identify potential fertility problems

Fertility awareness or confidence in the ability to become pregnant is often questioned by individuals before pregnancy occurs. HIV-affected women and couples may unknowingly have fertility problems and would value provider counselling on how to evaluate fertility, diagnose infertility and, if needed, receive fertility services before attempting pregnancy in order to minimize condomless sex and thus the potential for HIV transmission. The prevalence of involuntary childlessness due to infertility has been estimated at one in every four couples in developing countries [20]. Thus, providers working with HIV-affected women and couples with fertility aspirations value information that allows: 1) a recommendation to presumed fertile couples to engage in condomless sex timed to peak fertility during the menstrual cycle, or 2) access to basic fertility evaluation and affordable referral options should infertility be suspected (Figure 1).

**Figure 1:** Key values and service delivery guidance for programmes to reduce HIV risk during pregnancy attempts (i.e. safer conception services). The identified key values of HIV-affected women and couples were drawn from client and provider values and preferences and provide guidance for the integration of services to reduce HIV risk during pregnancy attempts. For example, the key value of understanding fertility care and having the ability to identify potential fertility problems is incorporated into guidance that comprehensive reproductive health counselling should incorporate discussion of fertility goals, including simple fertility screening and care. The identification of additional values and service delivery guidance would be warranted through further collaborative discussions.

Simple screening questionnaires that assess reproductive and lifestyle factors have been developed for individuals to self-assess their fertility potential and to facilitate provider-initiated discussions of reproductive desires and likelihood of normal fecundity [8]. Providers can conduct a basic fertility evaluation to understand history of past pregnancy losses, terminations and complications; menstrual cycle abnormalities for the woman; a semen analysis for the man; and assessment of individual and couples’ lifestyle, environmental exposures, STIs and sexual dysfunction that may alter fertility fitness [21]. These provider competencies must be complemented by offering opportunities for HIV-affected women and their partners to ask questions and become knowledgeable about their reproductive potential. Basic fertility interventions, such as vaginal insemination, could be offered in addition to an option of natural conception under the premise of viral suppression with antiretrovirals and/or the use of PrEP. However, if a desired pregnancy has not occurred within 12 months of regular condomless sex without contraception – or after six months when HIV risk is high – then HIV-affected women and couples are likely to be at significant risk of extended HIV exposure with a reduced chance of pregnancy, and affordable advanced diagnostic techniques and care services should be offered.

An infertility diagnosis can be devastating, especially for HIV-affected women and couples who are already struggling with HIV-related stigma. Providers, in anticipation of this additional distress from fertility problems, must be armed with appropriate knowledge, tools and counselling skills to provide basic fertility care, support and referral to infertility services for advanced diagnosis and management as well as HIV prevention options. To respect the reproductive rights of HIV-affected women and couples to achieve fertility goals, HIV and fertility service providers need to collaborate to ensure comprehensive, coordinated care.

### Providing equity of access to resources enabling informed decision-making about reproductive choices

HIV-affected women and couples value autonomy and the ability to make informed decisions about attempting pregnancy in the face of HIV risk and/or undiagnosed fertility problems. Current evidence suggests that women and men have low awareness of HIV risk reduction strategies they can use during pregnancy attempts, despite highly expressed interest in learning about them [2]. Knowledge about accessibility is an important corollary, because PrEP, fertility screening and basic and advanced assisted reproductive interventions are not universally available in resource-limited settings. In addition, women at risk of acquiring HIV who are considering PrEP require complete and balanced safety data about use during pregnancy, including recent data showing that PrEP does not significantly affect the risk of adverse maternal and infant outcomes [22].

Providers require the skills and confidence to provide comprehensive and non-judgmental guidance about suitable HIV risk reduction and fertility care options. Reproductive health and HIV providers must incorporate a broader exploration of reproductive goals into their established counselling on contraceptive uptake, antiretroviral use to prevent perinatal HIV transmission and consistent condom use. Despite general provider empathy with the social and cultural importance of HIV-infected women having children, knowledge and training on how to support women with this goal remains limited [23–25]. Additionally, many providers struggle with the tension between acknowledging the reproductive rights of HIV-affected women and couples and the clinical responsibility to protect partners and future children from HIV transmission and other pregnancy-related consequences [26]. The traditional emphasis of counselling on consistent condom use must shift, acknowledging the tremendous benefit of antiretrovirals (as treatment and PrEP) as HIV prevention strategies that enable safer condomless sex and support couples to attain their pregnancy goals.

Prevailing social and structural barriers may impact HIV-affected women and couples’ ability to utilize HIV and reproductive health services. For example, optimizing pre-pregnancy maternal health is important for pregnancy planning but may be a lower priority for women, particularly in socio-cultural and normative gendered contexts where pressures and expectations to bear children are high [27]. Where women have limited autonomy over reproductive decision-making, the delays involved in testing and treating pre-existing conditions or infections before pregnancy attempts occur may create difficulties for women. Counselling which directly acknowledges and addresses these delays provides opportunities to encourage pre-pregnancy planning and limit disengagement from care. For some women, pregnancy is not a goal. Ensuring their autonomy to decide against pregnancy is also a reproductive right and, therefore, sexual and reproductive health programmes must also support HIV-affected women and couples to make this choice with provision of, or linkage to, comprehensive contraceptive services [1].

### Creating enabling environments that reduce stigma associated with HIV and infertility

HIV-related stigma emerges as a major challenge to HIV-affected women and couples seeking services to minimize HIV risk during pregnancy attempts [28, 29]. To combat social stigma, programmes need to normalize pregnancy and childbearing desires and the use of HIV risk reduction strategies during pregnancy attempts [26]. In addition to HIV-related stigma, HIV-affected women and couples with suspected or diagnosed infertility may suffer psychosocial distress related to childbearing expectations, especially in societies where childbearing remains a strong expectation. This distress may be magnified in a setting where fertility is not routinely assessed and fertility care services that could resolve an infertility problem, and could reduce the stigma, may not be well established or supported [30].

Long-standing community impressions that HIV is associated with shorter life expectancy and potentially discounts a parent from long-term child-rearing may contribute to stigma associated with pregnancy and childbearing among HIV-affected women and couples. For HIV-infected women attempting pregnancy, antiretroviral therapy is a powerful option to reduce HIV transmission risk, with tremendous clinical benefits for the woman and prevention benefits for partners and the future child [13, 31]. However, HIV-infected individuals have long reported barriers to the initiation of antiretrovirals, citing personal and community perceptions that antiretroviral therapy use signals sickness and imminent death [32]. For HIV-affected women and couples, early provider-initiated discussions of fertility goals offer a prime opportunity to normalize antiretroviral initiation within the context of optimizing health prior to pregnancy and maximizing the likelihood of pregnancy [33].

Despite well-established directives on the rights of HIV-affected women and couples to attempt pregnancy, providers have voiced concerns about managing clinical and relationship complexities presented by HIV-serodiscordant couples wishing to become pregnant [26]. Thus, providers would value opportunities to develop skills to counsel non-disclosed HIV-affected women and couples without inadvertently disclosing HIV or subfertility status and triggering a cascade of poor outcomes, including patient/client distrust, couple discord and intimate partner violence [26]. Of utmost importance is that providers are able to counsel about fertility and HIV, with high sensitivity to social and cultural ideas about pregnancy among HIV-affected women and couples and perceptions of infertility.

### Creating enabling environments that encourage disclosure of HIV and fertility status to partners

For HIV-affected women and couples, disclosing HIV status can be one of the strongest links to health optimization, yet the act of disclosure is often met with trepidation and delays are common due to threats of violence, relationship dissolution and rejection [34]. Similarly, disclosure of infertility by HIV-affected women and couples can reduce HIV exposure during pregnancy attempts that are unlikely to succeed, but infertility disclosure may also result in these types of consequences. Environments supportive of reproductive decision-making must aim to foster the attainment of reproductive autonomy and support individual decision-making regarding the disclosure of HIV and/or infertility status to partners and providers. When disclosure is withheld, however, an environment with supportive providers continues working to maximize pre-pregnancy health and address HIV risk reduction.

For HIV-infected women with HIV-uninfected male partners, HIV status disclosure to a male partner potentially increases his personal HIV risk perception, his motivation to employ risk reduction methods and his engagement in reproductive decision-making [35]. When a heterosexual couple is mutually disclosed, male partners can take a more active role supporting reproductive decision-making including planning to delay pregnancy or seek fertility care to attempt pregnancy [26]. Models to increase male engagement and emphasize comfortable spaces for men, as well as women, have been successfully demonstrated in resource-constrained settings and can be adapted for programmes tailored to HIV risk reduction when pregnancy is attempted [36].

## Conclusions

We have identified and described four key values: understanding fertility care and an ability to identify potential fertility problems, providing equity of access to resources enabling informed decision-making about reproductive choices, creating enabling environments that reduce stigma associated with HIV and infertility and creating enabling environments that encourage disclosure of HIV status and fertility status to partners. These key values should remain central to guidelines, policies and programmes being developed to optimize pregnancy outcomes and reduce HIV risk among HIV-affected women and couples during pregnancy attempts. At this unique time in HIV prevention, when the power of antiretrovirals, including PrEP, and fertility interventions to eliminate HIV risk has been well established, HIV-affected women and couples no longer need to accept elevated HIV risk during pregnancy attempts nor forgo their desires for pregnancy. The imminent task for low-resource, high HIV-burdened settings is to develop acceptable and feasible services that are affordable and cost-effective to meet these needs.

Initially in the HIV epidemic, HIV-affected women and couples were discouraged from attempting pregnancy [37, 38]. The field has certainly progressed, as HIV is now a chronic condition in most societies, and there are great opportunities to help HIV-affected women and couples satisfy their fertility goals with minimal HIV risk while strengthening integrated health systems and fostering collaborations between fertility and HIV care providers. HIV-affected women and couples seeking services must encounter confident, informed providers who are equipped to initiate and engage in discussion about fertility desires, fertility care and HIV risk. Service integration provides opportunities for providers from the two disciplines to share experiences and best practices. HIV care providers, for example, can incorporate fertility screening into HIV care, recognize the importance of delaying pregnancy attempts when fertility may be compromised, provide access to fertility care options and provide counsel that recognizes socio-cultural childbearing expectations. In parallel, fertility care providers can adapt their practice to the context of HIV infection and recognize opportunities to reduce HIV risk, counsel about HIV prevention strategies and promote HIV testing and access to care.

Efforts to integrate services to improve outcomes among HIV-affected women and couples attempting pregnancy need to utilize evidence-based tools and approaches consistent with a sexual and reproductive health rights–based context [1]. This includes tools to expand provider knowledge and confidence when providing HIV prevention and fertility services for HIV-affected women and couples, campaigns to increase demand among HIV-affected women and couples for these services and appropriate systems to capture uptake and access to services. Research utilizing implementation science methods to capture usability and feasibility data is needed to evaluate various delivery models and determine the most cost-effective approaches. In addition, international clinical guidelines based on consensus recommendations from HIV prevention and fertility care researchers, providers, advocates and HIV-affected women and couples are urgently needed to spur the development of scalable programmes to reduce HIV risk during attempts to become pregnant.

In this commentary, we have described four key values to prioritize when integrating pre-pregnancy care and counselling services into HIV prevention and reproductive health services for HIV-affected women and couples. We identified many additional values including empowerment, religious beliefs and mental health services, but limited our descriptions here to the four that have dominated our discussions. Importantly, each HIV-affected individual seeking services presents a different HIV risk profile – whether in a seroconcordant partnership with risk of perinatal transmission or superinfection, HIV-uninfected with a partner of unknown status and the risk of sexual and perinatal transmission, unknown subfertility that necessitates prolonged pregnancy attempts or assisted reproductive care, or myriad other scenarios. Individuals prioritize values differently; programmes must consider each of these scenarios to ensure that their services accommodate all.

Programmes responsive to client values and preferences are inherently better positioned to meet client needs, fostering greater demand and uptake. Opportunities to incorporate pregnancy planning and fertility management exist through ongoing efforts to integrate HIV prevention and sexual and reproductive health programmes and the resulting programmes should be poised to fully respond to sexual and reproductive health needs. Minimizing HIV risk during pregnancy attempts for HIV-affected women and couples is a public health imperative. Tools to accomplish safer conception are available – and new tools developed alongside global consensus guidelines will add to this compendium. The impetus now is to develop value-driven, inclusive, scalable programmes to deliver interventions to minimize HIV transmission risk during pregnancy attempts with maximal coverage and impact.

## References

[1] United Nations (2014). Report of the Secretary General.

[2] Ngure K, Baeten J, Mugo N, Curran K, Vusha S, Heffron R (2014). “My intention was a child but I was very afraid”: fertility intentions and HIV risk perception among HIV-serodiscordant couples experiencing pregnancy in Kenya. AIDS Care.

[3] Matthews LT, Moore L, Crankshaw TL, Milford C, Mosery FN, Greener R (2014). South Africans with recent pregnancy rarely know partner's HIV serostatus: implications for serodiscordant couples interventions. BMC Public Health.

[4] Mugo NR, Heffron R, Donnell D, Wald A, Were EO, Rees H (2011). Increased risk of HIV-1 transmission in pregnancy: a prospective study among African HIV-1-serodiscordant couples. AIDS.

[5] Cooper D, Harries J, Myer L, Orner P, Bracken H, Zweigenthal V (2007). “Life is still going on”: reproductive intentions among HIV-positive women and men in South Africa. Soc Sci Med.

[6] Cooper D, Moodley J, Zweigenthal V, Bekker LG, Shah I, Myer L (2009). Fertility intentions and reproductive health care needs of people living with HIV in Cape Town, South Africa: implications for integrating reproductive health and HIV care services. AIDS Behav.

[7] Kaida A, Matthews LT, Kanters S, Kabakyenga J, Muzoora C, Mocello AR (2013). Incidence and predictors of pregnancy among a cohort of HIV-positive women initiating antiretroviral therapy in Mbarara, Uganda. PLoS One.

[8] Finocchario-Kessler S, Bastos FI, Malta M, Anderson J, Goggin K, Sweat M (2012). Discussing childbearing with HIV-infected women of reproductive age in clinical care: a comparison of Brazil and the US. AIDS Behav.

[9] Loutfy MR, Hart TA, Mohammed SS, Su D, Ralph ED, Walmsley SL (2009). Fertility desires and intentions of HIV-positive women of reproductive age in Ontario, Canada: a cross-sectional study. PLoS One.

[10] Matthews LT, Mukherjee JS (2009). Strategies for harm reduction among HIV-affected couples who want to conceive. AIDS Behav.

[11] Baeten JM, Donnell D, Ndase P, Mugo NR, Campbell JD, Wangisi J (2012). Antiretroviral prophylaxis for HIV prevention in heterosexual men and women. N Engl J Med.

[12] Thigpen MC, Kebaabetswe PM, Paxton LA, Smith DK, Rose CE, Segolodi TM (2012). Antiretroviral preexposure prophylaxis for heterosexual HIV transmission in Botswana. N Engl J Med.

[13] Cohen MS, Chen YQ, McCauley M, Gamble T, Hosseinipour MC, Kumarasamy N (2011). Prevention of HIV-1 infection with early antiretroviral therapy. N Engl J Med.

[14] Mmeje O, Cohen CR, Cohan D (2012). Evaluating safer conception options for HIV-serodiscordant couples (HIV-infected female/HIV-uninfected male): a closer look at vaginal insemination. Infect Dis Obstet Gynecol.

[15] Bailey RC, Moses S, Parker CB, Agot K, Maclean I, Krieger JN (2007). Male circumcision for HIV prevention in young men in Kisumu, Kenya: a randomised controlled trial. Lancet.

[16] Grossman D, Onono M, Newmann SJ, Blat C, Bukusi EA, Shade SB (2013). Integration of family planning services into HIV care and treatment in Kenya: a cluster-randomized trial. AIDS.

[17] Shade SB, Kevany S, Onono M, Ochieng G, Steinfeld RL, Grossman D (2013). Cost, cost-efficiency and cost-effectiveness of integrated family planning and HIV services. AIDS.

[18] Joint United Nations Programme on HIV/AIDS (2013). Global report: UNAIDS report on the global AIDS epidemic 2013.

[19] Cooper D, Mantell JE, Moodley J, Mall S (2015). The HIV epidemic and sexual and reproductive health policy integration: views of South African policymakers. BMC Public Health.

[20] Mascarenhas MN, Flaxman SR, Boerma T, Vanderpoel S, Stevens GA (2012). National, regional, and global trends in infertility prevalence since 1990: a systematic analysis of 277 health surveys. PLoS Med.

[21] Bunting L, Boivin J (2010). Development and preliminary validation of the fertility status awareness tool: FertiSTAT. Hum Reprod.

[22] Mugo NR, Hong T, Celum C, Donnell D, Bukusi EA, John-Stewart G (2014). Pregnancy incidence and outcomes among women receiving preexposure prophylaxis for HIV prevention: a randomized clinical trial. JAMA.

[23] Moodley J, Cooper D, Mantell JE, Stern E (2014). Health care provider perspectives on pregnancy and parenting in HIV-positive individuals in South Africa. BMC Health Serv Res.

[24] Kawale P, Mindry D, Phoya A, Jansen P, Hoffman RM (2015). Provider attitudes about childbearing and knowledge of safer conception at two HIV clinics in Malawi. Reprod Health.

[25] Matthews LT, Milford C, Kaida A, Ehrlich MJ, Ng C, Greener R (2014). Lost opportunities to reduce periconception HIV transmission: safer conception counseling by South African providers addresses perinatal but not sexual HIV transmission. J Acquir Immune Defic Syndr.

[26] Crankshaw TL, Mindry D, Munthree C, Letsoalo T, Maharaj P (2014). Challenges with couples, serodiscordance and HIV disclosure: healthcare provider perspectives on delivering safer conception services for HIV-affected couples, South Africa. J Int AIDS Soc.

[27] Kastner J, Matthews LT, Flavia N, Bajunirwe F, Erikson S, Berry NS (2014). Antiretroviral therapy helps HIV-positive women navigate social expectations for and clinical recommendations against childbearing in Uganda. AIDS Res Treat.

[28] Goggin K, Mindry D, Beyeza-Kashesya J, Finocchario-Kessler S, Wanyenze R, Nabiryo C (2014). Our hands are tied up”: current state of safer conception services suggests the need for an integrated care model. Health Care Women Int.

[29] Gogna ML, Pecheny MM, Ibarlucia I, Manzelli H, Lopez SB (2009). The reproductive needs and rights of people living with HIV in Argentina: health service users’ and providers’ perspectives. Soc Sci Med.

[30] Dyer SJ, Abrahams N, Mokoena NE, Lombard CJ, van der Spuy ZM (2005). Psychological distress among women suffering from couple infertility in South Africa: a quantitative assessment. Hum Reprod.

[31] Kilewo C, Karlsson K, Ngarina M, Massawe A, Lyamuya E, Swai A (2009). Prevention of mother-to-child transmission of HIV-1 through breastfeeding by treating mothers with triple antiretroviral therapy in Dar es Salaam, Tanzania: the Mitra Plus study. J Acquir Immune Defic Syndr.

[32] Curran K, Ngure K, Shell-Duncan B, Vusha S, Mugo NR, Heffron R (2014). ‘If I am given antiretrovirals I will think I am nearing the grave’: Kenyan HIV serodiscordant couples’ attitudes regarding early initiation of antiretroviral therapy. AIDS.

[33] Crankshaw TL, Matthews LT, Giddy J, Kaida A, Ware NC, Smit JA (2012). A conceptual framework for understanding HIV risk behavior in the context of supporting fertility goals among HIV-serodiscordant couples. Reprod Health Matters.

[34] Tam M, Amzel A, Phelps BR (2015). Disclosure of HIV serostatus among pregnant and postpartum women in sub-Saharan Africa: a systematic review. AIDS Care.

[35] Mindry D, Maman S, Chirowodza A, Muravha T, van Rooyen H, Coates T (2011). Looking to the future: South African men and women negotiating HIV risk and relationship intimacy. Cult Health Sex.

[36] Onono M, Guze MA, Grossman D, Steinfeld R, Bukusi EA, Shade S (2015). Integrating family planning and HIV services in western Kenya: the impact on HIV-infected patients’ knowledge of family planning and male attitudes toward family planning. AIDS Care.

[37] Kass NE (1994). Policy, ethics, and reproductive choice: pregnancy and childbearing among HIV-infected women. Acta Paediatr.

[38] Centers for Disease Control (1985). Recommendations for assisting in the prevention of perinatal transmission of human T-lymphotropic virus type III/lymphadenopathy-associated virus and acquired immunodeficiency syndrome. MMWR Morb Mortal Wkly Rep.

# Sub article: Fundamental concerns of women living with HIV around the implementation of Option B+

**DOI:** 10.7448/IAS.18.6.20286

**Published:** 2015-12-01

**Authors:** Rebecca Matheson, Suzette Moses-Burton, Amy C Hsieh, Sophie Dilmitis, Margaret Happy, Eunice Sinyemu, Sophie O Brion, Aditi Sharma

**Affiliations:** 1 International Community of Women Living with HIV (ICW), Nairobi, Kenya; 2 Global Network of People Living with HIV (GNP+), Amsterdam, The Netherlands; 3 Global Network of People Living with HIV (GNP+), Brooklyn, NY, USA; 4 International Community of Women Living with HIV (ICW), Harare, Zimbabwe; 5 International Community of Women Living with HIV (ICW), Kampala, Uganda; 6 International Community of Women Living with HIV (ICW), Lusaka, Zambia; 7 Network of Zambian People Living with HIV (NZP+), Lusaka, Zambia; 8 International Community of Women Living with HIV (ICW), Washington, DC, USA; 9 Global Network of People Living with HIV (GNP+), Brighton, UK

**Keywords:** adherence, eMTCT, implementation science, PMTCT, qualitative research, retention, vertical transmission

## Abstract

**Introduction:**

In 2011, the Global Plan towards the Elimination of New HIV Infections among Children by 2015 and Keeping Their Mothers Alive was launched to scale up efforts to comprehensively end vertical HIV transmission and support mothers living with HIV in remaining healthy. Amidst excitement around using treatment as prevention, Malawi's Ministry of Health conceived Option B+, a strategy used to prevent vertical transmission by initiating all pregnant and breastfeeding women living with HIV on lifelong antiretroviral therapy, irrespective of CD4 count. In 2013, for programmatic and operational reasons, the WHO officially recommended Option B+ to countries with generalized epidemics, limited access to CD4 testing, limited partner testing, long breastfeeding duration or high fertility rates.

**Discussion:**

While acknowledging the opportunity to increase treatment access globally and its potential, this commentary reviews the concerns of women living with HIV about human rights, community-based support and other barriers to service uptake and retention in the Option B+ context. Option B+ intensifies many of the pre-existing challenges of HIV prevention and treatment programmes. As women seek comprehensive services to prevent vertical transmission, they can experience various human rights violations, including lack of informed consent, involuntary or coercive HIV testing, limited treatment options, termination of pregnancy or coerced sterilization and pressure to start treatment. Yet, peer and community support strategies can promote treatment readiness, uptake, adherence and lifelong retention in care; reduce stigma and discrimination; and mitigate potential violence stemming from HIV disclosure. Ensuring available and accessible quality care, offering food support and improving linkages to care could increase service uptake and retention. With the heightened focus on interventions to reach pregnant and breastfeeding women living with HIV, a parallel increase in vigilance to secure their health and rights is critical.

**Conclusion:**

The authors conclude that real progress towards reducing vertical transmission and achieving viral load suppression can only be made by upholding the human rights of women living with HIV, investing in community-based responses, and ensuring universal access to quality healthcare. Only then will the opportunity of accessing lifelong treatment result in improving the health, dignity and lives of women living with HIV, their children and families.

## Introduction

Option B+ could be good, but we still lack information. – Woman living with HIV, Uganda [1]

In 2011, the Global Plan towards the Elimination of New HIV Infections among Children by 2015 and Keeping Their Mothers Alive (Global Plan) was launched to scale up efforts to comprehensively end vertical HIV transmission and support mothers living with HIV in remaining healthy [2]. This undertaking re-emphasized the necessity of the four-pillared approach, also known as *prevention-of-mother-to-child-transmission* [3, 4], (The term *comprehensive prevention of vertical transmission* is used where possible in this commentary in line with developing usage among the community of people living with HIV. For a fuller discussion on terminology, see Refs. [3, 4]), which aims to 1) prevent HIV acquisition among women of reproductive age; 2) provide appropriate care to meet the family planning needs of women living with HIV; 3) support pregnant women living with HIV to prevent vertical transmission; and 4) provide care, treatment and support to women living with HIV, their children and families [5].

Concurrently, excitement around treatment as prevention was mounting [6–8]. Against this backdrop in 2011, Malawi's Ministry of Health conceived and began implementing a new strategy to prevent vertical transmission that provides lifelong antiretroviral therapy (ART) to all pregnant and breastfeeding women living with HIV, irrespective of their CD4 count. Expanding on an existing WHO recommendation to HIV programme managers called *Option B*, which offers triple combination antiretrovirals (ARVs) as prophylaxis only during pregnancy and breastfeeding, Malawi's approach was dubbed *Option B+*. Malawi's primary rationale was the lack of access to CD4 cell count equipment and systems for successful referral to ART services for women who need treatment for their own health, the risk of viral rebound after ARV cessation and inconsistent breastfeeding cessation patterns, together causing increased HIV acquisition risk to a sexual partner or child [9, 10].

In 2013, the WHO officially recommended Option B+, for programmatic and operational reasons, particularly in countries with generalized epidemics, high fertility rates or long breastfeeding duration [11]. These were “conditional recommendations” based on “low-quality evidence” [11]. WHO additionally reasoned that Option B+ offered simplified implementation with a standardized drug regimen (the same as for non-pregnant individuals) and was cost-effective [11].

In November 2014, world leaders launched the Fast-Track Strategy to End the AIDS Epidemic by 2030, in which Option B+ plays a leading role [12]. To date, 19 of 22 Global Plan countries have committed to Option B+, though most are in the early or scale-up phases of implementation [13].

Communities of people living with HIV [1, 14–16] and clinicians [17–19] have held mixed and evolving perceptions of Option B+ since its inception. Women living with HIV understand the opportunity Option B+ presents to increase treatment access globally and its potential (e.g. improved mothers’ and babies’ health, reduced stigma, opportunities to breastfeed). However, few articles discuss the need to uphold human rights, invest in community-based support and address barriers to service uptake and retention as being essential in order to comprehensively end vertical transmission. This commentary reviews these concerns raised by women living with HIV regarding Option B+ implementation and calls for funding to support its examination through women's lived experiences.

## Discussion

As global momentum and pressure on countries to meet Global Plan targets have increased, so has the pressure on pregnant and breastfeeding women to be tested for HIV and initiate early and lifelong treatment. Option B+ intensifies many of the pre-existing challenges of HIV prevention and treatment programmes. With the heightened focus on interventions to reach pregnant and breastfeeding women, a parallel increase in vigilance to secure their health and rights is critical.

### Upholding human rights

Before I start on B+, I should be informed of all options and all of the advantages … so that I make informed decisions based on the benefits that are there. – Woman living with HIV, Uganda [1]

A primary concern regarding Option B+ implementation is whether women's human rights are upheld and what the consequences are for rights violations. A rights-based approach to end vertical transmission requires, at a minimum, that services be consistent with international human rights obligations [20–27]. Accordingly, women living with HIV have an autonomous right to make fully informed and voluntary decisions about whether to have an HIV test, learn the result, start or opt out of treatment, receive support for whichever decision they make and determine their sexual and reproductive lives free from stigma and discrimination [28–32]. During a 2012 consultation held in Malawi and Uganda, women living with HIV expressed concern about whether women are offered sufficient information about why and how to start lifelong treatment, time to reflect and the opportunity to choose [1].

A South Africa programme is pilot testing a “rapid ART initiation approach” as part of Option B+ [33]. In this method, women are enrolled in ART within the same week of their first antenatal care visit [33, 34]. The programme boasts a 97.0% ART initiation rate, with 90.8% initiating within the same day that treatment eligibility is determined [33].

However, given insufficient time to process the news, along with the information asymmetry and power disparity between client and provider [35], women in Malawi have reported feeling pressured to accept lifelong treatment without being fully informed of possible side effects, understanding the commitment and having linkage to care and support for adherence [1]. Such rapid initiation of lifelong treatment requires analysis as to whether the process meets international human rights standards for informed consent.

Across the four pillars, women can experience a spectrum of coercive practices, including lack of informed consent, involuntary or coercive HIV testing [36, 37], limited contraception [38] or treatment options, termination of pregnancy or coerced sterilization [39–44] and pressure to accept particular contraceptives or start treatment [37, 45]. These practices lead to disempowerment around testing and treatment choices, which can discourage women from seeking care and are counterproductive to meeting public health goals [30].

Indeed, early loss to follow-up has been a challenge for many countries implementing Option B+. For instance, in Malawi, compared to individuals who started ART for their own health, women who started ART while pregnant were five times less likely to return to the clinics after the initial visit (when they initiated ART) [46]. On average, 17 and 22% of all pregnant women starting ART under Option B+ dropped out of care in the first six months and year of therapy, respectively [46]. Women who started ART while breastfeeding were twice as likely to miss their first follow-up visit [46].

Additionally, non-discriminatory care, free from HIV-related stigma, is also vital to comprehensively prevent vertical transmission. Unlike previous prophylactic protocols, women must feel comfortable and supported to begin treatment “earlier” and remain in lifelong care. Yet, women reported receiving discriminatory care, where service providers do not treat them with dignity (e.g. yelling derogatory statements) and violate other human rights (e.g. disclosing serostatus, failing to provide correct or full information) [47]. Such discriminatory care provided by clinic staff to clients during labour and delivery and when they collected ARVs preceded the rollout of Option B+ in Malawi and remains a primary concern for women living with HIV [48].

Additionally, women are concerned that Option B+ may exacerbate inequities around treatment access by men and non-pregnant women [1, 49]. Some women in Malawi and Uganda feared the potential increase in domestic violence if one partner discovers her serostatus and/or starts treatment before the other and is accused of “bringing the virus home” [1]. Yet, policies that have attempted to engage partners (e.g. Uganda's requirement for pregnant women to bring their partners to antenatal visits) have also produced unintended consequences. As one Ugandan woman shared, “Women have chose[n] to hire *boda boda* (motorcycle taxi drivers) to go with them to access the services” [1]. Consequently, countries implementing Option B+ must consider how to prevent negative consequences of ART prioritization and design partner engagement policies that do not put women at greater risk.

Improving the circumstances around offering lifelong ART, by focusing on quality rather than quantity, is needed to support women to make informed decisions regarding timely treatment initiation and to remain in lifelong care [50]. In particular, programmes seeking to end vertical transmission should 1) involve women living with HIV in programme design and implementation; 2) train healthcare workers to provide non-discriminatory care, provide sufficient information and obtain informed consent without coercion; and 3) provide mechanisms for women to raise and address concerns about human rights violations (e.g. patient representatives, healthcare facility ombudsman or complaints mechanism). Finally, funding networks of women living with HIV, to provide needed information, rights education and other support, provides an additional opportunity to ensure that human rights are respected, protected and fulfilled.

### Investing in community-based support

There should be education for everybody that states clearly when people should start treatment so people are prepared. The doctors and those of us in support groups know a little but we need to disseminate the information. – Woman living with HIV, Malawi [1]

Community-based support is critical to Option B+ implementation. Communities play an important role in facilitating treatment readiness, support and retention in care. After years of being told to wait before starting ART, women need education and awareness of the benefits of earlier treatment initiation for their own health and how to manage lifelong treatment and side effects.

Personal readiness to start treatment is complex and motivated by many personal and social factors [51, 52]. Women living with HIV from Malawi and Uganda have warned that starting patients on treatment before they feel ready would not be conducive to adherence, retention or good health [1]. In Tanzania, women acknowledged the eventual need for treatment for their own health, but shared that they may lose motivation to remain in treatment after the risk of transmission to the child has passed, due to fear of the medications’ side effects and not feeling ready to remain on lifelong treatment [53]. Additionally, studies have suggested that more women who start ART for their own health remain in care than those who start for other reasons [54, 55]. Yet, as with treatment programmes that existed before Option B+, stigma and discrimination at home and in the community, divorce and physical violence have caused many women to decline treatment or hide their medications to prevent unintended disclosure [56, 57].

Peer and community support strategies can promote treatment uptake, adherence and lifelong retention in care [1, 46, 58–64]. Peer-to-peer strategies can further help to reduce stigma and discrimination and mitigate potential violence stemming from disclosure of HIV status [1]. High quality education and support groups meeting at the hospital or in the villages have been found to facilitate access and retention in care [48]. Community-based follow-up, such as home visits with women–infant pairs as in Zambia [65], has also improved antenatal care attendance. Women from Malawi and Uganda highlighted the need for clear information and education in communities, peer-to-peer counselling and community-led retention and adherence models to improve literacy, preparedness [51] and agency in order to enable women to assert their rights [1].

### Overcoming barriers to service uptake and retention

If I am coming from some place and you are referring me 40 kilometers, you find that the mother does not have that transport and this will hurt adherence. – Woman living with HIV, Uganda [1]

Women who commence lifelong ART, especially those with young children, shoulder new burdens arising from long-term routine clinic appointments. With Option B+, women experience temporal, financial (i.e. transportation costs, missing work), relational (e.g. permission from partner), emotional and physical (e.g. side effects) costs for the remainder of their lives. These costs affect women's ability to seek HIV care and adhere to treatment regimens [1] and have caused some women in Malawi to stop ART [66].

When women have been able to attend appointments, dysfunctional clinic and health systems – including healthcare worker shortages, long wait times and distances to clinics – disincentivize them from seeking care [67]. Studies in Tanzania and Malawi highlighted that maintenance of regular supplies of HIV-related test kits and medications are important in ensuring that all women in need of vertical transmission prevention services are reached [35, 67, 68].

Women living with HIV affirmed that ensuring available and easily accessible quality care, particularly by having follow-up and ARV distribution points closer to communities, could increase service uptake [1]. Access to food and food support (e.g. supplements for their infants, money from support groups) are also important facilitators to care [48]. A study of 141 health facilities in Malawi found that the number of women per HIV testing and counseling (HTC) counsellor, HIV-related test kit availability and the “model of care” affected treatment uptake; district location, patient volume and the model of care affected retention in care [69]. Improving linkages between antenatal and ARV services is important [70–73], but further research with women living with HIV is crucial to determining which models of care will most successfully support treatment initiation and lifelong ART [69].

## Conclusions

Option B+ has expanded treatment access for many pregnant and breastfeeding women living with HIV. However, real progress towards reducing vertical transmission and achieving viral load suppression can only be made by upholding the human rights of women living with HIV, investing in community-based responses and ensuring universal access to quality healthcare. Only then will the opportunity of accessing lifelong treatment result in improving the health, dignity and lives of women living with HIV, their children and families.

To meet Global Plan targets, governments and programmes seeking to end vertical transmission must do the following:

*Uphold human rights* by ensuring that service providers 1) always provide women sufficient information and time to make informed decisions regarding treatment for their health and the health of the child, 2) do not coerce women into accepting lifelong treatment should another option be desired, 3) respect confidentiality and 4) provide non-discriminatory care.

*Invest in community-based responses* to improve linkages to services, treatment literacy, preparedness and agency to enable women to receive quality services and adhere to treatment. This investment includes financial support to deliver community-based services, which are the backbone of healthcare systems, support groups, peer supporters and mentor mothers living with HIV, and linkages to networks and organizations of women living with HIV.

*Overcome barriers to service uptake and retention* by ensuring access to quality healthcare and providing decentralized services to address common challenges (e.g. distance to clinics, transport costs and long waiting times). This includes ensuring adequate supplies of ARVs, other medications and diagnostic tools as well as integration with other programmes (e.g. maternal and child health, nutrition, mental health) in ways that deliver the best care for women living with HIV and their families.

The experience and meaningful involvement of women living with HIV regarding design, implementation, monitoring and evaluation of Option B+ are crucial to comprehensively prevent vertical transmission. Teresia Njoki Otieno, speaking as ICW Global Chair at the launch of the Fast-Track strategy, reaffirmed, “We should end this epidemic, but we can only do this if we put women living with HIV at the centre”.

## References

[1] Rebekah Webb Consulting, International Community of Women Living with HIV (ICW), Global Network of People Living with HIV (GNP+) Understanding the perspectives and/or experiences of women living with HIV regarding Option B+ in Uganda and Malawi. Global Network of People Living with HIV [Internet].

[2] Joint United Nations Programme on HIV/AIDS (UNAIDS) (2011). Global plan towards the elimination of new infections in children by 2015 and keeping mothers alive: 2011–2015. New York [Internet].

[3] Dilmitis S, Edwards O, Hull B, Margolese S, Mason N, Namiba A (2012). Language, identity and HIV: why do we keep talking about the responsible and responsive use of language? Language matters. J Int AIDS Soc.

[4] United Nations Population Fund (2012). Preventing HIV and unintended pregnancies: strategic framework 2011–2015 [Internet]. http://www.unfpa.org/sites/default/files/pub-pdf/V2_web_P1P2_framework%2022.8.12.pdf.

[5] International Treatment Preparedness Coalition (2011). Missing the Target 9: the long walk: ensuring comprehensive care for women and families to end vertical HIV transmission: community experiences of efforts to prevent vertical transmission in ten countries [Internet].

[6] Cohen MS, Chen YQ, McCauley M, Gamble T, Hosseinipour MC, Kumarasamy N (2011). Prevention of HIV-1 infection with early antiretroviral therapy. N Engl J Med.

[7] Cohen J (2011). Breakthrough of the year: HIV treatment as prevention. Science.

[8] UNAIDS (2011). Groundbreaking trial results confirm HIV treatment prevents transmission of HIV [Press Release] [Internet].

[9] Center for Disease Control and Prevention (CDC) (2013). Impact of an innovative approach to prevent mother-to-child transmission of HIV – Malawi, July 2011–September 2012. Morb Mortal Wkly Rep.

[10] WHO Regional Office for Africa. Implementation of Option B+ for prevention of mother-to-child transmission of HIV: the Malawi experience (2014). Republic of Congo. pp. xi, 2–3 [Internet]..

[11] WHO 7.1.2 When to start ART in pregnant and breastfeeding women. In: World Health Organization. Consolidated guidelines on the use of antiretroviral drugs for treating and preventing HIV infection: recommendations for a public health approach.

[12] UNAIDS Fast Track: ending the AIDS epidemic by 2030.

[13] IATT (2015). Option B+ countries and PMTCT regimen. New York [Internet].

[14] WHO (2012). Discussion forum on option B+ with civil society leaders: report of a conference call [Internet].

[15] Welbourn A, Binder L (2013). Compulsion versus compassion: HIV treatment for women and children. 50.50 inclusive democracy [Internet].

[16] Ngarina M, Tarimo EA, Naburi H, Kilewo C, Mwanyika-Sando M, Chalamilla G (2014). Women's preferences regarding infant or maternal antiretroviral prophylaxis for prevention of mother-to-child transmission of HIV during breastfeeding and their views on Option B+ in Dar es Salaam, Tanzania. PLoS One.

[17] Coutsoudis A, Goga A, Desmond C, Barron P, Black V, Coovadia H (2013). Is option B+ the best choice?. Lancet.

[18] De Cock KM, el-Sadr WM (2013). When to start ART in Africa – an urgent research priority. N Engl J Med.

[19] Van de Perre P, Tylleskär T, Delfraissy JF, Nagot N (2013). How evidence based are public health policies for prevention of mother to child transmission of HIV?. BMJ.

[20] United Nations General Assembly (UNGA) (1948). Universal declaration of human rights. 217 A (III). Article 25 [Internet].

[21] UNGA (1966). International covenant on economic, social, and cultural rights. United Nations Treaty Series. 993:3, Article 12 [Internet].

[22] UNGA (1965). International convention on the elimination of all forms of racial discrimination. United Nations Treaty Series. 660:195, Article 11 [Internet].

[23] UNGA (1979). Convention on the elimination of all forms of discrimination against women. United Nations Treaty Series. 1249:13, Article 12 [Internet].

[24] UNGA (1989). Convention on the rights of the child. United Nations Treaty Series. 1577:3, Article 24 [Internet].

[25] UNGA (2007). Convention on the rights of persons with disabilities [resolution]. A/RES/61/106, Article 25 [Internet].

[26] Organization of African Unity (OAU) (1981). African Charter on human and peoples’ rights (“Banjul Charter”). CAB/LEG/67/3 rev. 5, 21 I.L.M. 58, Article 16 [Internet].

[27] United Nations Committee on Economic, Social and Cultural Rights (2000). General Comment No. 14: the right to the highest attainable standard of health (Art. 12 of the Covenant). E/C.12/2000/4 [Internet].

[28] Gruskin G, Bogecho D, Ferguson L (2010). ‘Rights-based approaches’ to health policies and programs: articulations, ambiguities and assessment. J Public Health Policy.

[29] UNAIDS (2006). International guidelines on HIV/AIDS and human rights 2006 consolidated version. Geneva [Internet].

[30] CDC, WHO (2010). PMTCT – generic training package participant manual. In: Prevention of mother-to-child transmission of HIV (PMTCT) generic training package [Internet].

[31] King EJ, Maman S, Wyckoff SC, Pierce MW, Groves AK (2013). HIV testing for pregnant women: a rights-based analysis of national policies. Global Public Health.

[32] WHO Box 10.4 Key implementation considerations: moving to lifelong ART for all pregnant and breastfeeding women (option B+). World Health Organization. Consolidated guidelines on the use of antiretroviral drugs for treating and preventing HIV infection: recommendations for a public health approach.

[33] Black S, Zulliger R, Myer L, Marcus R, Jeneker S, Taliep R (2013). Safety, feasibility and efficacy of a rapid ART initiation in pregnancy pilot programme in Cape Town, South Africa. S Afr Med J.

[34] Black S, Zulliger R, Marcus R, Mark D, Myer L, Bekker LG (2014). Acceptability and challenges of rapid ART initiation among pregnant women in a pilot programme, Cape Town, South Africa. AIDS Care.

[35] Gourlay A, Wringe A, Birdthistle I, Mshana G, Michael D, Urassa M (2014). “It is like that, we didn't understand each other”: exploring the influence of patient-provider interactions on prevention of mother to child transmission of HIV service use in rural Tanzania. PLoS One.

[36] Mandhivanan P, Krupp K, Kulkami V, Vaidya N, Shaheen R, Philpott S (2014). HIV testing among pregnant women living with HIV in India: are private healthcare providers routinely violating women's human rights?. BMC Int Health Hum Rights.

[37] Hardon A, Vernooij E, Bongololo-Mbera G, Cherutich P, Desclaux A, Kyaddondo D (2012). Women's views on consent, counseling and confidentiality in PMTCT: a mixed-methods study in four African countries. BMC Public Health.

[38] Hillard S, Gutin SA, Rose CD (2014). Messages on pregnancy and family planning that providers give women living with HIV in the context of a positive health, dignity, and prevention intervention in Mozambique. Int J Women's Health.

[39] ICW (2009). The forced and coerced sterilization of HIV positive women in Namibia [Internet].

[40] Women of the Asia Pacific Network of People Living with HIV (2010). Positive and pregnant: how dare you. A study on access to reproductive and maternal health care for women living with HIV in Asia.

[41] Kendal T Reproductive rights violations experienced by women with HIV in Mesoamerica.

[42] Mthembu P, Essack Z, Strode A (2012). “I feel like half a woman all the time”: a qualitative report of HIV positive women's experiences of coerced and forced sterilizations [Internet].

[43] Center for Reproductive Rights (2008). At risk rights violations of HIV-positive women in Kenyan health facilities.

[44] Kasiva F, Kiio G (2012). Robbed of choice: forced and coerced sterilization experiences of women living with HIV in Kenya.

[45] Vernooij E, Ardon A (2013). “What mother wouldn't want to save her baby?” HIV testing and counseling practices in a rural Ugandan antenatal clinic. Cult Health Sex.

[46] Tenthani L, Haas AD, Tweya H, Jahn A, van Oosterhout JJ, Chimbwandira F (2014). Retention in care under universal antiretroviral therapy for HIV infected pregnant and breastfeeding women (“Option B+”) in Malawi. AIDS.

[47] ICW, GNP+ (2014). Quality of family planning services and integration in the prevention of vertical transmission context: perspectives and experiences of women living with HIV and service providers in Cameroon, Nigeria, and Zambia. Global Network of People Living with HIV [Internet].

[48] Iroezi ND, Mindry D, Kawale P, Chikowi G, Jansen PA, Hoffman RM (2013). A qualitative analysis of the barriers and facilitators to receiving care in a prevention of mother-to-child program in Nkhoma, Malawi. Afr J Reprod Health.

[49] Dovel K, Yeatman S, Watkins S, Poulin M (2015). Men's heightened risk of AIDS-related death: the legacy of gendered HIV testing and treatment strategies. AIDS.

[50] Mnyani CN, Marinda E, Struthers H, Gulley M, Machepa R, McIntyre J (2014). Timing of antenatal care and ART initiation in HIV-infected pregnant women before and after introduction of NIMART. S Afr J HIV Med.

[51] Gebrekristos HT, Mlisana KP, Karim Q (2005). Patients’ readiness to start highly active antiretroviral treatment for HIV. BMJ.

[52] Orrell C (2005). Antiretroviral adherence in a resource-poor setting. Curr HIV/AIDS Rep.

[53] Ngarina M, Tarimo EA, Naburi H, Kilewo C, Mwanyika-Sando M, Chalamilla G (2014). Women's preferences regarding infant or maternal antiretroviral prophylaxis for prevention of mother-to-child transmission of HIV during breastfeeding and their views on Option B+ in Dar es Salaam, Tanzania. PLoS One.

[54] Nachega JB, Uthman OA, Anderson J, Peltzer K, Wampold S, Cotton MF (2012). Adherence to antiretroviral therapy during and after pregnancy in low, middle and high income countries: a systematic review and meta-analysis. AIDS.

[55] Giordano TP, Hartman C, Gifford AL, Backus LI, Morgan RO (2009). Predictors of retention in HIV care among a national cohort of US veterans. HIV Clin Trials.

[56] Medley A, Garcia-Moreno C, McGill S, Maman S (2004). Rates, barriers and outcomes of HIV serostatus disclosure among women in developing countries: implications for prevention of mother-to-child transmission programs. Bull World Health Organ.

[57] Anglewicz P, Chintsanya J (2011). Disclosure of HIV status between spouses in rural Malawi. AIDS Care.

[58] Rosenberg NE, van Lettow M, Tweya H, Kapito-Tembo A, Bourdon CM, Cataldo F (2014). Improving PMTCT uptake and retention services through novel approaches in peer-based family-supported care in the clinic and community: a 3-arm cluster randomized trial (PURE Malawi). J Acquir Immune Defic Syndr.

[59] Kim MH, Ahmed S, Buck WC, Preidis GA, Hosseinipour MC, Bhalakia A (2012). The Tingathe programme: a pilot intervention using community health workers to create a continuum of care in the prevention of mother to child transmission of HIV (PMTCT) cascade of services in Malawi. J Int AIDS Soc.

[60] Rosenberg NE, Pettifor A, Kamanga G, Bonongwe N, Mapanje C, Hoffman I (2013). Social networks of STI patients have higher STI and HIV prevalence than social networks of community controls. Sex Transm Infect.

[61] Decroo T, Van Damme W, Kegels G, Remartinez D, Rasschaert F (2012). Are expert patients an untapped resource for ART provision in sub-Saharan Africa?. AIDS Res Treat.

[62] Decroo T, Telfer B, Biot M, Maïkéré J, Dezembro S, Cumba LI (2011). Distribution of antiretroviral treatment through self-forming groups of patients in Tete Province, Mozambique. J Acquir Immune Defic Syndr.

[63] Teasdale C, Besser M (2008). Enhancing PMTCT programs through psychosocial support and empowerment of women: the Mothers2Mothers model of care. S Afr J HIV Med.

[64] Bekker LG, Myer L, Orrell C, Lawn S, Wood R (2006). Rapid scale-up of a community based HIV treatment service: programme performance over 3 consecutive years in Guguletu, South Africa. S Afr Med J.

[65] Herlihy JM, Hamomba L, Bonawitz R, Goggin CE, Sambambi K, Mwale J (2015). Integration of PMTCT and antenatal services improves combination antiretroviral therapy cART uptake for HIV-positive pregnant women in Southern Zambia – a prototype for Option B+?. J Acquir Immune Defic Syndr.

[66] Tweya H, Gugsa S, Hosseinipour M, Speight C, Ng'ambi W, Bokosi M (2014). Understanding factors, outcomes and reasons for loss to follow-up among women in Option B+ PMTCT programme in Lilongwe, Malawi. Trop Med Int Health.

[67] Gourlay A, Mshana G, Wringe A Barriers to uptake of prevention of mother-to-child transmission of HIV services in rural Tanzania: a qualitative study.

[68] Kim MH, Ahmed S, Hosseinipour MC, Giordano TP, Chiao EY, Yu X (2015). Implementation and operational research: the impact of option B+ on the antenatal PMTCT cascade in Lilongwe, Malawi. J Acquir Immune Defic Syndr.

[69] van Lettow M, Bedell R, Mayuni I, Mateyu G, Landes M, Chan AK (2014). Towards elimination of mother-to-child transmission of HIV: performance of different models of care for initiating lifelong antiretroviral therapy for pregnant women in Malawi (Option B+). J Int AIDS Soc.

[70] Stinson K, Jennings K, Myer L (2013). Integration of antiretroviral therapy services into antenatal care increases treatment initiation during pregnancy: a cohort study. PLoS One.

[71] Mandala J, Torpey K, Kasonde P, Kabaso M, Dirks R, Suzuki C (2009). Prevention of mother-to-child transmission of HIV in Zambia: implementing efficacious ARV regimens in primary health centers. BMC Public Health.

[72] Hussain A, Moodley D, Naidoo S, Esterhuizen TM (2011). Pregnant women's access to PMTCT and ART services in South Africa and implications for universal antiretroviral treatment. PLoS One.

[73] Chinkonde JR, Sundby J, Martinson F (2009). The prevention of mother-to-child HIV transmission programme in Lilongwe, Malawi: why do so many women drop out. Reprod Health Matters.

# Sub article: How does living with HIV impact on women's mental health? Voices from a global survey

**DOI:** 10.7448/IAS.18.6.20289

**Published:** 2015-12-01

**Authors:** Luisa Orza, Susan Bewley, Carmen H Logie, Elizabeth Tyler Crone, Svetlana Moroz, Sophie Strachan, Marijo Vazquez, Alice Welbourn

**Affiliations:** 1 ATHENA Network, London, UK; 2 Salamander Trust, London, UK; 3 Women's Health Academic Centre, King's College London, London, UK; 4 Factor-Inwentash Faculty of Social Work, University of Toronto, Toronto, ON, Canada; 5 ATHENA Network, Seattle, WA, USA; 6 Eurasian Women's Network on AIDS, Kiev, Ukraine; 7 UNAIDS Dialogue Platform, London, UK

**Keywords:** HIV, women, human rights, gender-based violence, mental health, values and preferences, survey, intimate partner violence, evidence base

## Abstract

**Introduction:**

Women living with HIV experience a disproportionate burden of mental health issues. To date, global guidelines contain insufficient guidance on mental health support, particularly regarding perinatal care. The aim of this article is to describe the extent and impact of mental health issues as experienced by women living with HIV on their sexual and reproductive health and human rights (SRH&HR).

**Methods:**

A global, mixed-methods, user-led and designed survey on SRH&HR of women living with HIV was conducted using snowball sampling, containing an optional section exploring mental health issues. Statistical quantitative data analysis included descriptive statistics, correlation and multiple linear regression analysis for the mental health responses. Thematic analysis of open free-text responses was performed for qualitative data.

**Results:**

A total of 832 respondents from 94 countries participated in the online survey with 489 responses to the optional mental health section. Of the respondents, 82% reported depression symptoms and 78% rejection. One-fifth reported mental health issues before HIV diagnosis. Respondents reported experiencing a 3.5-fold higher number of mental health issues after diagnosis (8.71 vs 2.48, *t*[488]=23.00, *p*<0.001). Nearly half (*n*=224; 45.8%) had multiple socially disadvantaged identities (SDIs). The number of SDIs was positively correlated with experiencing mental health issues (*p*<0.05). Women described how mental health issues affected their ability to enjoy their right to sexual and reproductive health and to access services. These included depression, rejection and social exclusion, sleep problems, intersectional stigma, challenges with sexual and intimate relationships, substance use and sexual risk, reproductive health barriers and human rights (HR) violations. Respondents recommended that policymakers and clinicians provide psychological support and counselling, funding for peer support and interventions to challenge gender-based violence and to promote HR.

**Conclusions:**

Interventions addressing intersecting stigmas and any especial impacts of diagnosis during pregnancy are required to ensure women's SRH&HR. Global policy guidelines regarding women living with HIV must incorporate mental health considerations.

## Introduction

Worldwide, about 50 to 55% [1,2] of adults with HIV are women. Mental health is core to wellbeing [3–5]. Yet, despite PubMed citing nearly 800 peer-reviewed articles on mental health in relation to women with HIV [6], this issue is either lacking [7] or has been insufficiently addressed [8–12] in global policy guidelines.

As for all women, those with HIV experience reciprocal effects of mental health on gender-based violence (GBV) [13], their sexual and reproductive health and human rights (SRH&HR) [8] and their capacity to access and adhere to healthcare and medication, when required [14,15]. These intersecting issues are not addressed comprehensively in any one guideline. Given that psychiatric symptoms, particularly depression, and mental health vulnerabilities (e.g. maladaptive coping skills) are widespread among pregnant women living with HIV [16], it is especially concerning that mental health does not feature in global perinatal transmission guidelines [7]. Even women living with HIV, who have long recognized that HIV affects them in many different, reciprocal and gendered ways [17], have rarely addressed mental health.

The World Health Organization's (WHO) Department of Reproductive Health and Research commissioned a user-led and -designed “values and preferences” global consultation to enable policymakers to address the SRH&HR of women living with HIV as part of their guideline update process [18]. The aim of this article is to describe the extent and impact of mental health issues as experienced by women living with HIV on their SRH&HR.

## Methods

### Community consultation

A pre-survey exercise led by a global reference group (GRG) identified mental health as a key issue. A survey was developed and pilot-tested by the 14 GRG members in English with iterative feedback.

### Survey

The research involved implementation of a WHO global values and preferences survey that aimed to understand access to services and priority issues among women living with HIV. The survey was distributed in seven languages and translated back into English where necessary. All sections included open-ended responses [19]. One optional section (of eight; Table 1) explored mental health issues using simply understood common terms rather than formal instruments and inquiring when respondents had experienced these issues in relation to their HIV diagnosis [19]. Answer options were not exclusive. Women were also asked to describe in free text how these mental health disorders affected their SRH&HR and how they felt that women living with HIV could best be supported to accommodate or overcome them.

**Table 1:** Content and language dissemination of the online values and preferences survey to update the WHO SRH&HR guidelines

A. Mandatory section, including experience of accessing services, priority issues	

B. Eight optional section themes	
1	Healthy sex life
2	Pregnancy and fertility
3	Violence against women living with HIV
4	Mental health issues
5	Women living with HIV in all of our diversity
6	Puberty, menstrual issues and menopause
7	HIV treatment and side effects
8	Financial issues affecting access to services

The online survey was disseminated in English, French, Spanish, Russian, Portuguese, Bahasa Indonesian and Chinese. SRH&HR, sexual and reproductive health and human rights.

### Participants

The survey study population included any woman around the world living with HIV. The process ran from February to June 2014, building on a non-random snowball sampling model developed by the ATHENA Network [20], advertised and promoted through regional and global listservs of networks of women living with HIV and clinical networks.

### Consent

The anonymous, confidential online survey began with an explanation of the questionnaire aims and purposes, including an overview of the structure and question areas, and definitions of SRH&HR [19]. Respondents were shown the following message: “I understand that by filling in the survey, I give my consent for my responses to be used in these publications. Please click agree to be able to continue.” Respondents who did not click “I agree” were unable to proceed, and their responses were not counted. Participation was taken as implicit consent. For in-person focus groups, respondents gave explicit written or verbal consent before the discussion began. Respondents were informed that they could opt to discontinue at any time or choose not to answer any individual question.

### Ethical considerations

Institutional review board approval for this survey was not sought, after discussion with the WHO Reproductive Health and Research Department and members of the Guidelines Review Committee, as this was a consultative element of the guidelines development process. Ethical considerations were undertaken in line with the WHO 2001 *Ethical and Safety Recommendations for Research on Domestic Violence against Women* and the International Community of Women Living with HIV/AIDS 2004 guidelines on involving women living with HIV in research [21,22].

### Data collection and analyses

The qualitative and quantitative data analyzed were from the online survey. Descriptive analyses were conducted to determine frequencies, mean values and standard deviations for variables. When answers were not exclusive, frequencies were derived based on the number of respondents who had never experienced the issues or who answered “don't know.” Bivariate correlations, *t*-tests and chi-square analyses were conducted to assess associations between mental health issues, socio-demographic variables and social identities. To examine the relationship between socially disadvantaged identities (SDIs) and mental health issues of respondents, a variable named *social identity index* was created, by summing identity categories to which the respondents referred themselves (Table 2). Two identities (heterosexual; married or in a stable relationship) were not considered socially disadvantaged and thus were excluded from analyses of SDI. Multiple regression analyses were performed to investigate the associations among socio-demographic variables, SDIs and mental health issues experienced before and after (since or because of) HIV diagnosis.

**Table 2:** Respondents’ demographic information

Variables	Valid sample (*n*=766)	With an answer(s) in the mental health section (*n*=489)	Without an answer(s) in the mental health section (*n*=278)	Difference tests
Age				
15 to 24	162 (21.4%)	95 (19.4%)	67 (24.1%)	
25 to 34	288 (37.6%)	173 (35.4%)	115 (41.4%)	
35 to 44	211 (27.5%)	150 (30.7%)	62 (22.3%)	
45 to 54	97 (12.7%)	66 (13.5%)	31 (11.2%)	
55 to 64	8 (1%)	5 (1%)	3 (1.1%)	
Mean age (SD)	32.98 (9.61)	33.64 (0.43)	31.81 (0.58)	*t*=2.55*
Language of respondents				
English	385 (50.3%)	258 (52.8%)	127 (45.7%)	*χ* ^2^=20.92**
Indonesian	19 (2.5%)	11 (2.2%)	8 (2.9%)	NS
Chinese	44 (5.7%)	16 (3.3%)	28 (10.1%)	NS
Portuguese	20 (2.6%)	11 (2.2%)	10 (3.6%)	NS
French	36 (4.7%)	22 (4.5%)	14 (5.0%)	NS
Spanish	86 (11.2%)	63 (12.9%)	23 (8.3%)	NS
Russian	176 (23.0%)	108 (22.1%)	68 (24.5)	NS
Social identity index				
1. I do or have done sex work.	101 (13.2%)	66 (13.5%)	35 (12.6%)	NS
2. I inject/use or have injected/used drugs.	136 (17.7%)	102 (20.9%)	34 (12.2)	*χ* ^2^=8.92**
3. My sexual partner(s) injects/uses or has injected/used drugs.	164 (21.4%)	107 (21.9%)	57 (20.5%)	NS
4. I am a client of opioid substitution therapy programme.	26 (3.4%)	20 (4.1%)	6 (2.2%)	NS
5. I am/have been in prison.	50 (6.5%)	35 (7.2%)	15 (5.4%)	NS
6. I am/have been in a detention centre.	52 (6.8%)	32 (6.5%)	20 (7.2%)	NS
7. I am living with one or more disabilities.	101 (13.2%)	77 (15.7%)	24 (8.6%)	*χ* ^2^=7.75**
8. I have or have had active TB.	99 (12.9%)	69 (14.1%)	30 (10.8%)	NS
9. I have or have had hepatitis C.	157 (20.5%)	107 (21.9%)	50 (18.0%)	NS
10. I have or have had malaria.	119 (15.5%)	96 (19.6%)	23 (8.3%)	*χ* ^2^=17.30***
11. I migrated from one country to another for economic reasons.	59 (7.7%)	45 (9.2%)	14 (5.0%)	*χ* ^2^=4.28*
12. I migrated from one country to another for political reasons.	17 (2.2%)	8 (1.6%)	9 (3.2%)	NS
13. I am lesbian, bisexual or have sex with women.	37 (4.8%)	20 (4.1%)	17 (6.1%)	NS
14. I am a trans woman.	29 (3.8%)	17 (3.5%)	12 (4.3%)	NS
15. I am a heterosexual woman.	462 (60.2%)	299 (61.1%)	163 (58.6%)	NS
16. I am married or in a stable relationship.	361 (47.1%)	229 (46.8%)	133 (47.8%)	NS
17. I am intersex.	4 (0.5%)	1 (0.2%)	3 (1.1%)	NS
18. I have experienced any form of female genital cutting or mutilation, including circumcision.	39 (5.1%)	28 (5.7%)	11 (4.0%)	NS
19. I am or have been homeless.	78 (10.2%)	55 (11.2%)	23 (8.3%)	NS
20. I am an indigenous woman.	62 (8.1%)	51 (11.4%)	11 (4.0%)	*χ* ^2^=9.92**

NS, not significant; TB, tuberculosis;

* *p*<0.05,

** *p*<0.01,

*** *p*<0.001;

*χ* ^2^, chi-squared; *t*, student *t*-test.

A mental health outcome index was created by summing the mental health issues that respondents reported experiencing. The options “since” and “because of my diagnosis” were combined into one variable to indicate the occurrence of a mental health issue following HIV diagnosis. Respondents who reported experiences both before *and* after acquiring HIV were categorized as experiencing “persistent” mental health issues.

Thematic analysis of open-ended survey responses was performed by two social science investigators (LO, CL) [23]. Transcripts were read several times and investigators made note of initial thoughts and ideas in the data; first-level codes were then developed by highlighting relevant quotes about key areas of interest (e.g. depression). Codes were collated across data sets to produce themes that highlighted similar experiences among respondents (e.g. stigma and mental health). Themes were then structured into a thematic map (e.g. the convergence of stigma and mental health issues to compromise sexual and reproductive health (SRH)) and refined (e.g. recommendations created for supporting the mental health of women living with HIV across the micro-, meso- and macro-levels).

## Results

A total of 1173 respondents were recorded on the online survey system, but 341 did not meet the criteria of giving consent and confirming HIV status. Thus there were 832 online respondents from 94 countries.

### Quantitative findings

As 66 of 832 respondents did not give their age, the final sample for quantitative analysis was 766. A further 278 (36.3%) respondents did not answer any questions in the optional mental health section so were not entered into subsequent analyses, resulting in a sample of *n*=489 for correlation and regression analyses. The early drop-outs were found to be younger (*t*=2.55, *p*<0.05) and less likely to speak English (*χ* ^2^=20.92, *p*<0.01), use drugs (*χ* ^2^=8.92, *p*<0.01), have a disability (*χ* ^2^=7.75, *p*<0.01), have had malaria (*χ* ^2^=17.30, *p*<0.001) or be economic immigrants (*χ* ^2^=4.28, *p*<0.05). Respondents’ information on age, language and social identities are presented in Table 2.

Respondents’ experiences of mental health issues are shown in Table 3. Approximately one-fifth had experienced depression (23.9%), low self-esteem (22.5%), body image issues (18.5%), feelings of rejection (18.1%) and substance use (22%) before receiving their HIV diagnosis. Paranoia (5.0%) and anorexia (5.5%) were relatively uncommon. HIV diagnosis was identified as a critical point for mental health issues, where around 70% of respondents reported experiences of depression (74.0%), shame (70.8%), self-blame (70.0%), feelings of rejection (69.9%) and insomnia (68.4%), among others. Compared to the rates before HIV diagnosis, problems occurring “since/because of” HIV diagnosis showed considerable increases across all mental health issues, ranging from 1.2-fold (substance use) to 6.7-fold (paranoia). Analyses indicate that the average number of mental health issues experienced after HIV diagnosis (8.71, SD 4.74) is significantly greater than that before HIV diagnosis (2.48, SD 3.89), *t*(488)=23.00, *p*<0.001.

**Table 3:** Incidence of mental health issues

	Before my HIV diagnosis, *n* (%)	After my HIV diagnosis (since/because of), *n* (%)	Extent of increase (fold)	Persistent (before and after), *n* (%)	Never, *n* (%)	Don't know, *n* (%)	Total number^a^
Depression	116 (23.9)	360 (74.0)	3.10	70 (14.4)	68 (14.0)	14 (2.9)	486
Shame	86 (17.7)	325 (70.8)	3.78	46 (10.0)	96 (20.9)	8 (1.7)	459
Self-blame	69 (14.4)	334 (70.0)	4.84	44 (9.2)	104 (21.8)	15 (3.1)	478
Very low self-esteem	107 (22.5)	292 (61.5)	2.73	57 (12)	113 (23.8)	21 (4.4)	475
Feelings of rejection	85 (18.1)	327 (69.9)	3.85	49 (10.5)	87 (18.6)	18 (3.8)	468
Body image issues	86 (18.5)	263 (56.4)	3.06	42 (9.0)	126 (27.0)	36 (7.7)	466
A strong sense of isolation	54 (11.4)	253 (53.4)	4.69	23 (4.9)	170 (35.9)	22 (4.6)	474
Paranoia	23 (5.0)	154 (33.2)	6.70	12 (2.6)	241 (51.9)	62 (13.4)	464
Anxiety	64 (13.5)	314 (66.1)	4.91	38 (8.0)	118 (24.8)	19 (4.0)	475
Insomnia	63 (13.7)	314 (68.4)	4.98	34 (7.4)	96 (20.9)	23 (5.0)	459
Anorexia	25 (5.5)	140 (30.6)	5.6	7 (1.5)	272 (59.5)	28 (6.1)	457
Difficulty going out and socializing	49 (10.4)	223 (47.4)	4.55	28 (6.0)	215 (45.7)	12 (2.6)	470
Spiritual isolation	50 (10.8)	176 (37.9)	3.52	18 (3.9)	228 (49.1)	30 (6.5)	464
Loneliness	87 (18.4)	302 (65.1)	3.47	47 (10.0)	115 (24.3)	17 (3.6)	473
Suicidal feelings	75 (15.9)	177 (37.4)	2.36	37 (7.8)	228 (48.3)	25 (5.3)	472
Post-traumatic stress disorder	69 (14.6)	177 (37.4)	2.57	36 (7.6)	182 (38.4)	33 (7.0)	474
Harmful use of drugs and/or alcohol	104 (22.0)	125 (28.5)	1.20	39 (8.3)	243 (51.5)	32 (6.8)	472

a Total *n* represents the respondents who gave an answer to each respective mental health issue question. Due to the multiple-choice design, the total number of respondents for each item does not amount to the summation of options.

As indicated in Table 2, one-third (*n*=158; 32.3%) of the respondents did not identify themselves with any SDI other than HIV. One-fifth (*n*=107; 21.9%) acknowledged one SDI (in addition to HIV). Nearly half of respondents (*n*=224; 45.8%) had multiple SDIs. Three variables, experiencing mental health issues *before*, *after* and *persistently* (both before and after HIV diagnosis), were created to understand the rate, types and changes in mental health among respondents. Table 4 shows Pearson's correlation coefficient results indicating that the more SDIs a woman living with HIV reported, the more mental health issues she would experience before diagnosis (*r*=0.19, *p*<0.01), after (*r*=0.13, *p*<0.01) and persistently (*r*=0.14, *p*<0.01).

**Table 4:** Bivariate correlations between SDIs and mental health issues with time of HIV diagnosis (*n*=489)

	SDI	Mental health issues before HIV diagnosis	Mental health issues after HIV diagnosis (since/because of)	Persistent mental health issues (before and after) diagnosis
SDI	–	0.19*	0.13*	0.14*
Mental health issues before HIV		–	0.05	0.77*
Mental health issues after HIV (since/because of)			–	0.33*
Persistent mental health issues (before and after diagnosis)				–

SDIs, socially disadvantaged identities;

* *p*<0.01.

Multiple regression analyses were conducted to examine whether socio-demographic variables (age) or SDIs were significant predictors of experiencing mental health issues before and after HIV diagnosis. Regression results are presented in Table 5. The results showed that older women with more SDIs were more likely to experience mental health issues prior to their HIV diagnosis (*F*[2,486]=11.96, *p*<0.001). In addition, women with more SDIs were more likely to report mental health issues after their HIV diagnosis (*F*=2.95 [3, 485], *p*<0.05).

**Table 5:** Multiple regression analyses of correlates of experiencing mental health issues among women living with HIV (*n*=489)

Variables	Model 1 Mental health issues before HIV diagnosis	Model 2 Mental health issues after HIV diagnosis (since/because of)
Age	0.11*	0.04
SDI	0.20***	0.13**
*R* ^2^	0.04	0.01
*F*	11.96***	2.95*

SDI, socially disadvantaged identity;

* *p*<0.05;

** *p*<0.01;

*** *p*<0.001.

### Qualitative findings

#### Stigma and mental health

While a significant proportion of respondents experienced mental health issues pre-diagnosis, women indicated that HIV diagnosis is, in itself, a flashpoint for immediate and ongoing mental health problems, especially depression, feelings of rejection and insomnia.

##### Depression

Most mental health survey respondents (82%) reported experiencing depression and depressive symptoms, often following an HIV diagnosis:

It took me well over two years to recover mentally from my diagnosis. I was withdrawn, depressed and at times suicidal. There was a lack of adequate psychological and community support services. My family and friends helped me through the worst, but I'm lucky that I have good family and friends. Many others would be totally isolated. (Ireland)

HIV-related stigma contributed to fear about disclosing to friends, which played a role in enhancing depression: “The high stigma attached to HIV infection means that I have felt very lonely – unable to fully confide in friends … This also contributed to my depression” (UK).

##### Rejection and social exclusion

Over three-quarters (78%) of survey respondents reported experiencing rejection. Respondents discussed both experiences and fears of being rejected because of HIV-related stigma. This factor was a barrier to disclosure: “I live a life of fear of disclosure and secrecy with few knowing my status and little good support” (Ireland).

This point underscores linkages between lack of disclosure and lower social support.

Respondents also discussed rejection from friends: “I have lost friends, have a hugely restricted social circle and friends from before I was diagnosed with HIV” (UK).

In other situations, family members enacted stigma:

Family member[s are] blaming me for [the] death of my husband, [I] fear [to] disclose [my status] to even my mother because she will isolate me from my siblings and use me as an example in every case. (Uganda)

Another narrative highlights coping strategies, such as exercise, employed to manage isolation:

I am again feeling very sad and [have] become a recluse again. I think HIV is a very sad and isolating disease. For all of that I will train and complete a half marathon and just will not give up. (New Zealand)

##### Sleep problems

Most survey respondents (69 to 75%) reported having experienced sleep problems and discussed many root causes, including nightmares: “During [the] night you dream things which at times if you wake up – you fear even to go out or you think maybe you can die” (Tanzania).

For some women, nightmares were associated with past trauma:

I have PTSD as a result of multiple traumas. Don't get enough sleep because I'm afraid of getting nightmares. I do fine in a crisis but after everyone is safe I shut down and don't function well. (US)

Another respondent, whose concerns for her child were an extra source of stress, also discussed this narrative of functioning all day, but having challenges sleeping at night:

I do not sleep, all sorts of thoughts go through my head, mostly related to my health. I feel sorry for myself, and for my son. He will be left alone; who will support him? And if during the day I almost never think about it, [because] I'm busy all the time, at night I cannot calm down and often cry. (Ukraine)

##### Intersectional stigma

Respondents discussed the convergence of HIV-related stigma with the stigmas surrounding other factors in their life, including those related to mental health, drug use and transgender identity. A respondent articulated how fear of disclosure due to mental health stigma can exacerbate social isolation:

Mental health problems are looked upon in a bad way by society, [they are] perceived as something that should be hidden, and most often those that struggle with mental health issues are reluctant to share with anyone resulting in further isolation. (Canada)

This was reinforced by another respondent, who described stigmas converging with sexism: “There is also stigma around mental health and it compounds HIV stigma and other forms of misogyny” (Italy).

Others discussed stigma targeting people who use drugs:

Women who use drugs are subjected to double or sometimes triple stigma. There are cases of discrimination against these women, even in [the] HIV-service organizations in which they work. (Ukraine)

Transgender stigma also emerged in service provision and laws:

Think of any service a youth could benefit from and just know that trans kids probably aren't allowed to access it. (US)

The Anti Homosexuality Act has affected trans women from getting friendly services. (Uganda)

#### Stigma, mental health issues and SRH

Respondents discussed the stigma and mental health issues that present challenges for realizing SRH. Stigma and mental health issues converged to create challenges with attaining SRH&HR in three areas: sexual and intimate relationships; subtance use and its connection with sexual risk and stigma; and, human rights (HR) violations.

##### Challenges with sexual and intimate relationships

Respondents discussed HIV-related stigma and mental health issues presenting challenges both in engaging and asserting agency and control within sexual/intimate relationships. Shame and fear were described as barriers:

I have stopped engaging in sexual relationships since being diagnosed. I feel embarrassed [and] will never disclose to anyone. (Nigeria)

The issues around dating and having to talk to your lover or partner about your status at times brings anxiety, fear and depression of being rejected. (Kenya)

As a woman explained: “It makes me less assertive and I sometimes give permission to my partner to take advantage of me by having sex when I would rather not” (Nigeria).

Another discussed a result as “little or no condom negotiation” (Jamaica).

Low self-esteem also influenced sexual relationships. A respondent articulated how low self-esteem and violence could reduce the desire to be in a relationship:

I had felt very low energy level[s] at times when my self-esteem was low, whenever I experienced violence within a marital relationship and didn't feel like getting into any relationship[s] with any men for some period in my life. (India)

By contrast, a respondent discussed how low self-esteem could result in wanting to be in a sexual relationship:

When your self-esteem is low it is very difficult to have sex because you really want to have it. You have sex because you want to be accepted, you are lonely, you just want somebody to touch you … not because you really want to. (Italy)

##### Substance use and sexual risk

Substance use was often connected to coping with mental health challenges and/or socializing. This substance use in turn was discussed in relation to sexual risk in relationships and in sex work. A respondent discussed how substance use was a way of coping with low self-esteem but led to elevated sexual risks and presented a barrier to developing relationships:

Low mood, anxiety, lack of self-esteem generally combined with enjoying alcohol with socializing (and drinking a lot when I do) resulted in putting myself at risk for HIV and hence acquisition. Ongoing low mood, anxiety etc., worsened by HIV diagnosis, encourages drinking a lot (socially) and preventing formation of close relationships. (UK)

##### Reproductive health barriers and HR violations

Respondents discussed many reproductive health barriers and HR violations, including being dissuaded from having children, mistreatment while pregnant and forced or coerced sterilization. Women who wanted to have children were often constructed as wanting to transmit the virus:

In Gabon, it is a problem to decide to have children. Women [living with HIV] who choose to do it without their doctor's advice are perceived as women who wish to transmit HIV. (Gabon)

This construction of women living with HIV as wanting to transmit the virus through childbirth resulted in some respondents unwillingly choosing not to have children:

When I found out I was HIV positive, my doctor at Planned Parenthood told me I could never have children. That I might infect them and I would be [a] “horrible woman” to do so. I didn't have children but I have regretted that decision every day of my life since. I did refuse sterilization when it was “encouraged” but still wish I had considered having children as a possibility. (US)

Some women also reported coerced and forced sterilization, which can have a profound and devastating impact on a woman's mental health:

Find a law that strongly punishes doctors performing forced sterilization and that does not expire, and [allows] time to make a lawsuit if necessary because many times we realize that we were sterilized many years later and we can do nothing. (Puerto Rico)

Others discussed childbirth mistreatment, which could prevent efforts to reduce vertical transmission:

The moment a woman identifies herself as living positively with HIV, they are neglected, especially during delivery, hence [there is an] increased number of children born with HIV because women prefer to keep it a secret and be treated like the rest. (Uganda)

#### Respondents’ recommendations

Women living with HIV recommended psychological support and counselling, peer support, challenging stigma (which is one form of GBV) and promoting HR.

##### Psychological support and counselling

Respondents recommended psychological support and counselling that was affordable, accessible, holistic and integrated. Some called for future-oriented services:

Access and encouragement to use professional mental health services. Psychologists, counsellors etc. who also have [an] understanding of the need to build a life based on well being and acceptance not over focusing on problems of the past; solution building. (UK)

Many respondents recommended integrating HIV services into a one-stop shop and that counselling should include substance use and other issues: “treat underlying causes. I was an addict & couldn't get better till I gave up all drugs. HIV was a cause/symptom of that rather than the problem” (UK).

Respondents reinforced the importance of trained, skilled provision of integrated mental health and substance use services and highlighted the need to address trauma, including “resources that actually name and explain deep rooted subconscious responses and behaviours (that are normal). Post-traumatic stress counselling” (Australia).

Underscored across many narratives was the need to increase “access to mental health services for low income women” (US), preferably without payment.

##### Peer support

Peer support was discussed as highly important in helping respondents manage mental health issues: “Connecting with other positive women was the best thing I ever did for myself” (US).

Peer support groups and networks provide: “women ‘spaces’ to be themselves, to talk about what they are going through without judgment” (UK).

This support provided hope:

Peer mentoring can be helpful. Knowing that there is life after HIV is difficult at the start of a diagnosis and anyone that can show you that there is the possibility to survive and thrive is important. (Ireland)

It was also a source of learning coping and health strategies:

Joining support groups or forums where one could meet with other infected women and learn from each other as well as how others are living with and managing their conditions/lives and medication. (Zimbabwe)

##### Challenging stigma and promoting HR

Regarding mental health experiences, women wanted policymakers “not to stigmatize women living with HIV” (Uganda) and to “end the stigma by all means possible! Policies, laws, education, public information etc. What other way is there?” (UK)

Others discussed the need to tackle “the stigma related to mental health and as well as some social norms that make mental health taboo” (Uganda).

Challenging stigma and promoting HR required policies: “real health policies addressing HIV+ women and girls issues respecting their dignity and human rights” (Brazil).

## Discussion

This largest-ever survey of women living with HIV globally examined their mental health, across the complexity of their lives and in relation to SRH&HR, GBV and treatment access combined. Women have substantial mental health issues before HIV diagnosis but experience a significant manyfold increase in the incidence of mental health issues after HIV diagnosis. Seven out of ten women report depression, shame, self-blame, feelings of rejection and insomnia, and over 50% have multiple issues. These are linked with stigma/social marginalization based on intersecting identities. SDI was a significant predictor of mental health issues before and after HIV diagnosis. This point shows the importance of examining the intersecting identities of women living with HIV and strongly corroborates the qualitative findings of intersectional stigma. Stigma (itself a form of GBV [24–27]) that targets women's identities is a key reason for the increased mental health issues post-diagnosis, highlighting the role of social and structural factors in influencing the mental health of women living with HIV.

A key strength of this original user-led and -defined study is that it examines women's perspectives of mental health before and after diagnosis with a global population. The instrument and the community-based participation were grounded in appreciative enquiry approaches [28], building on strategies and practices that work for women living with HIV. Participation was global, including women from a diverse range of ages, stages, countries and contexts, although this was not a representative sample by geography. The mixed methodology gives a comprehensive view of women's challenges in negotiating life beyond an HIV diagnosis. Due to the study design, respondents may be more connected to activist and resilient networks. They had to be mentally well enough to participate: some even reported finding it cathartic and healing. Others may have found its length and the in-depth nature of questioning too hard. Only half responded to the optional section but non-responders did not appear to have more SDIs, supporting generalizability. Although not intended as a representative sample, quantitative results thus contain selection biases and imprecision. Participation was also limited by language, Internet access and connection to networks of women living with HIV. Those in greater isolation, poverty or specific contexts may be experiencing yet greater mental health challenges, for example in areas recently affected by conflict.

In order to increase access to the survey, nine focus group discussions (FGDs) in seven countries (Ethiopia (two surveys), Jamaica, Myanmar, Nepal, Senegal (two surveys), Thailand and the United Kingdom), using five further languages (Amharic, Burmese, Nepali, Thai, Wolof), ran parallel to the online survey, using the same questionnaire (data not shown). These FGDs reached out to other women living with HIV without computers, or with limited literacy or no knowledge of the online languages used. They were facilitated by GRG members or by other women well trusted by the participants. Data analysis did not produce significant differences in responses between online survey respondents and FGD participants. This result supports the content validity of the online survey findings, since findings were not just from a relatively elite group of women with online access, but also applicable to the FGD participants.

The results are comparable with reported mental health prevalence levels of people with HIV, although only one-third of studies of interventions have included women [15]. The results are cause for concern, in terms of the complexity of these findings: the burden of HIV disease alone, conflated with the known reciprocal links between mental health, SRH&HR [8], GBV [13] and treatment access [8,14,15].

The findings indicate that clinicians and global policymakers must address the complex reciprocal effects of mental health on the lives of women living with HIV in relation to their experiences of GBV, their ability to realize their SRH&HR and their capacity to access and adhere to treatment, as a matter of urgency. Moreover, root causes of mental health issues among women living with HIV such as stigma (one pervasive form of GBV) and social marginalization need urgent attention. Given widespread HIV testing and diagnosis during pregnancy [16], itself a time of heightened potential vulnerability, and recognizing diagnosis as a critical point for mental health issues, it is especially important that future global policy guidelines regarding perinatal transmission address mental health [29].

Women living with HIV recommend that their mental health issues be addressed urgently and comprehensively in policy guidelines, in training health workers and in providing relevant services [30,31].

### Implications for research

Further research is required into the following areas: exploration into and understanding of mental health issues as a contributing factor to HIV acquisition and as a consequence of HIV diagnosis (during and outside of pregnancy and childbirth); implementation of mental health strategies in sexual health services, GBV reduction services, perinatal care and in HIV care, support and treatment; mental health issues as a significant barrier to accessing all these areas. Peer-led strategies and the effectiveness of practices that many women living with HIV find of value to manage mental health, without use of (or in combination with) relevant medication, should be tested. Links for women between conflict, mental health and HIV remain inadequately explored.

## Conclusions

Mental health challenges for women living with HIV reciprocally affect their experiences of GBV, SRH&HR and treatment access. Women living with HIV who have other SDIs can be especially affected by mental health issues. If women's mental health issues are resolved it will benefit their SRH and HR. The limited attention given in global policy guidelines to the mental health of women living with HIV as a consequence of their diagnosis is an oversight that must be addressed. Furthermore, while policymakers and clinicians clearly have a role to play, the state also needs to act more broadly and cross-sectorally to address mental health issues and social disadvantage through supportive laws and policies.

## References

[1] Murray C, Naghavi M, Wang H, Lozano R (2015). Global, regional, and national age-sex specific all-cause and cause-specific mortality for 240 causes of death, 1990–2013: a systematic analysis for the Global Burden of Disease Study 2013. Lancet.

[2] (2013). Responding to intimate partner violence and sexual violence against women. WHO clinical and policy guidelines [Internet].

[3] (1946).

[4] Kamen C, Arganbright J, Kienitz E, Weller M, Khaylis A, Shenkman T (2015). HIV-related stigma: implications for symptoms of anxiety and depression among Malawian women. Afr J AIDS Res.

[5] Prince M, Patel V, Saxena S, Maj M, Maselko J, Phillips M (2007). No health without mental health. Lancet.

[6] PUBMED US National Library of Medicine National Institutes of Health [Internet].

[7] UNAIDS (2011). Countdown to zero: global plan towards the elimination of new HIV infections among children by 2015 and keeping their mothers alive.

[8] (2006). Sexual and reproductive health of women living with HIV/AIDS: guidelines on care, treatment and support for women living with HIV and their children in resource-constrained settings [Internet].

[9] (2013). Consolidated guidelines on the use of antiretroviral drugs for treating and preventing HIV infection recommendations for a public health approach.

[10] (2015). Guideline on when to start antiretroviral therapy and on pre-exposure prophylaxis for HIV [Internet].

[11] (2010). mhGAP intervention guide for mental, neurological and substance use disorders in nonspecialized health settings [Internet].

[12] (2014). Integrating the response to mental disorders and other chronic diseases in health care systems [Internet].

[13] Orza L, Bewley S, Chung C, Crone ET, Nagadya H, Vazquez M (2015). “Violence. Enough already”: Findings from a global participatory survey among women living with HIV. http://dx.doi.org/10.7448/IAS.18.6.20285.

[14] Hatcher A, Smout E, Turan J, Christofides N, Stoeckl H (2015). Intimate partner violence and engagement in HIV care and treatment among women. AIDS.

[15] Sherr L, Clucas C, Harding R, Sibley E, Catalan J (2011). HIV and depression – a systematic review of interventions. Psychol Health Med.

[16] Kapetanovic S, Dass-Brailsford P, Nora D, Talisman N (2014). Mental health of HIV-seropositive women during pregnancy and postpartum period: a comprehensive literature review. AIDS Behav.

[17] O'Sullivan S, Thomson K (1996). Positively women.

[18] Orza L, Welbourn A, Bewley S, Crone ET, Vazquez M (2015). Building a safe house on firm ground: key findings from a global values and preferences survey regarding the sexual and reproductive health and human rights of women living with HIV [Internet].

[19] (2014). Salamander Trust values and preferences survey (Questionnaire): sexual and reproductive health and human rights for women living with HIV [Internet].

[20] (2011). In women's words: HIV priorities for positive change [Internet].

[21] (2001). Putting women first: ethical and safety recommendations for research on domestic violence against women [Internet].

[22] (2004). Guidelines on ethical participatory research with HIV positive women [Internet].

[23] Braun V, Clarke V (2006). Using thematic analysis in psychology. Qual Res Psychol.

[24] Hale F, Vazquez M (2011). Violence against women living with HIV/AIDS: a background paper [Internet].

[25] (2013). 16 ideas for addressing violence against women in the context of the HIV epidemic: a programming tool [Internet].

[26] Galtung J (1969). Violence, peace, and peace research. J Peace Res.

[27] Farmer P, Nizeye B, Stulac S, Keshavjee S (2006). Structural violence and clinical medicine. PLoS Med.

[28] McAdam E, Lang P (2009). Appreciative work in schools.

[29] Stringer EM, Meltzer-Brody S, Kasaro M, Stuebe AM, Wiegand S, Paul R (2014). Depression, pregnancy, and HIV: the case to strengthen mental health services for pregnant and post-partum women in sub-Saharan Africa. Lancet Psychiatry.

[30] Logie C, James L, Tharao W, Loutfy M (2013). Associations between HIV-related stigma, racial discrimination, gender discrimination, and depression among HIV-positive African, Caribbean, and Black Women in Ontario. Canada. AIDS Patient Care STDs.

[31] Collins PY, Holman AR, Freeman AC, Patel V (2006). What is the relevance of mental health to HIV/AIDS care and treatment programs in developing countries? A systematic review. AIDS.

# Sub article: “Violence. Enough already”: findings from a global participatory survey among women living with HIV

**DOI:** 10.7448/IAS.18.6.20285

**Published:** 2015-12-01

**Authors:** Luisa Orza, Susan Bewley, Cecilia Chung, E Tyler Crone, Hajjarah Nagadya, Marijo Vazquez, Alice Welbourn

**Affiliations:** 1 Salamander Trust, London, UK; 2 ATHENA Network, London, UK; 3 Women's Health Academic Centre, King's College London, London, UK; 4 Transgender Law Center, Oakland, CA, USA; 5 ATHENA Network, Seattle, WA, USA; 6 International Community of Women Living with HIV and AIDS (ICW) East Africa, Kampala, Uganda

**Keywords:** HIV, women, human rights, gender-based violence, values and preferences, survey, intimate partner violence, evidence base

## Abstract

**Introduction:**

Women living with HIV are vulnerable to gender-based violence (GBV) before and after diagnosis, in multiple settings. This study's aim was to explore how GBV is experienced by women living with HIV, how this affects women's sexual and reproductive health (SRH) and human rights (HR), and the implications for policymakers.

**Methods:**

A community-based, participatory, user-led, mixed-methods study was conducted, with women living with HIV from key affected populations. Simple descriptive frequencies were used for quantitative data. Thematic coding of open qualitative responses was performed and validated with key respondents.

**Results:**

In total, 945 women living with HIV from 94 countries participated in the study. Eighty-nine percent of 480 respondents to an optional section on GBV reported having experienced or feared violence, either before, since and/or because of their HIV diagnosis. GBV reporting was higher after HIV diagnosis (intimate partner, family/neighbours, community and health settings). Women described a complex and iterative relationship between GBV and HIV occurring throughout their lives, including breaches of confidentiality and lack of SRH choice in healthcare settings, forced/coerced treatments, HR abuses, moralistic and judgemental attitudes (including towards women from key populations), and fear of losing child custody. Respondents recommended healthcare practitioners and policymakers address stigma and discrimination, training, awareness-raising, and HR abuses in healthcare settings.

**Conclusions:**

Respondents reported increased GBV with partners and in families, communities and healthcare settings after their HIV diagnosis and across the life-cycle. Measures of GBV must be sought and monitored, particularly within healthcare settings that should be safe. Respondents offered policymakers a comprehensive range of recommendations to achieve their SRH and HR goals. Global guidance documents and policies are more likely to succeed for the end-users if lived experiences are used.

## Introduction

Evidence over the past 15 years, documented in peer-reviewed and informal literature, shows that women living with HIV globally experience high levels of gender-based violence (GBV) and human rights (HR) abuses, including structural violence [1–20]. Intimate partner violence (IPV), and violence against women, as well as stigma and discrimination, can be viewed as overlapping sub-sets of GBV [7,15]. There are varied definitions of GBV [7,15,21,22], with different combinations of physical, sexual, psychological, financial, legal or structural abuses. Only one, from a key 2011 document aiming to start documenting the forms and extent of violence they experience, relates specifically to women living with HIV: “violence against positive women is any act, structure or process in which power is exerted in such a way as to cause physical, sexual, psychological, financial or legal harm to women living with HIV” [15].

### GBV before HIV diagnosis

IPV is already recognized to increase during pregnancy for some women regardless of HIV status: this also increases risk of poor pregnancy outcomes [23]. The World Health Organization (WHO) and others [8,10] document how IPV can increase women's vulnerability to acquiring HIV. For example, forced sex, limited or compromised negotiation about safer sex and consequent increased sexual risk-taking can all result in women acquiring HIV [4]. Violence-related mental health problems can also result in HIV acquisition and progression [4].

### GBV after HIV diagnosis

Global HIV policies regarding women with HIV have remained largely silent on GBV experienced after HIV diagnosis [24–28]. Yet IPV can affect women's ability to initiate or adhere to treatment [20]. This may have policy implications on when best to start [27,29]. Moreover, other GBV consequences, including poor mental health, poverty and lack of widow's property and inheritance rights can particularly affect capacity for women to cope with HIV, with subsequent health consequences for their children also [7].

Healthcare is recognized as a setting where professionals can support women experiencing GBV [30]. However, HR abuses against women living with HIV in healthcare settings, such as forced and coerced sterilization [31] and related psychological violence, have been documented and represent manifestations of structural and GBV [17,18,21,22].

Balancing the impact of “formal” (scholarly/peer-reviewed/replicable) and “informal” (policy/activist/community-based) evidence is challenging, but there is ongoing value in ensuring the voices and experiences of women living with HIV are heard when developing policies which affect them. In 2014, the World Health Organization (WHO) commissioned a user-led and -designed global values and preferences survey of women living with HIV, to enable policymakers to update its 2006 Guidelines on the sexual and reproductive health (SRH) of women living with HIV [32]. These guidelines required updating through changing biomedical and political aspects of the HIV response, and internal WHO technical guidelines production processes [33]. WHO aimed to enhance the relevance and effectiveness of global guidelines, policies and programmes by exploring the lived experiences of women with HIV through involving and listening to women from the outset.

The aim of this article is to explore the ways women living with HIV experienced GBV. It describes how this impacted on their SRH and HR, as elicited from this community-based participatory research process, and implications of these finding for policymakers.

## Methods

### Overall survey development and dissemination

As community-based participatory research, this comprised an anonymous, confidential, mixed-methods, global, web-based values and preferences survey [34–36]. A Global Reference Group (GRG) of 14 women with diverse experiences of acquiring and living with HIV shaped the instrument using Survey Monkey, as described elsewhere [33,37]. The GRG used a written pre-survey exercise, developed by the core team, which reviewed women's lives over the lifecourse and to reflect on all facets (physical, psychological, material, sexual, spiritual) of women's lives in relation to their SRH. The results from the pre-survey exercise informed all the survey sections and questions. The survey used an appreciative inquiry approach (which focuses on the respondent as expert in creating positive, future-oriented outcomes) [38], grounded in HR [33] and sought to identify strategies which already work and can be expanded.

The resulting survey had one mandatory and eight optional sections, included quantitative and qualitative elements, was pilot-tested with iterative feedback by the GRG and disseminated in English, French, Spanish, Russian, Portuguese, Bahasa Indonesian and Chinese.

The process ran from February to June 2014, building on a non-random snowball sampling model developed by ATHENA Network [39], advertized and promoted through regional and global listservs of networks of women with HIV and clinical networks also [33]. The survey study population included any woman around the world living with HIV [37].

### Gender-based violence questions

The GRG identified GBV as a key issue. Questions on experiences of GBV (including within SRH services), gender inequality, harmful traditional/cultural practices, and violence from intimate partners/family members/community were thus included in the initial, mandatory section.

One optional section focused further on GBV [37]. This explored experiences and fear of GBV (from intimate partner, family and neighbours, wider community, health services, and prison/detention). Another question asked women to rank as “critical,” “important” or “less important” a series of policy and programme recommendations to address or prevent GBV that had been informed by the GRG regarding specific identities of women with HIV. Women were asked about their experiences of various types of GBV and when these were experienced in relation to HIV diagnosis (“before”, “since” and “because of” answer options were not mutually exclusive). Women were also offered freetext space to detail experiences of violence and how they had accessed support.

### Analysis

Simple descriptive frequencies and proportions were used for quantitative survey data. When answers were not mutually exclusive, “ever experienced” was calculated by subtraction of “never” and “don't know” from the total.

Freetext answers in all survey sections were translated back into English where necessary and reviewed for themes about experiences of GBV and for policy and programme recommendations [15]. Thematic coding of all open qualitative responses (survey freetext answers) was performed by one social scientist (LO), through manual textual analysis to identify emergent common themes, and validated with GRG members.

### Ethical considerations

Institutional review board approval for the survey was not sought, after discussion with the WHO Reproductive Health and Research Department and members of Guidelines Review Committee, as this study was a consultative element of the guidelines development process. Ethical considerations were undertaken, however, in line with the WHO 2001 *Ethical and Safety Recommendations for Research on Domestic Violence against Women* and the International Community of Women Living with HIV/AIDS 2004 guidelines on involving women with HIV in research [40,41].

Full explanations of the survey aims and purposes, including overviews of the structure and question areas, and definitions of SRH and HR, opened the survey. Respondents were required to confirm they had HIV and that they gave their consent for their responses to be used in these publications. Online respondents who did not click “I agree” could not proceed. These responses were not counted. Participation was taken as implicit consent.

## Results

### Survey participants

In total, 832 women living with HIV from 94 countries participated in an online survey [33]. Their ages ranged from 15 to 72 years, with most in their 30s (32%) and 40s (32%). Their diversity is shown in Table 1.

**Table 1:** Participant characteristics – online survey (*n*=832, 100%)

*Survey participants’ self-identification of diversity^a^*	
Heterosexual	524 (63%)
Inject/use or have injected/used drugs	116 (14%)
Do, or have done, sex work	116 (14%)
Lesbian or bisexual	46 (5.5%)
Transgender women	37 (4.5%)
Intersex	6 (0.7%)
Indigenous	67 (8%)
In stable relationships	374 (45%)
Have experienced any form of genital cutting or mutilation	42 (5%)
Have or have had active tuberculosis, malaria or hepatitis C (respectively)	108 (13%), 150 (18%), 137 (16.5%)
Have been incarcerated or detained	42 (5%)
Have migrated for economic or political reasons (respectively)	158 (19%), 21 (2.5%)
Have other disabilities	100 (12%)
Are or have been homeless	116 (14%)

a Numbers do not add up to 100% as these were optional questions.

### Quantitative responses

GBV was reported in every section of the survey [33]. In the one mandatory section, participants overwhelmingly agreed that addressing GBV in all three contexts (within SRH services; in the home and community; and harmful traditional practices) was an “absolute must” or “high priority” (555–559/589: 94–97%). Few (18–34/589: 3–6%) considered addressing GBV to be “lower priority.”

The optional GBV section was completed by over half the online survey respondents (480/832, 58%). Table 2 shows the types, frequencies and timings of violence reported. For all six categories, some respondents gave multiple answers according to when the violence had occurred. Of respondents to the optional section, 89% (422/480) had experienced at least one form of violence in any of the six categories. Only 58 respondents (11%) said they had “never” experienced any form of violence (six of these ticked “never” in all six areas and at least one other response, but were counted as not having experienced violence). 100% (57) of respondents answering this section from Eastern Europe and Central Asia experienced at least one form of violence.

**Table 2:** Categories, frequencies and timings of violence reported by women with HIV

Category of violence experienced^a^	Before HIV diagnosis *n* (%)	Since HIV diagnosis *n* (%)	Because of HIV diagnosis *n* (%)	Any experience *n* (%)	Never *n* (%)	Don't know *n* (%)	Total *n* (%)
From a sexual partner or spouse	208 (43)	80 (17)	70 (15)	282 (59)	181 (38)	17 (4)	480 (100)
From a family member/neighbours	75 (16)	80 (17)	112 (24)	215 (45)	244 (51)	16 (3)	475 (100)
In the community	76 (16)	109 (23)	146 (32)	250 (53)	196 (42)	25 (5)	471 (100)
In health settings	28 (6)	133 (28)	164 (35)	253 (53)	209 (44)	13 (3)	475 (100)
From police/military/prison or detention services	44 (9)	34 (7)	26 (6)	78 (17)	360 (77)	31 (7)	469 (100)
Fear of violence	118 (25)	136 (29)	184 (39)	322 (68)	140 (30)	11 (2)	473 (100)

a Categories in columns 2 to 4 are not mutually exclusive. Only columns 5 to 7 add up to 100% across each row.

Fear of violence was the most commonly reported form of GBV (322/473, 68.1%), but only 15 (3%) reported “fear of violence” alone. The least reported form of GBV was from police/military/prison or detention (16.6%) (Table 2). The commonest form of violence before diagnosis was IPV (43%), which was exacerbated afterwards for some. Reports of four types of GBV (from family/neighbours, community, health settings, and fear of violence), were higher “since” or “because of” HIV diagnosis than “before.”

### Qualitative responses

In the one mandatory survey section, violence emerged as a key theme in freetext responses to the question “What's the most important issue that you would like to see the WHO Guidelines address in order to make it the most useful tool for you?” Answers varied from one word, “violence” (multiple responses), to longer statements concerning ways GBV intersects with HIV:

The impact of violence and the trauma associated with being a girl/woman in many parts of the world affect every aspect of our fight against HIV – from risk to diagnosis to accessing care to managing treatment to survival. (US)

### Lifelong violence

The optional GBV survey section also revealed that violence is not a one-off occurrence, and that it cannot be easily packaged as either a cause or consequence of HIV. The freetext results indicate the wide breadth of forms of GBV and its complex and iterative relationship with HIV, occurring throughout women's lives:

Before HIV, I was victim of different types of violence (physical, psychological, financial) besides the impact my partner's alcoholism and machismo; this lead me to get several STIs, including HIV. (El Salvador)

Violence occurred both before and after my diagnosis and post diagnosis the violence was related to stigma, which in turn resulted in being discriminated against, gossiped about, treated badly by family, asked to not use utensils, etc …. (Canada)

When I was newly diagnosed and had lost about 40Kg my neighbours and members of my church choir started avoiding me and in fact disallowed their children to come to my home and my son to enter theirs. It was such a painful experience for me. (Nigeria)

### Violence within healthcare settings

HIV diagnosis and disclosure can act as specific triggers for violence. They also expose women to new settings of HIV-related violence, including within healthcare institutions, especially concerning SRH. In other survey sections, women also reported confidentiality breaches leading to involuntary disclosure (exposing women to further forms of violence from partners, family and community members): “No Confidentiality. Nurse at hospital told my whole family. Lost my family business” (Belize/US).

Also from freetext responses, women indicated that their choice whether to test for HIV, and whether, when and how to disclose their status to their partner(s) was often compromised in maternity contexts:

Service providers should stop forcing women with HIV to disclose to their husbands, they should teach on how to help them to disclose, cause if a woman doesn't disclose she is not attended to. (Uganda)

### Lack of SRH choice

Whilst many respondents reported improved SRH services over the last 20 years, many still experienced negative provider attitudes regarding fertility desires, and no access to a full choice of services relating to contraception, maternity care and obstetrics, fertility treatment and adoption. Freetext survey responses included: “After my delivery, the midwives, knowing that I was HIV positive, didn't direct me towards family planning services … according to them I no longer had the right to have children” (Cote D'Ivoire).

The moment a woman identifies herself as living positively with HIV, they are neglected especially during delivery hence increased number of children born with HIV because women prefer to keep it a secret and be treated like the rest. Others have avoided giving birth from health centers … because of negligence in those hospitals. They prefer traditional birth attendants. (Uganda)

Respondents also reported mixed experiences regarding contraceptive choice. Service providers reportedly told many only to use condoms. Others have been coerced or forced into using long-acting or permanent birth-control methods, including intra-uterine devices (IUD, coils), injectable hormonal contraceptives, or tubal ligation (sterilization). Once diagnosed, women also reported treatment refusal (especially fertility treatment) or being forced or coerced into services they did not freely choose, including abortion: “When I got pregnant with my daughter, doctors tried to convince me to abort” (Portugal).

I went to the health facility for an IUD which they did willingly but after 6 months I wanted it removed because I wanted to have a baby. They told me the family planning method I used was for 12 yrs so they gave me a hard time that I wasted their IUD which another person would have used, ‘someone who is not like me’. It hurt me so much and I left. So I gave up removing it. (Uganda)

Make sure that there is a law that can heavily penalize doctors who perform forced sterilization and more; in many cases like mine we only realize many years later and [then] you cannot do anything. (Mexico)

### Human rights abuses and diversity

HR abuses were documented throughout the survey. In the human rights section, 180/589 (30%) respondents disagreed or strongly disagreed with the statement: “I experience the same service as any other women when I go for sexual and reproductive health services.” Their freetext responses included: “When it comes to referral to reproductive sexual health, STIs etc., the staff is completely uninformed and discrimination still persists, leaving care of women living with HIV til last” (Peru).

Women often faced considerable ignorance from health providers regarding SRH rights, and judgemental attitudes. This was exacerbated for sex workers, lesbians and transgender women especially around fertility and family planning access:

Because I am a sex worker I am ignored most of the times. (Malawi)

Health providers have no experience in dealing with lesbian women especially those living with HIV. … When asked to come with my “male partner” I responded that I am lesbian. The [healthcare professional] was very much disgusted when I revealed my sexual preference. I have never attended that hospital again. (Kenya)

As a trans woman, healthcare providers do not have the correct information about my body. They are very uncomfortable dealing with a woman who has a penis. … Now I avoid such centres. (South Africa)

Being Trans and HIV+ makes getting compassionate and comprehensive care very difficult. I have been hospitalized at the University of X Hospital, Y University Hospital, and University of Z Hospital – all places made me feel extremely unsafe. (US)

Women who used drugs also reported considerable stigma, discrimination and violence, especially regarding their reproductive rights. These respondents reported high anxiety, particularly over child custody by free text: “When my mother-in-law found out about my HIV-positive status from the hospital nurses, she made every effort to take away my daughter” (Russia/Ukraine).

When I was diagnosed doctors actively discouraged me from having a baby – even if we already knew good interventions to stop vertical transmission. Maybe it was because I was an ex-drug user and also had Hepatitis C Virus … But it took me many years to even ask again if I could have children & it was too late. (Italy)

### Support for policies to address or prevent GBV

Survey respondents were asked what messages to offer policymakers, or to give recommendations on how to address and prevent violence against women with HIV:

I would like them to address gender violence because women are suffering in the hands of their husbands. Most women like me stay in the marriage not because I want but because I have nowhere else to go and also the business I do is family business so if I leave I won't have any financial support, so I endure the beatings, insults etc. because I don't have an alternative. (Kenya)

### Policy recommendations

Women's responses to the policy recommendations presented in the optional GBV section covered a wide range of issues (Table 3). Over 80% of respondents agreed that these are critical or important ways healthcare services can address violence against women with HIV [33].

**Table 3:** What women living with HIV think are the most important ways to address or prevent gender-based violence

Strategy	Critical *n* (%)	Important *n* (%)	Less important *n* (%)	Don't know *n* (%)	Total response *n* (%)
*Through safe health services that protect, respect and uphold women's rights*					
Sensitize healthcare workers to the rights of women living with HIV	363 (77)	86 (18)	11 (2)	11 (2)	471 (100)
Increase access to quality support services for women who experience gender-based violence (including sexual violence)	356 (76)	94 (20)	11 (2)	8 (2)	469 (100)
Ensure effective complaints/redress mechanisms in case of rights violations within health services	332 (71)	114 (24)	10 (2)	10 (2)	466 (100)
Provide a minimum post-rape care and support package, including post-exposure prophylaxis, emergency contraception, screening for other sexually transmitted infections, and psychosocial care/counselling	330 (71)	114 (24)	9 (2)	13 (3)	466 (100)
Increase access to harm reduction-based treatment for women who use drugs	238 (51)	178 (38)	26 (6)	23 (5)	465 (100)
Address alcohol abuse	206 (44)	182 (39)	54 (12)	22 (5)	464 (100)
*Through a protective legal and policy environment and decriminalization*					
Strengthen laws and policies to protect the rights of people living with HIV	376 (80)	79 (17)	9 (2)	8 (2)	472 (100)
Strengthen legal protections around all forms of violence against women/gender-based violence	358 (76)	90 (19)	9 (2)	14 (3)	471 (100)
Recognize and address marital rape and “date rape”	280 (60)	143 (31)	17 (4)	25 (5)	465 (100)
Remove laws which criminalize HIV exposure/transmission	235 (51)	112 (24)	63 (14)	48 (10)	458 (100)
Remove laws which criminalize same sex practices	181 (40)	131 (29)	97 (21)	48 (11)	457 (100)
Remove laws which criminalize sex work	166 (35)	153 (33)	89 (19)	61 (13)	469 (100)
Remove laws which criminalize drug use	157 (34)	148 (32)	106 (23)	54 (12)	465 (100)
*Through financial security*					
Increase social protection for women and children	333 (71)	116 (25)	12 (3)	8 (2)	469 (100)
Increase access to employment for women, including transgender women	269 (58)	153 (33)	26 (6)	17 (4)	465 (100)

In looking beyond “just” an end to GBV, further policy and practice suggestions were ranked as shown in Table 4. A range of 54–93% of respondents agreed proposed measures were “critical” or “important.” For lower percentage responses, a consistently greater proportion also answered “don't know” (up to 30%).

**Table 4:** Policy recommendations that would improve the SRH of women living with HIV in all their diversity (from optional section relating to diversity)

Policy recommendations that would improve the SRH of women living with HIV in all their diversity	Proportion endorsing recommendations as critical or important *n* (%)
*Women who use drugs*	
Access to methadone or buprenorphine for women living with HIV who inject drugs and are pregnant	250/430 (56)
Treatment and support for hepatitis C co-morbidities	398/428 (93)
Education on prevention and first-aid for overdoses, including access to naloxone	*
*Sex workers*	
Interventions to halt and address violence and discrimination against sex workers	375/425 (88)
*Lesbian, bisexual, transgender women, and other women who have sex with women*	
SRH services tailored for lesbian, bisexual, transgender women or other women living with HIV who have sex with women	288/428 (67)
Introduction of SRH guidelines/policy for transgender women	279/419 (67)
Access to sexual reassignment surgery for transgender women	234/424 (55)
Access to other gender-affirming surgeries for transgender women	226/422 (54)
*Women in prison or detention*	
Continuity of treatment access and adherence support for women in prison or detention and women re-entering into society	393/426 (92)
Addressing HIV-related stigma and discrimination among prison staff and inmates	399/428 (93)
Consistent implementation of up-to-date practice guidelines in relation to women living with HIV in prison	388/426 (91)
*Women with disabilities*	
Tailored access to information and services for women with disabilities	402/431 (93)
*For all women with HIV, throughout the life-cycle*	
Comprehensive sexuality education	403/433 (93)
Removal of age-restrictive polices	254/403 (63)
Treatment and support for TB co-morbidities	398/428 (93)

* Inadvertently not included in the survey, but later highlighted by GRG members.

One survey respondent highlighted the importance of using the guidelines in conflict-afflicted countries:

Issues around GBV and HIV/AIDS for women and girls in South Sudan. The … country continues to be troubled in political issues and insecurity, women and girls continue to suffer the brunt of it all. WHO should be specific … on how to address GBV and HIV/AIDS for women and girls in an insecure country. (South Sudan)

## Discussion

This mixed-methods study, developed and led by women living with HIV, describes the experiences of 945 participants from 94 countries globally. Eight-hundred and thirty-two women took part in an online survey, the data from which are described above. A further 113 women living with HIV from key affected populations took part in 11 focus group discussions (FGDs) in seven countries. This enabled more respondents with no limited literacy or no computer access or limited literacy to answer the same survey questions and, through discussion, to deepen understanding and triangulate findings. Data analysis did not produce significant differences in responses between online survey respondents and FGD participants, therefore supporting the content validity of the online survey findings (data not shown). It is the largest study of its kind to explore SRH and HRs of women with HIV.

Of concern is that 89% of respondents to the GBV section of the online survey reported violence. Women reported HIV-related violence before, since and because of HIV diagnosis – or a combination of all three. Fear of violence was more common than other forms of GBV, but there were high background IPV levels before and after diagnosis. Notably, higher levels of violence were experienced post-diagnosis in health settings and the community. Peer-review literature to date has not highlighted the increased levels of GBV in such settings, nor the very high proportion of respondents reporting violence at different stages of their lives. GBV was a high priority for many women with HIV, including sex workers, transgender women and women who used drugs. The range and complexity of the violence experienced by women living with HIV combined to constrain their capacity to enjoy SHR and HRs.

The strengths of this largest-ever survey of women with HIV include being user-led, with efforts made to ensure inclusion of women living with HIV in multiple contexts and diversities. It was based on a snowballing technique via informed networks, used both qualitative and quantitative methods, and achieved a large global response.

With constrained timeframe and budget, the study's key limitation is that participants do not represent all women with HIV and was biased towards women with Internet access who had an activist interest through their listserv membership. Experience of GBV might influence the wish to participate in either direction. Numbers from each of many key populations are relatively small due to the breadth of participation. It was particularly difficult to reach younger women and women living with HIV in conflict-affected situations, who may have other specific experiences of violence not captured by the survey. Nevertheless, the response exceeded expectations and achieved sufficient breadth and depth of information required to support generalizability.

The implications for clinicians and policymakers are: that stigma and discrimination against women living with HIV are forms of GBV and need to be recognized as such; and that GBV is commonly experienced by women living with HIV globally. Safety is the foundation of an effective response to violence against women in the context of HIV. WHO/UNAIDS have documented the existing evidence base for effective interventions in four strategic areas: effective laws and policies, transformative gender norms, integrated violence against women and HIV services, and empowering women and girls though integrated multi-sectoral approaches [7]. Survey respondents highlighted all these strategic areas, and also others including alcohol policies and peer-support.

Healthcare settings need to be safe places for all women including key populations [42], and there is a need for greater awareness-raising and evidence-informed debate in these areas.

Healthcare practitioners and policymakers should be trained to explore the effects of their words and actions, both on the women themselves and on their ability to care for their children. Moreover, policies related to antiretroviral initiation must consider, measure and mitigate the GBV that may follow an HIV diagnosis.

Since so many women are diagnosed with HIV during pregnancy, healthcare practitioners and policymakers need to be particularly mindful of the incremental effects of a diagnosis on GBV. Considering the reports of lack of safety and HR protection within healthcare services, HR violations including mandatory testing, disclosure, and coerced or forced sterilization and abortion should be particularly urgently addressed. Such information might be sought directly (e.g. via community surveys, non-governmental organizations, legal redress, complaints), or found indirectly (e.g. from measuring access to care and treatment, non-attendance or subsequent retention in care) [43].

Implications also include that women living with HIV need to be aware of their rights and the different forms of GBV, and that they may identify and name violence, recognize rights violations, find support and develop greater agency to seek redress and safety. In addition to the priorities identified in the survey, this study supports suggestions that all healthcare staff should also be equipped to enquire about GBV, as a part of routine, everyday, safe interactions within healthcare settings [44,45], as benefit for all women and girls, especially those with HIV. Healthcare services must also have formal pathways to advocacy and safety planning for women who disclose GBV [44,45].

Regarding future research, there is urgent need for replication, quantification, refinement and testing of interventions informed by the experiences of violence and the intersections of health and HR across the life-cycle. Research should address health, dignity, and welfare; the interface between violence and HIV exposure, acquisition and impact; the interface between violence and other factors, such as gender identity and gender expression; their impact on the SRH and HR of women living with HIV; women's capacity to take antiretrovirals – as well as their ability to act as primary carers of the next generation.

## Conclusions

Women living with HIV experience unacceptable levels of violence, including in healthcare settings, across their life-cycle. This study contributes to the growing literature on GBV in women living with HIV, both before and after diagnosis. Global policymakers should heed this literature in policy formulation and implementation. Since guidelines examine the formal evidence base which takes time to be published, any time-lag in information or implementation risks failing the very end-users that global polices seek to support in the absence of such key information. Community-based information can act as a valuable early-warning monitoring system to assess unintended consequences of global policies.

No woman, with or without HIV, should have to experience GBV, especially not in healthcare settings, over which policymakers and service providers have most control. GBV should be recognized as a barrier to effectiveness of many policies regarding testing, care, treatment initiation and adherence. Addressing GBV as a critical policy issue has instrumental as well as inherent value. Forthcoming guidelines should encompass recommendations for policy, formal healthcare, and a more holistic view of the current SRH and HR of women living with HIV in all their diversities, which include community engagement, a firm foundation of safety, and are based on a HR framework.

## References

[1] Siemieniuk R, Krentz H, Miller P, Woodman K, Ko K, Gill M (1999). The clinical implications of high rates of intimate partner violence against HIV-positive women. J Acquir Immune Defic Syndr.

[2] Gielen A, McDonnell K, Burke J, O'Campo P (2000). Women's lives after an HIV-positive diagnosis: disclosure and violence. Matern Child Health J.

[3] Zierler S, Cunningham WE, Andersen R, Shapiro MF, Nakazono T, Morton S (2000). Violence victimization after HIV infection in a US probability sample of adult patients in primary care. Am J Public Health.

[4] Campbell JC, Baty ML, Ghandour RM, Stockman JK, Francisco L, Wagman J (2008). The intersection of intimate partner violence against women and HIV/AIDS: a review. Int J Inj Contr Saf Promot.

[5] Osinde M, Kaye D, Kakaire O (2011). Intimate partner violence among women with HIV infection in rural Uganda: critical implications for policy and practice. BMC Womens Health.

[6] Dhairyawan R, Tariq S, Scourse R, Coyne K (2012). Intimate partner violence in women living with HIV attending an inner city clinic in the UK: prevalence and associated factors. HIV Med.

[7] WHO/UNAIDS (2013). 16 ideas for addressing violence against women in the context of the HIV epidemic: a programming tool [Internet].

[8] (2013). Global and regional estimates of violence against women: prevalence and health effects of intimate partner violence and non-partner sexual violence [Internet].

[9] Li Y, Marshall C, Rees H, Nunez A, Ezeanolue E, Ehiri J (2014). Intimate partner violence and HIV infection among women: a systematic review and meta-analysis. J Int AIDS Soc.

[10] Durevall D, Lindskog A (2015). Intimate partner violence and HIV in ten sub-Saharan African countries: what do the Demographic and Health Surveys tell us?. Lancet Global Health.

[11] Zunner B, Dworkin S, Neylan T, Bukusi E, Oyaro P, Cohen C (2015). HIV, violence and women: unmet mental health care needs. J Affect Disord.

[12] Olowookere S, Fawole O, Adekanle D, Adeleke N, Abioye-Kuteyi A (2015). Patterns and correlates of intimate partner violence to women living with HIV/AIDS in Osogbo, Southwest Nigeria. Violence Against Women.

[13] Feldman R, Manchester J, Maposhere C, Cornwall A, Welbourn A (2002). Positive women: voices and choices in Zimbabwe. Realizing rights.

[14] (2007). Hidden in the mealie meal: gender-based abuses and women's HIV treatment in Zambia [Internet]. Human Rights Watch.

[15] Hale F, Vazquez M (2011). Violence against women living with HIV/AIDS: a background paper [Internet].

[16] Vazquez M, Hale F (2011). Ethical considerations for an integral response to human rights, HIV and violence against women in Central America [Internet].

[17] (2012). If I knew what would happen I would have kept it to myself: gender violence and HIV [Internet].

[18] Paxton S (2012). Positive and pregnant: how dare you – a study on access to reproductive and maternal health care for women living with HIV in Asia [Internet].

[19] Hutchinson J, Perry G (2012). Violence as a cause and consequence of women living with HIV in England [Internet].

[20] Mwanza J (2012). Baseline report on intimate partner violence amongst people living with HIV [Internet].

[21] Galtung J (1969). Violence, peace, and peace research. J Peace Res.

[22] Farmer PE, Nizeye B, Stulac S, Keshavjee S (2006). Structural violence and clinical medicine. PLoS Med.

[23] (2011). Intimate partner violence during pregnancy: information sheet [Internet].

[24] (2011). Global plan towards the elimination of new HIV infections among children by 2015 and keeping their mothers alive 2011–2015 [Internet].

[25] Chitembo A, Dilmitis S, Edwards O, Foote C, Griffiths L, Moroz S (2012). Towards an HIV-free generation: getting to zero or getting to rights?. Reprod Health Matters J.

[26] (2013). Consolidated guidelines on the use of anti-retroviral drugs for treating and preventing HIV infection: recommendations for a public health approach [Internet].

[27] Welbourn A (2014). WHO's poor consultation with patients on HIV guidance has denied women choice in drug treatment. BMJ.

[28] (2015). Guideline on when to start antiretroviral therapy and on pre-exposure prophylaxis for HIV [Internet].

[29] AVAC, ATHENA, Salamander Trust, UNWomen (2015). Key barriers to women's access to HIV treatment: making ‘Fast-Track’ a reality [Internet].

[30] Heise L, Ellsberg M, Gottmoeller M (2002). A global overview of gender-based violence. Int J Gynecol Obstet.

[31] Southern Africa Litigation Centre (2013). African Commission on human and peoples’ rights condemns involuntary sterilisation of women living with HIV in Africa [Internet].

[32] (2006). Sexual and reproductive health of women living with HIV/AIDS: guidelines on care, treatment and support for women living with HIV and their children in resource-constrained settings [Internet].

[33] Orza L, Welbourn A, Bewley S, Crone E, Vazquez M (2015). Building a safe house on firm ground: key findings from a global values and preferences survey regarding the sexual and reproductive health and human rights of women living with HIV [Internet].

[34] Raab M, Stuppert W (2014). Review of evaluation approaches and methods for interventions related to violence against women and girls (VAWG).

[35] Creswell J, Klassen A, Clark V, Smith K NIH Office of Behavioral and Social Sciences Research (OBSSR) – mixed methods research index [Internet].

[36] Jagosh J, Bush P, Salsberg J, Macaulay A, Greenhalgh T, Wong G (2015). A realist evaluation of community-based participatory research: partnership synergy, trust building and related ripple effects. BMC Public Health.

[37] (2014). Salamander Trust Values and Preferences Survey (Questionnaire): sexual and reproductive health and human rights for women living with HIV [Internet].

[38] McAdam E, Lang P (2009). Appreciative work in schools.

[39] (2011). In women's words: HIV priorities for positive change [Internet].

[40] (2001). Putting women first: ethical and safety recommendations for research on domestic violence against women [Internet].

[41] (2004). Guidelines on ethical participatory research with HIV positive women [Internet].

[42] (2014). Consolidated guidelines on HIV prevention, diagnosis, treatment and care for key populations [Internet].

[43] Whiteford L, Vindrola-Padros C (2015). Community participatory involvement.

[44] (2013). Responding to intimate partner violence and sexual violence against women WHO clinical and policy guidelines [Internet].

[45] (2014). Domestic violence and abuse: how health services, social care and the organisations they work with can respond effectively [Internet].

# Sub article: Safer disclosure of HIV serostatus for women living with HIV who experience or fear violence: a systematic review

**DOI:** 10.7448/IAS.18.6.20292

**Published:** 2015-12-01

**Authors:** Caitlin E Kennedy, Sabina Haberlen, Avni Amin, Rachel Baggaley, Manjulaa Narasimhan

**Affiliations:** 1 Department of International Health, Johns Hopkins Bloomberg School of Public Health, Baltimore, MD, USA; 2 Department of Epidemiology, Johns Hopkins Bloomberg School of Public Health, Baltimore, MD, USA; 3 Department of Reproductive Health and Research, World Health Organization, Geneva, Switzerland; 4 Department of HIV/AIDS, World Health Organization, Geneva, Switzerland

**Keywords:** disclosure, violence, gender-based violence, review, systematic

## Abstract

**Introduction:**

Supporting individuals as they disclose their HIV serostatus may lead to a variety of individual and public health benefits. However, many women living with HIV are hesitant to disclose their HIV status due to fear of negative outcomes such as violence, abandonment, relationship dissolution and stigma.

**Methods:**

We conducted a systematic review of studies evaluating interventions to facilitate safer disclosure of HIV status for women living with HIV who experience or fear violence. Articles, conference abstracts and programme reports were included if they reported post-intervention evaluation results and were published before 1 April 2015. Searching was conducted through electronic databases for peer-reviewed articles and conference abstracts, reviewing websites of relevant organizations for grey literature, hand searching reference lists of included studies and contacting experts. Systematic methods were used for screening and data abstraction, which was conducted in duplicate. Study quality (rigor) was assessed with the Cochrane risk of bias tool.

**Results:**

Two interventions met the inclusion criteria: the Safe Homes and Respect for Everyone cluster-randomized trial of combination HIV and intimate partner violence (IPV) services in Rakai, Uganda, and the South Africa HIV/AIDS Antenatal Post-Test Support study individual randomized trial of an enhanced counselling intervention for pregnant women undergoing HIV testing and counselling. Both programmes integrated screening for IPV into HIV testing services and trained counsellors to facilitate discussions about disclosure based on a woman's risk of violence. However, both were implemented as part of multiple-component interventions, making it impossible to isolate the impact of the safer disclosure components.

**Conclusions:**

The existing evidence base for interventions to facilitate safe HIV serostatus disclosure for women who experience or fear violence is limited. Development and implementation of new approaches and rigorous evaluation of safe disclosure outcomes is needed to guide programme planners and policy makers.

## Introduction

HIV serostatus disclosure has been associated with many potential benefits. People who disclose their HIV status may receive social support and experience reduced stigma [1], which may in turn lead to other positive outcomes for them, their partners and their families, such as engagement in HIV prevention services (including prevention of mother-to-child transmission [PMTCT]) and uptake of and adherence to HIV care and treatment services [2]. Disclosure to sexual partners could also increase rates of HIV testing and reduce transmission risk behaviours [3], including increasing the use of antiretroviral treatment [4] or pre-exposure prophylaxis (PrEP) [5] to prevent HIV transmission in sero-discordant couples.

Despite these potential benefits, evidence from a number of studies and several reviews shows that a substantial number of women are hesitant to disclose their HIV status due to fear of negative outcomes such as violence, abandonment, relationship dissolution, stigma, loss of children or loss of their home [6,7]. Violence is common in the lives of women. Globally, 35% of women age 15 and older are estimated to have experienced physical or sexual violence by an intimate partner or non-partner sexual violence in their lifetime [8]. A recent survey of 832 women living with HIV from 94 countries found that participants identified violence as a significant concern related to their sexual and reproductive health [9]. Violence can be both a risk factor for and an outcome of HIV infection [8]. Women who are currently in violent relationships may be at risk of further violence that is triggered by HIV serostatus disclosure, while other women who have not previously experienced violence may newly experience it as an outcome of disclosure. A 2003 systematic review found that following HIV serostatus disclosure to a partner between 3.5 and 14.6% of women across studies reported experiencing negative reactions, including violence [6]; another non-systematic review also found that violence may occur around disclosure [10]. More recent studies from Ethiopia, Nigeria and Zimbabwe have found that large proportions of women report negative reactions, including violence, as outcomes of disclosure and that women who disclosed were at increased risk of physical and emotional violence compared to those who had not [11–13]. Further, a systematic review of studies from sub-Saharan Africa found that a history of domestic violence is associated with non-disclosure of HIV serostatus among pregnant and postpartum women [14]. Uptake of HIV testing services among women has increased, especially among pregnant women in the context of PMTCT programmes. However, fear of disclosure and experience of violence and other traumatic or stressful life events after disclosure of HIV status have been associated with lower adherence to antiretroviral treatment for women's own health and for PMTCT [15–19]. Fear and experience of violence related to HIV serostatus disclosure may also prevent women from receiving social support, accessing other reproductive health services such as postpartum care and being able to negotiate safer sex [20,21].

In 2006, the WHO issued a meeting report on addressing violence against women in HIV testing and counselling services [22]. This report called for operational research to assess counselling, communication and referral tools to support women through the disclosure and risk-reduction planning processes. The report identified multiple models for integration in this area, including in HIV testing and counselling protocols that considered risk of violence as part of efforts to evaluate the safety of HIV status disclosure. However, the report did not assess evidence of success of these programmes. To examine the evidence for such programmes nearly a decade after the release of this report, we conducted a systematic review of rigorous evaluations of interventions to facilitate safe disclosure of HIV status for women living with HIV who fear violence or are currently experiencing violence.

## Methods

We conducted a systematic review of the literature following PRISMA guidelines [23].

### Inclusion criteria

Inclusion criteria for the review were as follows:

1. Published in a peer-reviewed journal, presented as an abstract at a scientific conference or presented as a grey literature report prior to the search date of 1 April 2015
2. Comparative study (including either pre-/post- or multi-arm comparison groups) assessing one or more interventions to facilitate safe disclosure of HIV status for women living with HIV who fear violence or who disclose that they are currently experiencing violence compared with no intervention or standard of care
3. Measures one or more of the following outcomes: (1) disclosure, (2) violence (physical, sexual, emotional), (3) fear of violence, (4) other adverse events (e.g. relationship dissolution, abandonment, job loss, loss of children, loss of access to services, etc.) or (5) positive outcomes (e.g. feelings of individual empowerment, safety, partner involvement, better physical health for self and children, HIV care and treatment engagement, adherence to antiretroviral treatment, etc.)

Following Obermeyer et al. [7], we defined *HIV serostatus disclosure* as “the process of revealing a person's HIV status.” However, while Obermeyer et al.'s definition encompasses both HIV-positive and HIV-negative serostatus disclosure, we focused on disclosure outcomes among HIV-positive women where such data were available. We included studies among all populations of women living with HIV, including adolescents (10 to 19 years) and young people (20 to 24 years) [24] and women who are members of key populations (e.g. sex workers, women who use drugs, and women in prisons or other closed settings). We also included all women living with HIV, not just those who are newly diagnosed through HIV testing services.

Interventions to facilitate safer disclosure of HIV status were defined as any effort to reduce the risk of violence or other negative outcomes associated with HIV serostatus disclosure. Such efforts could include identifying people at risk of violence following disclosure, helping them find strategies to disclose in a way that reduced their risk of violence (such as through facilitated disclosure, role plays or safe disclosure plans) or helping them decide when non-disclosure was the safest option. Studies examining all types of violence were included, including physical, sexual and emotional violence, and all types of violence perpetrators, including intimate partners, family members and others. Studies could assess violence using any measure (e.g. the conflict tactic scale, WHO methodology, DHS methodology or an alternate measure). We defined current experience of violence as recent violence (in the past 12 months); however, we included articles that used any definition of current or recent experience of violence as long as it was intended to identify violence experienced after the intervention. We were particularly interested in outcome measures that captured violence related to HIV serostatus disclosure as opposed to background levels of violence, such as lifetime prevalence of violence.

No restrictions were placed based on location of the intervention. No language restrictions were used on the search; if we had identified articles published in languages other than English that met the inclusion criteria, we intended to translate them into English.

### Search strategy

We searched the following electronic databases through the cutoff date of 1 April 2015: PubMed, CINAHL (Cumulative Index to Nursing and Allied Health Literature) and EMBASE. The following terms were entered into all computer databases: (disclos*) AND (violence OR abuse OR rape OR “forced sex” OR “coerced sex”) AND (HIV OR AIDS). We also conducted secondary reference searching on all studies included in the review as well as other review articles on interventions to facilitate HIV serostatus disclosure [25] and key meeting reports [22]. We used Google Scholar to identify articles that cited the 2006 WHO meeting report “Addressing violence against women in HIV testing and counselling” [22]. Further, we contacted a small number of selected experts in the field, including the lead authors of included studies, to identify any additional studies we may have missed.

We searched for conference abstracts by searching websites for the following conferences through 1 April 2015: International AIDS Conference; IAS Conference on HIV Pathogenesis, Treatment, and Prevention; the Conference on Retroviruses and Opportunistic Infections; the International Conference on AIDS and STIs in Africa; and the Sexual Violence Research Initiative (SVRI) conference. We attempted to include abstracts from the End Violence Against Women International Annual Conference and the World Association for Sexual Health Congress, but they were not available online.

To search for other grey literature, we searched the USAID Development Experience Clearinghouse (DEC) under the terms *HIV* and *violence* and reviewed websites of the following organizations known to be involved in initiatives related to violence and HIV: SVRI, the STRIVE research consortium, London School of Hygiene and Tropical Medicine – Gender Violence and Health Centre, Futures without Violence, the Futures Group, Columbia University ICAP programme, International Planned Parenthood Federation, FHI360, MenEngage, EngenderHealth and Population Council.

### Screening abstracts

Citations identified through the search strategy underwent an initial screening by a single reviewer based on title and abstract. All citations that were considered possibly relevant were then screened by two reviewers separately to assess whether they met the inclusion criteria, with differences resolved through consensus. Full text articles were obtained of all selected abstracts and two independent reviewers made a final determination of study inclusion after full text review.

### Data extraction and analysis

Data were extracted independently by two reviewers using standardized forms. Differences in data extraction were resolved through consensus and discussion with all authors when necessary. The following information was gathered from each included study: citation information, study objectives, location, population characteristics, description of the intervention, study design, sample size, follow-up periods and loss to follow-up, analytic approach, violence measures, outcome measures, comparison groups, effect sizes, confidence intervals (CIs), significance levels, conclusions and limitations. For randomized controlled trials, risk of bias was assessed using the Cochrane Collaboration's tool for assessing risk of bias [26].

Meta-analysis was not conducted due to the small number of included studies. Instead, we present a descriptive summary of the findings across studies based on our coding categories and outcomes.

## Results

Our initial database search yielded 1080 published citations (Figure 1). In addition, over 2200 conference abstracts, unpublished reports and other grey literature publications were reviewed. The vast majority of these were excluded in the initial screening; 52 were retained for closer consideration by two independent reviewers. At this level, 45 citations were excluded for not meeting the inclusion criteria – generally because they did not report specific intervention evaluation data related to safer HIV disclosure – and five were literature reviews included as background material. We identified two studies that ultimately met the inclusion criteria. One of the studies published evaluation results in 2015 [27], and the other is completed [28] but evaluation results for the safer disclosure component have not yet been published. The lead authors of both studies were contacted to obtain additional information.

**Figure 1:** PRISMA flowchart showing disposition of citations through the search and screening process.

### Study descriptions

Table 1 presents descriptions of the included studies. Both were conducted in sub-Saharan Africa, one in rural Uganda [27] and one in urban South Africa [28]. Both integrated screening for intimate partner violence (IPV) into HIV testing and counselling and trained counsellors to facilitate discussions about disclosure based on a woman's risk of violence. In addition, both studies included multiple other intervention components designed to address community norms around IPV and HIV [27] or to enhance services for women undergoing HIV testing [28].

**Table 1:** Descriptions of evaluated interventions

Study	Setting	Population characteristics	Intervention description
Wagman et al., 2015	Uganda Rakai district	General adult population Gender: 59% female, 41% male Age range: 16 to 42 years	**Safe disclosure component:** The Safe Homes and Respect for Everyone (SHARE) project included a screening and brief intervention to reduce HIV-disclosure-related violence and sexual risk in women seeking HTC. HIV counselling protocols were modified to address IPV, and HTC counsellors were trained to screen women for IPV and handle or refer IPV cases; help HIV-positive women develop safe HIV disclosure plans; and help abused women develop safe sex negotiation skills. The intervention was designed for women newly diagnosed with HIV, but was offered to women who were diagnosed previously but had not disclosed their status and expressed fear of violence. **Other components:** SHARE also included extensive community-based mobilization to change attitudes and social norms that contribute to IPV and HIV risk.
Maman et al., 2014 and in progress	South Africa Umlazi Township, Durban	Pregnant women Gender: 100% female Mean age: 25.5 years	**Safe disclosure component:** The South Africa HIV/AIDS Antenatal Post-test Support (SAHAPS) study included an additional structured discussion tool to help women assess their risk for physical harm following disclosure in women seeking HTC. The counselling tool consisted of five questions that were meant to prompt discussion about the possibility of disclosure-related violence between the counsellors and their clients. Based on answers to these questions, counsellors were trained to explore women's risk of disclosure-related violence and potentially explore alternative options including opting not to disclose, deferring disclosure to a time when women's safety can be insured or developing a plan for mediated disclosure in which the women either brings the partner to the clinic to disclose in the presence of a counsellor or identifies a trusted family member or friend who can be present with the woman when she shares her HIV test results with her partner, or alternative options. **Other components:** SAHAPS also included enhanced HIV pre- and post-test counselling at the first antenatal visit, as well as two additional counselling sessions at 6 and 10 weeks postpartum. The postpartum counselling sessions were conducted by the same counsellors the women saw during their antenatal visit. Women also had the option to use support groups, which were ongoing through the antenatal and postpartum period, and the option to use onsite legal services.

HTC, HIV testing and counselling; IPV, intimate partner violence.

In the rural Rakai district, Uganda, the Safe Homes and Respect for Everyone (SHARE) project provided a combination of IPV prevention and HIV services from 2005 to 2009 [27]. SHARE employed two main approaches to achieve these goals: a screening and brief intervention in HIV testing and counselling to reduce HIV disclosure-related violence and sexual risk and community-based mobilization to change attitudes and social norms that contribute to IPV and HIV risk [29]. For the safer disclosure component, counsellors were trained to screen women for IPV and handle or refer IPV cases, help HIV-positive women develop safe HIV disclosure plans and help abused women develop safe sex negotiation skills. Additional community mobilization activities included advocacy with local leaders, officials and policy makers; capacity building and activism with community members and volunteers; dissemination of booklets, brochures, posters, story cards and other learning materials; special events such as fairs, marches, campaigns and poster shows; work with youth and men; and support groups for HIV-positive women. The intervention was evaluated through a cluster-randomized trial nested within the Rakai Community Cohort Study – an open, community-based cohort [27].

In Umlazi Township, Durban, South Africa, the South Africa HIV/AIDS Antenatal Post-Test Support (SAHAPS) study evaluated an enhanced counselling intervention for pregnant women undergoing HIV testing and counselling [28]. The intervention group received enhanced pre- and post-test counselling during antenatal care as well as two additional counselling sessions at 6 and 10 weeks postpartum. Women also had the option to use support groups and onsite legal services. For the safer disclosure component, counselling included a five-question structured discussion tool to help women assess their risk for physical harm following disclosure. Counsellors were then trained to explore women's risk of disclosure-related violence and to explore alternative options where indicated. The intervention was evaluated through an individual randomized controlled trial [28].

### Study results

Table 2 presents a summary of key characteristics and outcomes for the included studies. Both studies received generally high marks on the Cochrane risk of bias tool, although due to the nature of the interventions, neither study was able to blind participants or study staff to intervention allocation. Both studies examined the impact of the intervention on disclosure and violence; both measured violence using an adapted version of the conflict tactics scale. The SAHAPS study measured disclosure and violence 14 weeks after the receipt of HIV test results and excluded women who already knew they were HIV-positive, enabling measurement of violence that was proximal to disclosure. In contrast, the SHARE study measured disclosure and violence in the past year among community members residing in the intervention and control communities, including people who did not receive new HIV results during the time of the intervention. Neither study measured the effect of the intervention on fear of violence, other adverse events or positive outcomes. Neither study reported outcomes specifically among women living with HIV who were identified as experiencing or fearing violence who received the safer disclosure intervention; however, the SHARE study was able to provide unpublished data for the sub-sample of women living with HIV. Below, we present outcomes of both studies on disclosure and violence among women.

**Table 2:** Study characteristics and outcomes among women

Study	Setting	Study population	Study design	Main findings on disclosure	Main findings on violence
Wagman et al., 2015	Uganda Rakai district	Total study population: *N*=11,448 (5337 intervention, 6111 control) general adult population (59% women, 41% men) HIV-positive women: *N*=791 (343 intervention, 448 control)	Cluster randomized controlled trial *N*=4 intervention clusters; 7 control clusters. Assessments took place at baseline, 16 months and 35 months follow-up.	***Among all women:*** Reported disclosure was significantly higher among women in intervention compared to control communities at 35 months (42%, 37%; aPRR=1.15 [95% CI 1.06, 1.24]), but was not significantly different at 16 months. ***Among HIV***+***women:*** Reported disclosure was higher, but not statistically significantly different, among HIV+ women in intervention compared to control communities at 16 months (27%, 25%, *p=*0.34) or 35 months (33%, 29%; *p*=0.24); PRR=1.08 (95% CI 0.82, 1.43).	***Among all women*** **a** ***:*** Reported physical violence was significantly lower among women in intervention compared to control communities at 35 months (12%, 16%; aPRR=0.79 [95% CI 0.67, 0.92]), but was not significantly different at 16 months. Reported sexual violence and forced sex were significantly lower among women in intervention compared to control communities at 35 months (10%, 13%; aPRR=0.80 [CI: 0.67, 0.97]; 8%, 11%; aPRR=0.79 [CI: 0.65, 0.96]), but were not significantly different at 16 months. No significant difference in reported emotional violence at 16 or 35 months.
Maman et al., 2014, and in progress	South Africa Umlazi Township, Durban	Total study population *N*=1480 (733 intervention, 747 control) pregnant women receiving antenatal care HIV-positive women: *N*=571	Individual randomized controlled trial. Assessments took place at baseline (first antenatal care visit), 14 weeks postpartum and 9 months postpartum.	***Among all women*** **a** ***:*** No significant differences in reported disclosure rates among women between intervention and control groups at 14 weeks postpartum.	***Among all women*** **a** ***:*** No significant differences in reported physical, emotional or sexual violence among women between intervention and control groups at 14 weeks postpartum.

PRR: prevalence rate ratio; aPRR: adjusted prevalence rate ratio; CI: confidence interval (all are 95% confidence intervals).

a Data were not available among HIV-positive women.

In the SHARE study, women in the intervention group reported higher rates of HIV serostatus disclosure than women in the control group at the second follow-up; the adjusted prevalence rate ratio (aPRR) was 1.15 (95% CI: 1.06 to 1.24). Women's reports of past-year physical IPV, sexual IPV and forced sex were lower in the intervention group compared to the control group at the second follow-up (35 months after baseline) (physical IPV: aPRR: 0.79, 95% CI: 0.67 to 0.92; sexual IPV: aPRR: 0.80, 95% CI: 0.67 to 0.97; forced sex: aPRR: 0.80, 95% CI: 0.65 to 0.96). Reports of emotional IPV were not statistically significantly different at second follow-up, nor were any outcomes significant at the first follow-up (16 months after baseline).

The SHARE study authors provided additional data for women living with HIV in the sample. These data reflect women with prevalent infection, rather than just those who were newly learning their HIV-positive status (the main target of the safer disclosure intervention), and they reflect all women from the sample in the intervention communities rather than just women who received the safer disclosure intervention. At baseline, there was no difference in disclosure among women living with HIV across study arms. At both follow-ups, a higher proportion of women living with HIV in the intervention group reported disclosing compared to the control group, but this difference was not significant (prevalence rate ratio for entire follow-up period: 1.08; 95% CI: 0.82 to 1.43).

While the SAHAPS study has not yet published disclosure and violence outcomes, the study authors provided additional data showing that there were no significant differences across study arms in either disclosure or IPV at the 14 week postpartum assessment. While the effect of the safer disclosure component cannot be isolated from the effect of the rest of the intervention, the fact that the study saw no measurable effect on disclosure or violence overall may also suggest that the individual components had no measurable effect. The authors also looked at interactions between HIV status, disclosure and IPV among participants in both study arms at follow-up. Among women who disclosed, IPV at 14 weeks postpartum was not significantly different for HIV-positive and HIV-negative women. However, among women who had not disclosed, the odds of reporting IPV at 14 weeks was almost five times higher for HIV-positive women as compared to HIV-negative women.

## Discussion

Despite significant attention to the intersections between HIV and violence as well as international policy consensus on the need to facilitate safer disclosure of HIV serostatus for women who experience or fear violence, we identified only two studies evaluating such interventions globally. Both studies were from sub-Saharan Africa and employed strong randomized designs. However, neither provided clear evidence for the effectiveness of a safer disclosure intervention as they were not designed to isolate the contribution of the safer disclosure components from the broader multiple-component interventions. The evidence base for interventions to facilitate safer disclosure is thus quite limited, and further studies are needed.

For all women, the SHARE study in Uganda reported positive outcomes for disclosure and violence at the second follow-up. Unpublished data among HIV-positive women showed non-significant but positive trends in disclosure. However, these analyses could not distinguish women who had received the enhanced counselling intervention from those who had not or newly diagnosed women from those who had known their HIV-positive status for a long time, both of which would likely attenuate an effect toward the null. In the SAHAPS study in South Africa, unpublished data showed that women who were exposed to the enhanced counselling intervention were no more likely to disclose to their partner or report violence after HIV diagnosis than women who received standard of care HIV testing services. Additional analyses suggested that women who were already at higher risk of IPV – particularly HIV-positive women – chose not to disclose despite, or perhaps because of, the safer disclosure intervention, while women who disclosed were those who already knew it was safe to do so. This interpretation is supported by previous descriptive studies showing that women who have disclosed their status are less likely to have ever experienced violence [30] and a systematic review of male involvement in PMTCT programmes, which found that women who want to test together as couples may be a self-selected group of those already in non-violent relationships [31]. Together, these findings suggest that while IPV continues to be a significant barrier to disclosure for women who fear violence, it has not been adequately addressed in current approaches related to provision of HIV testing, treatment and care services in healthcare settings. There are several options to be considered in such a scenario. One option is that in the absence of specific interventions to respond to violence or promote safety, women who are at risk of violence may be better off being supported in a decision not to disclose their status. Another consideration is to promote safety for women who do want to disclose or who may experience inadvertent disclosure of their status and to do so in line with WHO guidelines. These guidelines recommend training of healthcare providers, especially in HIV testing settings, to identify women who are at risk of IPV and offer a response that includes first-line psychological support including safety planning, addressing immediate needs for physical and mental health and providing referrals to appropriate services that address violence [32].

Both studies included in this review evaluated interventions that integrated screening for IPV into HIV testing services and trained counsellors to facilitate discussions about disclosure based on a woman's risk of violence. HIV testing services provide an opportunity for safer disclosure interventions, but this is not the only potential approach to facilitating safer disclosure for women who experience violence. The 2006 WHO consultation found that programmes jointly addressing the problems of HIV and violence used two broad types of strategies: those addressing violence against women in HIV testing programmes and those addressing HIV-related needs among women who experience violence [22]. While both of the interventions identified in this review integrated violence assessments with HIV services, there may be other interventions addressing violence risk in the context of HIV serostatus disclosure outside of testing that have not been evaluated. Creative ideas include integrating safer disclosure messages into support groups for people living with HIV and training peer counsellors to address disclosure-related violence as part of their work. We encourage programmes currently attempting to intervene to facilitate safer disclosure of HIV status for women who experience or fear violence to share their results in implementation science journals, short report formats or by other means of dissemination to add to the existing evidence base.

In this review, we included all studies that attempted to directly address safer HIV serostatus disclosure for women who experience or fear violence. We did not include studies that may indirectly encourage safer disclosure. For example, many violence prevention programmes that seek to change community norms around violence give women legal rights and protections and give women greater control over financial resources may also have the effect of creating safer environments for women to disclose their serostatus. Similarly, interventions that attempt to reduce HIV-related stigma and discrimination may also reduce the risk of violence for women living with HIV who choose to disclose. Efforts to change community norms that justify or tolerate violence against women and that perpetrate HIV-related stigma are critical to creating enabling environments that reduce violence, which in turn can contribute to facilitating safer disclosure [33]. However, our review focused on direct interventions to facilitate safe HIV disclosure that can be implemented within the context of existing HIV services.

Our findings must be seen in the context of the limitations of our review. While we did not include any language restrictions in our inclusion criteria and searched several online databases that include articles in languages other than English, our search for grey literature only included English-language websites as we were not familiar with any relevant websites in other languages. We may therefore have missed some unpublished evaluations of interventions in other languages.

While HIV serostatus disclosure may lead to many positive outcomes, for many women living with HIV, fear of violence in response to HIV serostatus disclosure is a serious concern. In many settings, health workers are not trained to identify women at risk of violence, provide them with appropriate care and support them in enhancing their safety in relation to health behaviours (e.g. disclosure, safer sex). Moreover, in many settings there are few support services or referral options for women who experience violence generally, regardless of their HIV status. This situation poses a challenge for all health services, including HIV and reproductive health programmes, which are often resource-constrained at the human and financial levels and may not be able to provide comprehensive violence services. Further, as new approaches to HIV testing are expanded as part of efforts to reach the UNAIDS 90-90-90 targets, including community-based and provider-initiated testing, self-testing and testing conducted by lay healthcare providers [34], programmes will need to consider how to achieve safer disclosure of HIV status within the complexities inherent in each of these models [35]. Further research is needed to identify which interventions can best achieve the objective of supporting women living with HIV who experience or fear violence to safely disclose their HIV serostatus – or not to disclose at all, as appropriate – in order to inform programme and policy decisions.

## References

[1] Smith R, Rossetto K, Peterson BL (2008). A meta-analysis of disclosure of one's HIV-positive status, stigma and social support. AIDS Care.

[2] Katz IT, Ryu AE, Onuegbu AG, Psaros C, Weiser SD, Bangsberg DR (2013). Impact of HIV-related stigma on treatment adherence: systematic review and meta-synthesis. J Int AIDS Soc.

[3] Simoni JM, Pantalone DW (2004). Secrets and safety in the age of AIDS: does HIV disclosure lead to safer sex?. Top HIV Med.

[4] Cohen MS, Chen YQ, McCauley M, Gamble T, Hosseinipour MC, Kumarasamy N (2011). Prevention of HIV-1 infection with early antiretroviral therapy. N Engl J Med.

[5] Baeten JM, Donnell D, Ndase P, Mugo NR, Campbell JD, Wangisi J (2012). Antiretroviral prophylaxis for HIV prevention in heterosexual men and women. N Engl J Med.

[6] Medley A, Garcia-Moreno C, McGill S, Maman S (2004). Rates, barriers and outcomes of HIV serostatus disclosure among women in developing countries: implications for prevention of mother-to-child transmission programmes. Bull World Health Organ.

[7] Obermeyer CM, Baijal P, Pegurri E (2011). Facilitating HIV disclosure across diverse settings: a review. Am J Public Health.

[8] WHO, LSHTM, SAMRC (2013). Global and regional estimates of violence against women: prevalence and health effects of intimate partner violence and non-partner sexual violence.

[9] Salamander Trust (2014). Building a safe house on firm ground: key findings from a global values and preferences survey regarding the sexual and reproductive health and human rights of women living with HIV.

[10] Koenig LJ, Moore J (2000). Women, violence, and HIV: a critical evaluation with implications for HIV services. Matern Child Health J.

[11] Gari T, Habte D, Markos E (2010). HIV positive status disclosure among women attending ART clinic at Hawassa University Referral Hospital, South Ethiopia. East Afr J Public Health.

[12] Iliyasu Z, Abubakar IS, Babashani M, Galadanci HS (2011). Domestic violence among women living with HIV/AIDS in Kano, Northern Nigeria. Afr J Reprod Health.

[13] Shamu S, Zarowsky C, Shefer T, Temmerman M, Abrahams N (2014). Intimate partner violence after disclosure of HIV test results among pregnant women in Harare, Zimbabwe. PLoS One.

[14] Tam M, Amzel A, Phelps BR (2015). Disclosure of HIV serostatus among pregnant and postpartum women in sub-Saharan Africa: a systematic review. AIDS Care.

[15] Gourlay A, Birdthistle I, Mburu G, Iorpenda K, Wringe A (2013). Barriers and facilitating factors to the uptake of antiretroviral drugs for prevention of mother-to-child transmission of HIV in sub-Saharan Africa: a systematic review. J Int AIDS Soc.

[16] Schafer KR, Brant J, Gupta S, Thorpe J, Winstead-Derlega C, Pinkerton R (2012). Intimate partner violence: a predictor of worse HIV outcomes and engagement in care. AIDS Patient Care STDs.

[17] Mugavero MJ, Raper JL, Reif S, Whetten K, Leserman J, Thielman NM (2009). Overload: impact of incident stressful events on antiretroviral medication adherence and virologic failure in a longitudinal, multisite human immunodeficiency virus cohort study. Pyschosom Med.

[18] Cohen MH, Cook JA, Grey D, Young M, Hanau LH, Tien P (2004). Medically eligible women who do not use HAART: the importance of abuse, drug use, and race. Am J Public Health.

[19] Mugavero M, Ostermann J, Whetten K, Leserman J, Swartz M, Stangl D (2006). Barriers to antiretroviral adherence: the importance of depression, abuse, and other traumatic events. AIDS Patient Care STDs.

[20] Machtinger EL, Haberer JE, Wilson TC, Weiss DS (2012). Recent trauma is associated with antiretroviral failure and HIV transmission risk behavior among HIV-positive women and female-identified transgenders. AIDS Behav.

[21] Mulrenan C, Colombini M, Howard N, Kikuvi J, Mayhew SH (2015). Exploring risk of experiencing intimate partner violence after HIV infection: a qualitative study among women with HIV attending postnatal services in Swaziland. BMJ Open.

[22] WHO (2006). Addressing violence against women in HIV testing and counselling. A meeting report.

[23] Moher D, Liberati A, Tetzlaff J, Altman DG (2009). Preferred reporting items for systematic reviews and meta-analyses: the PRISMA statement. Ann Intern Med.

[24] WHO (2014). HIV and adolescents: Guidance for HIV testing and counselling and care for adolescents living with HIV.

[25] Kennedy CE, Fonner VA, Armstrong KA, O'Reilly KR, Sweat MD (2015). Increasing HIV serostatus disclosure in low- and middle-income countries: a systematic review of intervention evaluations. AIDS.

[26] Higgins JPT, Green S Cochrane handbook for systematic reviews of interventions [Internet].

[27] Wagman JA, Gray RH, Campbell JC, Thoma M, Ndyanabo A, Ssekasanvu J (2015). Effectiveness of an integrated intimate partner violence and HIV prevention intervention in Rakai, Uganda: analysis of an intervention in an existing cluster randomised cohort. Lancet Glob Health.

[28] Maman S, Moodley D, McNaughton-Reyes HL, Groves AK, Kagee A, Moodley P (2014). Efficacy of enhanced HIV counseling for risk reduction during pregnancy and in the postpartum period: a randomized controlled trial. PLoS One.

[29] Wagman JA, King EJ, Namatovu F, Kiwanuka D, Kairania R, Semanda JB (2015). Combined intimate partner violence and HIV/AIDS prevention in rural Uganda: design of the SHARE intervention strategy. Health Care Women Int.

[30] Makin JD, Forsyth BW, Visser MJ, Sikkema KJ, Neufeld S, Jeffery B (2008). Factors affecting disclosure in South African HIV-positive pregnant women. AIDS Patient Care STDS.

[31] Brusamento S, Ghanotakis E, Tudor Car L, van-Velthoven MH, Majeed A, Car J (2012). Male involvement for increasing the effectiveness of prevention of mother-to-child HIV transmission (PMTCT) programmes. Cochrane Database Syst Rev.

[32] WHO (2013). Responding to intimate partner violence and sexual violence against women: WHO clinical and policy guidelines.

[33] Ellsberg M, Arango DJ, Morton M, Gennari F, Kiplesund S, Contreras M (2015). Prevention of violence against women and girls: what does the evidence say?. Lancet.

[34] WHO (2015). Consolidated guidelines on HIV testing services.

[35] Kumwenda M, Munthali A, Phiri M, Mwale D, Gutteberg T, MacPherson E (2014). Factors shaping initial decision-making to self-test amongst cohabiting couples in urban Blantyre, Malawi. AIDS Behav.

# Sub article: A pressing need to respond to the needs and sexual and reproductive health problems of adolescent girls living with HIV in low- and middle-income countries

**DOI:** 10.7448/IAS.18.6.20297

**Published:** 2015-12-01

**Authors:** Venkatraman Chandra-Mouli, Alice Armstrong, Avni Amin, Jane Ferguson

**Affiliations:** 1 Department of Reproductive Health and Research, World Health Organization, Geneva, Switzerland; 2 Department of HIV/AIDS, World Health Organization, Geneva, Switzerland; 3 Department of Maternal, Newborn, Child and Adolescent Health, World Health Organization, Geneva, Switzerland

**Keywords:** adolescents living with HIV, adolescent girls, sexual and reproductive health

## Abstract

**Introduction:**

This commentary provides the rationale and makes a call for greater investment and effort to meet the sexual and reproductive health (SRH) problems of adolescent girls living with HIV in low- and middle-income countries (LMIC).

**Discussion:**

Adolescent girls in LMIC are at a greater risk of acquiring HIV infection than their male peers. They also face a number of other serious SRH problems – early pregnancy, pregnancy- and childbirth-related complications, unsafe abortions, sexual abuse and intimate partner violence and sexually transmitted infections. While many LMIC have made notable progress in preventing HIV in children and adults and in improving the access of these population groups to HIV treatment and care, adolescents in general and adolescent girls in particular have not received the same effort and investment.

**Conclusions:**

Much more needs to be done to implement proven approaches to prevent new HIV infections in adolescent girls in LMIC and to meet the needs of those living with HIV.

## Introduction

In the 15 years since the United Nations Millennium Declaration, the world has made tremendous progress in reducing the number of new HIV infections and in reducing deaths in those living with HIV [1,2].

Relative to this improvement, there has been limited progress in preventing new HIV infections in adolescents, especially in low- and middle-income countries (LMIC) [1,2]. This is both because the interventions that have been used have been shown to be of limited effectiveness [3] and because HIV prevention efforts in adolescents have not received the investment and effort needed [4]. Many LMIC have made substantial progress in diagnosing and treating children and adults with HIV infection. Lack of concerted efforts directed at adolescents has meant that LMIC have made relatively less progress in reaching this population group with HIV testing and counselling, as well as treatment and care services [1,5,6].

While HIV infections and HIV-related mortality and morbidity occur in both adolescent boys and girls, the number of new infections and the number of individuals living with HIV are far higher in the latter [1,2,5,6]. This is primarily because biological as well as social, cultural and economic factors increase girls’ vulnerability to HIV infection and to its health and social consequences. While acknowledging that boys and young men need concerted attention too, the focus of this paper is on adolescent girls and young women, with a special focus on those living with HIV. It provides the rationale and makes a call for greater investment and effort to meet the sexual and reproductive health (SRH) problems of adolescent girls living with HIV in LMIC.

## Discussion

This paper begins by describing the SRH problems that adolescent girls face. It then discusses the limited progress that has been made in preventing these problems, responding to them when they occur and especially in addressing the social, cultural and economic drivers of these problems. It then discusses the need to increase access to HIV testing and counselling for adolescent girls and to treatment and care for those living with HIV.

## SRH problems in adolescent girls

While most adolescents make the transition to adulthood in good health, not all are so fortunate. They face SRH problems; in endemic countries, HIV is an important one of them.

Globally, new HIV infections in all age groups declined by 38% since 2001 [6]. New HIV infections among adolescents declined even more sharply, by 43%. However, a substantial number of HIV infections – an estimated 250,000 new infections – occurred in adolescents globally in 2012 (range 210,000 to 290,000). Progress was uneven across different regions. For example, while new infections have decreased substantially in Eastern and Southern Africa, they have remained stable in Asia and the Pacific [1,5,6]. Adolescent girls are affected more than their male peers; 64% of the new infections were in girls aged 15 to 19 [1,5,6]. Finally, data from the few available studies on adolescent girls in key populations (Note: Key populations (also referred to as *most-at-risk populations*) are people who inject drugs, gay men and other men who have sex with men, transgender persons and sex workers. They are disproportionately infected with HIV compared to the general population.) suggest that they have significantly higher HIV prevalence and other sexually transmitted infections (STIs) than their peers in the general population [7,8].

Adolescent girls are affected by a number of other SRH problems. An estimated 16 million girls aged 15 to 19 and some one million girls less than 15 years give birth every year – most in LMIC [9,10]. Complications during pregnancy and childbirth are the second leading cause of death for 15 to 19 year-old girls globally (after road traffic injuries) [9]. Adolescent pregnancy, especially when it occurs in early adolescence, is associated with higher rates of maternal mortality and morbidity [11]. It is also associated with increased risks of mortality and morbidity in newborns and children [12]. Of the estimated 22 million unsafe abortions that occur every year, 15% occur among girls aged 15 to 19 [13]. Adolescent girls are at risk of childhood sexual abuse in homes and community settings such as schools, from influential adults around them. Globally, 1 in 10 girls under the age of 20 have been sexually abused [14]. Additionally, an estimated 30% of girls aged 15 to 19 experience intimate partner violence [15]. The WHO estimates that every year 500 million people become ill with one of four curable STIs – chlamydia, gonorrhoea, syphilis and trichomoniasis. In addition there is a huge burden of viral STIs. The WHO also estimates that more than 530 million people are infected with the herpes simplex virus, which causes genital herpes, and more than 290 million women with the human papilloma virus, which causes genital warts and cervical cancer. The available data is not disaggregated by age and so we do not have global estimates for STIs in adolescents. However, in most populations higher levels of infection are noted in young populations [16].

Globally, there are an estimated 35 million people living with HIV. In 2013, an estimated 1.5 million died of AIDS-related illnesses. This figure represents a 35% decline since the peak of 2.4 million deaths, recorded in 2005. AIDS-related deaths, however, have not decreased among adolescents aged 10 to 19 [2]. At the end of 2013, an estimated 2.1 million adolescents (aged 10 to 19 years) were living with HIV; with the majority in sub-Saharan Africa [6]. This estimate includes both perinatally infected children surviving into adolescence as well as individuals infected in the second decade of their lives. In 2013, an estimated 120,000 adolescents aged 10 to 19 years (range 100,000 to 130,000) died of AIDS-related illnesses [1,5]. AIDS remains the number one killer of adolescents in sub-Saharan Africa [2].

## Meeting the SRH information and service needs of all adolescent girls and of those living with HIV

In many contexts, adolescent girls are ill-informed about their bodies and their health and are unprepared for the changes they are experiencing and the challenges they could face. For example, only 30% of young women aged 15 to 24 in sub-Saharan Africa countries (with available data) had comprehensive, correct knowledge of HIV transmission in 2014. This represents an increase of less than 10% in four years [2]. Improving adolescent girls’ knowledge and understanding of SRH and building their life skills to take charge of their health is a crucial step in meeting their health needs and fulfilling their rights [17]. As girls move from older childhood into and through adolescence and then into early adulthood, they need sexuality education that responds to their developmental stages and circumstances and evolves with their changing needs. The sexuality education they receive should be comprehensive; it should both include and go beyond information on sexuality, reproduction and SRH problems and how to avoid them [18,19].

Adolescent girls living with HIV must learn what all girls need to. In addition they must learn to live with a chronic illness and to deal with the emotional and psychological issues associated with the knowledge that HIV is a highly stigmatized transmissible infection. This aspect often has an enormous impact on their sexual health and relationships. Adolescent girls living with HIV do not lose their desire for sex or to have families. Some have not yet had sex but hope/intend to do so in the future and have questions and concerns. Many others have had sex and have unanswered questions and concerns about infecting others, disclosing their status to their partners and having sex and children safely. They need information and counselling that responds to these needs [20,21].

Almost universally, adolescent girls lack access to the SRH commodities (such as condoms and other contraceptives) that they need to protect themselves or to the health services they need (e.g. diagnosis and treatment of STIs, HIV testing and counselling, treatment and care). Restrictive laws and policies in many countries forbid the provision of these services to unmarried adolescents, those below the age of majority. Often adolescent girls do not know where and how to obtain the health services they need. Even when they are able to obtain health services, they are often reluctant to do so because of fears about privacy, confidentiality, of being judged and of being treated with disrespect [17]. And as a study by Nalwadda *et al*. in Uganda highlighted, when they do seek care, adolescent girls say that they are treated with disrespect or are even turned away by providers who refuse to offer them services [22].

Adolescents living with HIV face an additional burden. Stigma and discrimination against people living with HIV and key populations in healthcare settings continue to undermine HIV responses [23]. Adolescent girls can be labelled as promiscuous or discriminated against by not being provided the health services they need [23]. Further, in some settings, they can be coerced into procedures such as sterilization or pregnancy termination [24].

Whether or not they are living with HIV, adolescent girls have a right to a healthy, enjoyable and safe sex life. SRH services should enable them to reduce or avoid health problems and should enable those who experience health problems to recover [25,26]. Countries should eliminate medical and social restrictions to the provision of SRH services including contraceptives to adolescent girls and should support and enable them to obtain commodities and services that are appropriate to their needs and preferences through delivery mechanisms that are acceptable to them [27]. Health services should also be ready to provide those girls who are pregnant with quality antenatal care including linkages to prevention of mother-to-child transmission of HIV care or to help in obtaining a safe abortion where this is permitted by law. Effective care during childbearing is important to ensuring the survival of mothers and their babies and the prevention of mother-to-child transmission and other problems such as fistulas resulting from obstructed labour. Adolescent girls living with HIV need access to contraceptive services including emergency contraception, safe abortion services and services to prevent mother-to-child transmission of HIV, if they get pregnant and want to continue with their pregnancies. They also need information and services to avoid STIs and diagnosis and treatment for STIs when they occur.

## Addressing the social, cultural and economic drivers of SRH problems in all adolescent girls and in those living with HIV

In several settings, adolescent girls engage in sexual relationships with men who are several years older than them. These relationships entail the exchange of sex for essentials (such as food and school fees) as well as non-essential ones (such as mobile phones). Cross-generational sexual relationships are a key driver of HIV risk among adolescent girls. To respond to this, a number of research studies and projects have been undertaken to test approaches to reduce the vulnerabilities of adolescent girls through economic empowerment interventions such as waivers of school fees, cash transfers and microfinance schemes. There is a growing knowledge base in this area [28].

There is also growing research evidence and project experience in preventing sexual coercion of girls [29,30]. This endeavour requires a multifaceted and multilevel approach. At the wider societal level, laws that forbid violence against women and girls should be passed and enforced effectively. Together with this action, efforts are needed to develop social norms that are intolerant of such violence. At the community level, steps should be taken to protect girls and women from physical and emotional violence at home, as well as sexual harassment and coercion in educational institutions and other community settings. These efforts should include activities directed at boys and men to address masculine norms that encourage boys and young men to take sexual risks to prove their manhood and to assert their masculinity by coercing girls and women to have sex. Adolescent girls and boys should be taught that violence is an unacceptable way to resolve conflicts. Moreover, boys and young men should be taught that sexual relationships should be based on equity and respect [29,30].

Studies from many countries point to the common occurrence of forced sex involving adolescent girls and the HIV risk associated with it [31]. There is a dearth of information on whether adolescent girls with HIV experience coerced sex or pressure to have transactional sex. However, such a situation is not unlikely. Given this information, adolescent girls living with HIV should be included in empowering interventions directed at girls uninfected with HIV.

## Increasing access to HIV testing and counselling for adolescent girls and to treatment and care for those living with HIV

As indicated earlier, there are an estimated 1.2 million adolescents living with HIV, with 70 to 80% living in sub-Saharan African. Many of the latter are unaware of their HIV status [2,5]. While there are currently no estimates of antiretroviral coverage for adolescents, the available evidence suggests higher rates of loss to follow-up, poor adherence and increased requirement for psychosocial support in this age group [32–36]. Studies have also pointed to gaps and weaknesses in health services to address the SRH needs of adolescents living with HIV [37–39]. For example, a Kenyan study of adolescents living with HIV reported low rates of contraceptive use (66%). Of those sexually active female respondents, 68% had already been pregnant and three-quarters of those were unintended pregnancies [38]. Additionally, higher mother-to-child transmission risk was seen among infants of HIV-positive adolescent mothers compared to adult mothers, with a 1.7-fold increased risk compared to adult mothers in one study in KwaZulu Natal, South Africa [39]. Adolescent girls from key population groups are also often subject to significant levels of stigma, discrimination, violence, increased risk for criminalization and incarceration and social isolation. These further exclude them from essential platforms for support and access to SRH and other prevention or treatment services [23].

## Looking back at the global response to meeting the needs and fulfilling the rights of adolescent girls, 21 years since the International Conference on Population and Development, and 15 years since the United Nations Millennium Declaration

In 2014, the world commemorated the twentieth anniversary of the International Conference on Population and Development (ICPD). Reviews of progress in the implementation of adolescent sexual and reproductive health policies and programmes over the two decades since the landmark conference noted that progress was limited and patchy [40]. The Millennium Development Goals report for 2015 also points to limited progress made in adolescents in relation to Goals 5 and 6 [2].

A review of the ICPD's achievements that focused on adolescent girls echoed these findings: “… many countries have yet to make significant progress in delaying marriage and childbearing, reducing unintended childbearing, narrowing gender disparities that put girls at risk of poor sexual and reproductive health outcomes, expanding health awareness or enabling access to sexual and reproductive health services” [41].

Given this backdrop, UNAIDS has called for a much stronger collective response to preventing HIV and HIV-related mortality in adolescents [42].

Two recent reviews stress the need to strengthen HIV prevention efforts directed at adolescent girls in LMIC, by using tailor-made combinations of biomedical, behavioural and structural interventions to address the web of individual and environmental factors that increase their vulnerability to HIV. Both stress the importance of developing more effective prevention options [28,43].

HIV prevention efforts must be implemented with efforts to improve the provision of, access to and uptake of quality health services (including SRH services, HIV testing counselling and HIV treatment and care services) to adolescent girls living with HIV. Two complementary approaches are needed. Firstly, lack of the required competencies and moralistic attitudes or values stand in the way of health workers providing adolescents living with HIV with the health services they need. Training, value clarification and ongoing support can enable them to make the contributions they need to. Two important starting points are making an assessment of sexual activity and providing full and accurate information [44]. Secondly, in most LMIC, health services are either not available or are not geared towards adolescents. Existing services should be made more welcoming (accessible and acceptable) to adolescents living with HIV – including those in key populations. Integrating SRH services with other health services, decentralizing their provision away from hospitals to clinics in the adolescent's neighbourhood and reaching out with services in the community can extend their range [45].

## Conclusions

Limited progress has been made in preventing SRH problems, notably HIV infections, in adolescent girls and in preventing HIV-related mortality in this group. This inadequate improvement, especially in relation to the progress made in other groups, is unacceptable. Much more needs to be done to prevent HIV in girls and to enable those living with HIV to survive and live to their full potential.

## Author information

Venkatraman Chandra-Mouli works as a scientist on adolescent sexual and reproductive health in the WHO's Department of Reproductive Health and Research, which includes the Special Programme of Research, Development and Research Training in Human Reproduction. His work includes supporting efforts to build the epidemiologic and evidence base and supporting countries to translate this evidence into action. He has led or contributed substantially to a number of the WHO's publications on adolescent health, including evidence reviews, policy and programmatic guidance, advocacy documents, training and self-learning materials and evaluation tools. As part of his work in the WHO, he has contributed to the work of national-level bodies and front-line organizations, in many different social, cultural and economic settings.

Alice Armstrong is a nurse practitioner and prescriber working in HIV and sexual reproductive health with a particular focus on adolescents. She is currently an affiliated consultant in the HIV/AIDS Department in the WHO in Geneva. She has coordinated the development of the HIV Testing and Counselling online implementation tool and the young key populations’ technical briefs; she has also worked on the adolescent components of the consolidated key population and antiretroviral guidance. She is currently working on adolescent antiretroviral treatment and adolescent male circumcision. Before joining the WHO, she was the adolescent programme coordinator for the Children's HIV Association, South Africa, providing technical support to the Ministry of Health on adolescent HIV service delivery.

Jane Ferguson is a scientist in the Maternal, Newborn, Child and Adolescent Health Department of the WHO in Geneva. She is the focal point for adolescent health and responsible for research and development related to adolescents in that Department. Jane has held a variety of positions in various configurations of the WHO over the past several decades, devoted to moving adolescent health onto the public health agenda. During these years she has worked with ministries of health and civil society organizations, including youth organizations, United Nations partners and technical and funding agencies in countries in all regions of the world. She has led adolescent health strategy development, defined research priorities and developed programme support tools, including recent guidelines on adolescent reproductive health outcomes and HIV prevention, treatment and care.

Avni Amin is a technical officer for violence against women at the Department of Reproductive Health and Research, WHO Geneva. She has worked on issues of gender equality, violence against women and their linkages to sexual and reproductive health and HIV for the last 18 years.

## References

[1] UNAIDS (2014). Global report: UNAIDS report on the global AIDS epidemic.

[2] United Nations (2015). Millennium Development Goals Report: 2015.

[3] Michielsen K, Chersich M, Luchters S, de Koker P, Van Rossern R, Temmerman M (2010). Effectiveness of HIV prevention for youth in sub-Saharan Africa: systematic review and meta-analysis of randomized and nonrandomized trials. AIDS.

[4] Independent Expert Review Group (2014). The Second Report of the independent Expert Review Group (iERG) on Information and Accountability for Women's and Children's Health.

[5] UNICEF (2014). Children, adolescents and AIDS. 2014 statistical update.

[6] UNAIDS (2014). The gap report.

[7] Wilson EC, Garofalo R, Harris RD, Herrick A, Martinez M, Martinez J (2009). Transgender female youth and sex work: HIV risk and a comparison of life factors related to engagement in sex work. AIDS Behav.

[8] Busza JR, Balakireva OM, Teltschik A, Bondar TV, Sereda YV, Meynell C (2011). Street-based adolescents at high risk of HIV in Ukraine. J Epidemiol Community Health.

[9] WHO (2014). Health for the world's adolescents: a second chance in the second decade.

[10] Neal S, Matthews Z, Frost M, Fogstad H, Camacho AV, Laski L (2012). Child bearing in adolescents aged 12–15 years in low resource countries: a neglected issue. New estimates from demographic and household surveys in 42 countries. Acta Obstet Gynecol Scand.

[11] Nove A, Matthews Z, Neal S, Camacho AV (2014). Maternal mortality in adolescents compared with women of other ages. Evidence from 144 countries. Lancet Glob Health.

[12] Ganchimeg T, Ota E, Morisaki N, Laopaiboon M, Lumbiganon P, Zhang J (2014). Pregnancy and childbirth outcomes among adolescent mothers: a World Health Organization multi-country study. Br J Gynaecol Obstet.

[13] Shah I, Ahman E (2012). Unsafe abortion differentials in 2008 by age and developing country region: high burden among young women. Reprod Health Matters.

[14] UNICEF (2014). Hidden in plain sight. A statistical analysis of violence against children.

[15] WHO (2013). Global and regional estimates of violence against women: prevalence and health effects of intimate partner violence and non-partner sexual violence.

[16] WHO (2013). Sexually transmitted infections. Fact sheet no. 110. November 2013.

[17] Temin M, Levine R (2009). Start with a girl. A new agenda for global health.

[18] UNESCO (2009). International technical guidance on sexuality education.

[19] UNFPA (2014). Operational guidance for comprehensive sexuality education. A focus on human rights and gender.

[20] WHO (2010). Strengthening the health sector response to care, support, treatment and prevention for young people living with HIV.

[21] WHO (2010). Integrated management of adolescent and adult illnesses. One-day orientation on adolescents living with HIV. Participant manual and facilitator guide.

[22] Nalwadda G, Mirembe N, Tumwesigye N, Josphat B, Faxelid E (2011). Constraints and prospects for contraceptive service provision to young people in Uganda: providers’ perspectives. BMC Health Serv Res.

[23] WHO (2014). Global update on the health sector response to HIV.

[24] WHO (2014). Eliminating forced, coercive and otherwise involuntary sterilization. An interagency statement. OHCHR, UN Women, UNAIDS, UNDP, UNFPA, UNICEF and WHO.

[25] WHO (2006). Preventing HIV in young people. A systematic review of the evidence from developing countries.

[26] WHO (2011). WHO Guidelines on preventing early pregnancy and poor reproductive outcomes in adolescents in developing countries.

[27] WHO (2012). From evidence to policy: expanding access to family planning. Expanding access to contraceptive services for adolescents.

[28] Hardee K, Gay J, Croce-Galis M, Afari-Dwamena NA (2014). What HIV programs work for adolescent girls?. J Acquir Immune Defic Syndr.

[29] Heise L (2011). What works to prevent partner violence: an evidence overview.

[30] Amin A, Chandra-Mouli V (2014). Empowering girls: developing egalitarian gender norms and relations to end the cycle of violence. Reprod Health.

[31] Stockman JK, Lucea MB, Campbell JC (2013). Forced sexual initiation, sexual intimate partner violence and HIV risk in women: a global review of the literature. AIDS Behav.

[32] Koech E, Teasdale C, Wang C, Fayorsey R, Alwar T, Mukui IN (2014). Characteristics and outcomes of HIV infected youth and young adolescents enrolled in HIV care in Kenya. AIDS.

[33] Auld A, Agolory S, Shiraishi R, Wabwire-Mangen F, Kwesigabo G, Mulenga M (2014). Antiretroviral therapy enrolment characteristics and outcomes among HIV-infected adolescents and young adults compared with older adults – seven African countries, 2004–2013. Mort Morb Wkly Rev.

[34] Nachega J, Hislop M, Nguyen H, Dowdy DW, Chaisson RE, Regensberg L (2009). Antiretroviral therapy adherence, virologic and immunologic outcomes in adolescents compared with adults in southern Africa. J AIDS.

[35] Chandwani S, Koenig L, Sill A, Abramowitz S, Conner LC, D'Angelo L (2012). Predictors of antiretroviral medication adherence among a diverse cohort of adolescents with HIV. J Adolesc Health.

[36] Lamb R, Fayorsey R, Nuwagaba-Birbonwoha H, Viola V, Mutabazi V, Alwar T (2014). High attrition before and after ART initiation among youth (15–24 years of age) enrolled in HIV care. AIDS.

[37] Baryamuta R, Baingana F (2011). Sexual and reproductive health needs and rights of young people with perinatally acquired HIV in Uganda. Afr J Health Sci.

[38] Obare F, van der Kwaak A, Adieri B, Owuor D, Okoth S, Musyoki S (2010). HIV-positive adolescents in Kenya: access to sexual and reproductive health services. KIT Development Policy and Practice.

[39] Horwood C, Butler LM, Haskins L, Phakathi S, Rollins N (2013). HIV-infected adolescent mothers and their infants: low coverage of HIV services and high risk of HIV transmission in KwaZulu-Natal, South Africa. PLoS One.

[40] United Nations (2014). Report of the Secretary General, United Nations on ‘Assessment of the status of implementation of Programme of Action of the ICPD’.

[41] Santhya KG, Jejeebhoy SJ (2015). Sexual and reproductive health and rights of adolescent girls: evidence for low- and middle-income countries. Global Public Health.

[42] UNAIDS (2015). All in: end adolescent AIDS.

[43] Dellar RC, Dlamini S, Abdool Karim Q (2015). Adolescent girls and young women: key populations for HIV epidemic control. J Int AIDS Soc.

[44] WHO (2014). Adolescent HIV testing, counselling and care. Implementation guidance for health providers and planners.

[45] WHO (2015). Global standards for quality health care services for adolescents. A guide to implement a standards-driven approach to improve the quality of health services for adolescents.
